# Theoretical Study on the Structural-Function Relationship of Manganese(III)-Iodosylarene Adducts

**DOI:** 10.3389/fchem.2020.00744

**Published:** 2020-08-20

**Authors:** Dongru Sun, Xiaolu Chen, Lanping Gao, Yufen Zhao, Yong Wang

**Affiliations:** School of Material Science and Chemical Engineering, Institute of Drug Discovery Technology, Ningbo University, Ningbo, China

**Keywords:** manganese(III)–iodosylarene, sulfoxidation, C-H bond activation, mechanism, DFT

## Abstract

Metal-iodosylarene complexes have been recently viewed as a second oxidant alongside of the well-known high-valent metal-oxo species. Extensive efforts have been exerted to unveil the structure-function relationship of various metal-iodosylarene complexes. In the present manuscript, density functional theoretical calculations were employed to investigate such relationship of a specific manganese-iodosylbenzene complex [Mn^III^(TBDAP)(PhIO)(OH)]^2+^ (**1**). Our results fit the experimental observations and revealed new mechanistic findings. **1** acts as a stepwise 1e+1e oxidant in sulfoxidation reactions. Surprisingly, C-H bond activation of 9,10-dihydroanthracene (DHA) by **1** proceeds via a novel ionic hydride transfer/proton transfer (HT/PT) mechanism. As a comparison to **1**, the electrophilicity of an iodosylbenzene monomer PhIO was investigated. PhIO performs concerted 2e-oxidations both in sulfoxidation and C-H activation. Hydroxylation of DHA by PhIO was found to proceed via a novel ionic and concerted proton-transfer/hydroxyl-rebound mechanism involving 2e-oxidation to form a transient carbonium species.

## Introduction

**Scheme 1:**
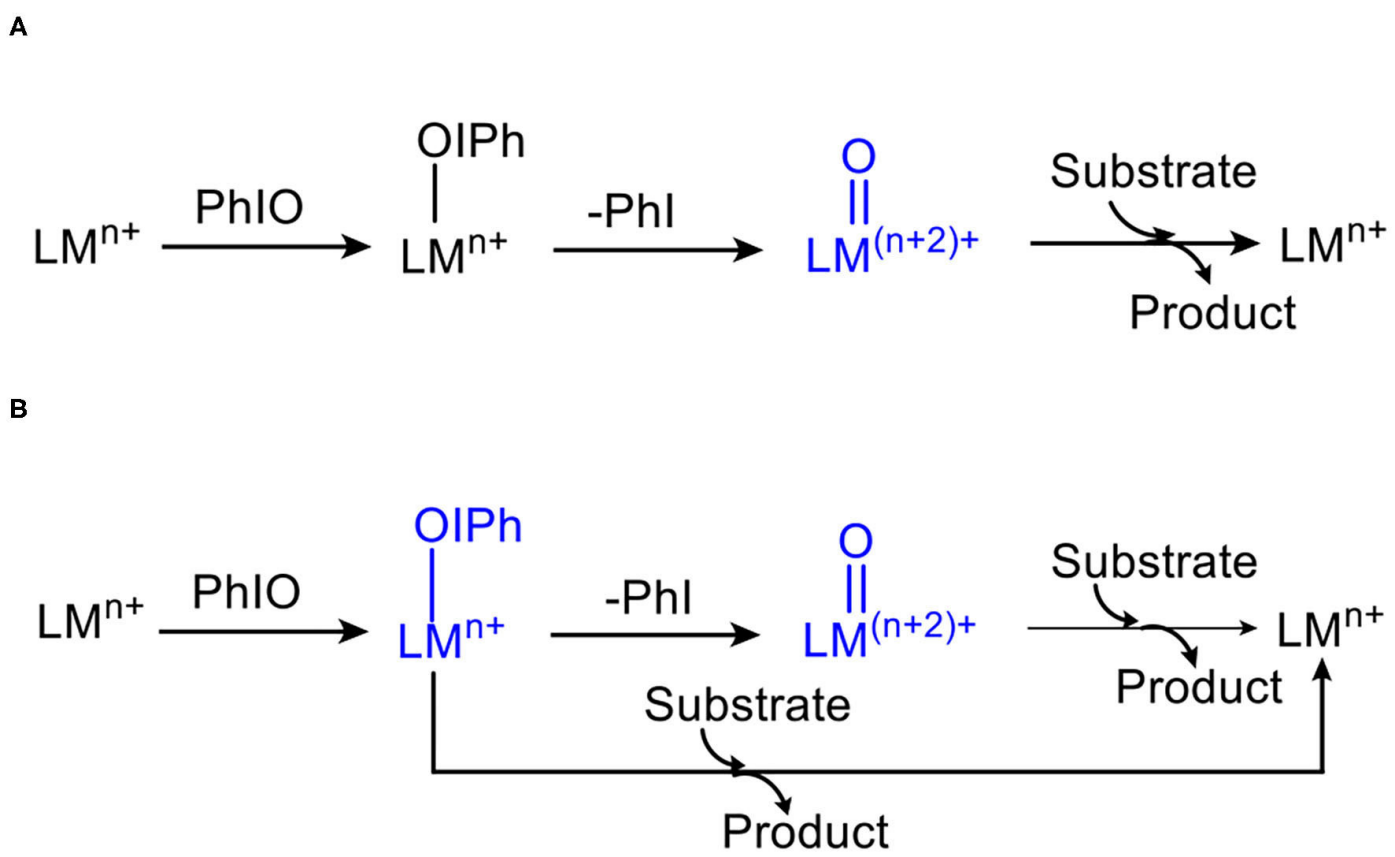
The controversy of **(A)** one-oxidant mechanism and **(B)** multiple-oxidant mechanism.

Iodosylarenes have been frequently employed as terminal oxidants in the synthesis of metal-oxo species, as well as in the catalytic oxidation of organic substrates including C–H hydroxylation and O-atom transfer to substrates (Zhdankin and Stang, 2002, 2008; Yoshimura and Zhdankin, 2016). Generally, the insoluble iodosylarenes bind to an heme or non-heme metal core and produce the metal-iodosylarene complexes (Scheme 1A) (Macikenas et al., 2000). Such metal-iodosylarene intermediates are unstable and ready to cleave into high-valent metal-oxo species and iodoarenes. The nascent high-valent metal-oxo species are believed to be the sole oxidants in the various oxygenation reactions (Groves et al., 1979; Groves and Nemo, 1983). Extensive experimental observations support this one-oxidant mechanism, which becomes a widely accepted mechanism in oxygenation chemistry (Shaik et al., 2005). However, in the 1990s, Valentine and co-workers found that epoxidation of olefin was significantly enhanced when redox-innocent metals were added in the oxidation system involving idosylbenzene (Nam and Valentine, 1990; Yang et al., 1990). This experimental result drops the one-oxidant mechanism into doubt. In 2000, Collman, Brauman and co-workers reported the oxidation rate ratios for each pair of substrates varied when different iodosylarenes were employed as terminal oxidants (Collman et al., 2000). In 2002, Nam and co-workers suggested that the ratio of stereoisomers in olefin epoxidation reactions catalyzed by porphyrin iron complex depended on the terminal oxidant or counterions, and proposed the multiple-oxidant mechanism (Scheme 1B) (Nam et al., 2002). In this mechanism, both metal-iodosylarene adducts and high-valent metal-oxo complexes are potential oxidants to oxidize the substrates to the products. Nam and co-workers have presented various spectroscopic (UV-Vis, Raman, etc.) and enantioselectivity evidences to support this mechanism (Hong et al., 2014; Wang et al., 2015).

Many evidences supporting the multiple-oxidant mechanism also come from other famous groups. Hill et al. reported iodosylbenzene adducts of a manganese porphyrin complex trans-[Mn^IV^(por)(OI(OAc)Ph)_2_] as a precursor to active high-valent manganese–oxo species (Smegal and Hill, 1983). Fujii and his workers crystalized another Mn(IV)-iodosylarene complex trans-[Mn^IV^(salen)(MesIO)^2^Cl]^+^ (Wang et al., 2012), and clarified that the reactivity and selectivity of iodosylarene adducts depended on steric and electronic properties of substituents on iodine(III) of the coordinated iodosylarenes (Wang et al., 2013). The spectroscopic evidence for a manganese iodosylarene porphyrin adduct [Mn(TDCPP)(ArIO)]^+^ was also reported by Lei (Guo et al., 2012). Meanwhile, other metal-iodosylarene complexes with different metals have been crystalized, such as the iron complex [Fe^III^(tpena)(OIPh)]^+^ by Mckenzie (Lennartson and Mckenzie, 2012), the rhodium complex [Rh^III^Cp^*^(ppy)(^s^PhIO)]^+^ by Templeton (Turlington et al., 2014), and the cobalt complex [Co^II^TptBu(sPhIO)]^+^ by Anderson (Hill et al., 2018). In addition to normal transition-metal complex, the lanthanide complexes, the Ce-iodosylbenzene one [Ce^IV^(LOEt)_2_(OI(Cl)Ph)_2_] and the Ce-iodylbenzene one [Ce^IV^(LOEt)_2_(OI(O)ClPh)_2_] were reported by Leung et al. (Au-Yeung et al., 2016).

Recently, Cho and his co-workers crystallized the first X-ray crystal structure of a mononuclear manganese-iodosylarene complex, [Mn^III^(TBDAP)(OIPh)(OH)]^2+^ (**1**, TBDAP = *N, N*-ditert-butyl-2,11-diaza-[3.3](2,6)-pyridinophane), which is capable of conducting various oxidation reactions, such as C–H bond activation, sulfoxidation, and epoxidation (Jeong et al., 2018). In these reactions, **1** exhibits similar and even higher electrophilic oxidation power comparing to highly reactive manganese(IV)-oxo complexes. A consecutive Mn(III)(OIPh)(OH)/Mn(IV)(OH)_2_ conducting mechanism (Jeong et al., 2018) was proposed for the hydrogen abstraction reactions and a direct oxygen atom transfer mechanism (DOT) (Li et al., 2007; Shaik et al., 2010) was postulated for the sulfoxidation and epoxidation reactions. However, no further theoretical study is performed to support such mechanism. Herein, we presented the first theoretical investigation to the structure-reaction relationship of **1** shown in oxidative C-H bond activation and sulfoxidation. Our results demonstrate that *such Mn(III)-OIPh complex* ***1****prefers to be stepwise 1e*+*1e oxidant* in sulfoxidations, oxygen transfer occurs via the electron transfer followed by oxygen transfer (ETOT) mechanism proposed by Watanabe and Baciocchi (Goto et al., 1999; Baciocchi et al., 2003), not the DOT mechanism. *Surprisingly, in the C-H bond activation mediated by* ***1****, a new ionic hydride transfer/proton transfer (HT/PT) mechanism is preferred* (Li et al., 2016; Geng et al., 2017, 2019; Schwarz et al., 2017).

## Theoretical Methods

Coordinates of [Mn^III^(TBDAP)(OIPh)(OH)]^2+^ was obtained from the Cambridge Crystallographic Data Center with deposition number CCDC-1868139. Thioanisole and 9,10-Dihydroanthracene (DHA) were employed as the substrates in the mechanistic study of sulfoxidation and C–H bond activation, respectively. Density functional theory (DFT) calculations were carried out using the Gaussian 16 suite of quantum chemical programs (Frisch et al., 2016). The spin-unrestricted functional B3LYP-D3(BJ) (Becke, 1992a,b, 1993) with the addition of Grimme's D3 dispersion and Becke–Johnson damping (Grimme et al., 2010, 2011) was used. Such functional has been extensively verified to be accurate and efficient in many transition metal-containing reaction systems (Yang et al., 2016, 2019). Two mixed basis sets were carried out: (1) geometry optimizations and frequency calculations were performed with a set of basis set of Lanl2dz for Mn, Lanl2dzdp for I, 6-31G^*^ for S, 6-31G^**^ for the first coordinating moieties PhIO(I is excluded)/OH and 6-31G for the rest atoms. This basis set is denoted as B1 for simplicity. The benchmark on the other DFT functionals was added into the Supporting Materials. The validity of the functional B3LYP was evaluated by comparison with calculations that employed four other functionals: PBE0 (Adamo and Barone, 1999), B3PW91 (Kaupp et al., 2002), M06 (Zhao and Truhlar, 2008), and BP86 (Perdew, 1986), which are widely used in transition metal model systems. The hybrid DFT functionals (PBE0, B3PW91, M06) show consistent results with B3LYP (Supplementary Table S3). (2) Single point energy (SPE) calculations were done with the basis set of Lanl2tz for Mn, Lanl2dzdp (Hay and Wadt, 1985; Isaia et al., 2009; Jaccob et al., 2011) for I and 6-311+G^**^ for the rest atoms. Basis sets lanl2dzdp and lanl2tz were obtained from the Basis Set Exchange library (Feller, 1996; Schuchardt et al., 2007). All geometries were optimized without any symmetry constraints. Solvent effects (acetonitrile, ε = 35.688) were included in all calculations using the conductor-like polarizable continuum model (CPCM) (Barone and Cossi, 1998; Cossi et al., 2003) as implemented in Gaussian 16. An experimental temperature of 293.15 K was adopted in the Gibbs free energy calculations. Transition states were ascertained by vibrational frequency analysis to possess a mode along the reaction coordinate with a sole imaginary frequency. The energy values in the text were calculated at the SPE/B2//B1+ZPE(B1) level. Energy values at other computing levels are presented in the Supporting Materials.

## Result And Discussion

### Conversion of the Manganese(III)–Iodosylarene Complex 1 to the High-Valent Manganese(V)-oxo Complex 2

**Figure 1:**
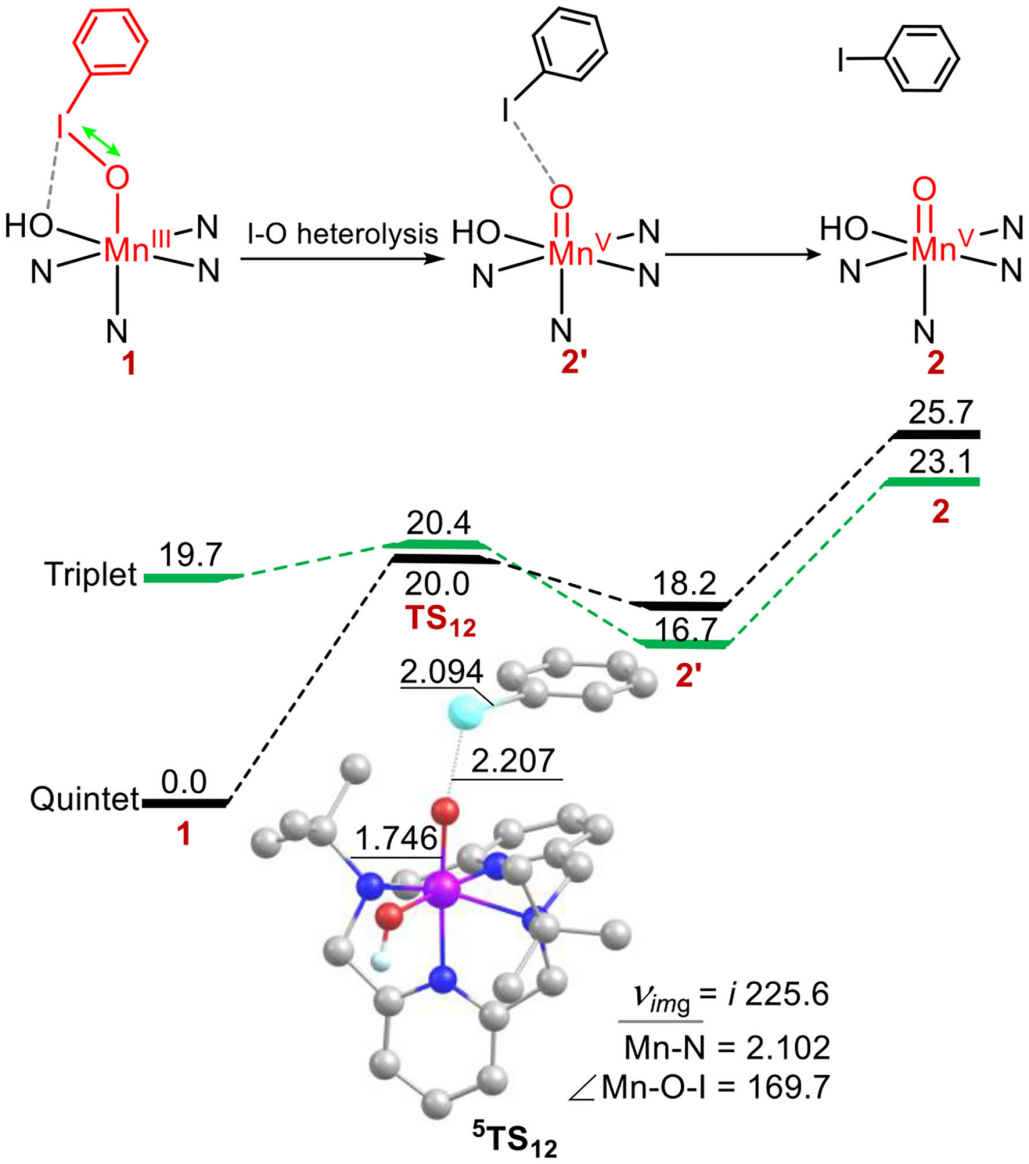
Energy profiles (in kcal mol^−1^) for the conversion of manganese(III)–iodosylbenzene 1 to oxo-manganese(V) 2. Energies were calculated at the UB3LYP-D3(BJ)/B2//B1+ZPE/B1 level in solvent. The geometric information of the transition state ^5^TS_12_ is presented. Hydrogen atoms are omitted for clarity. Lengths are in Å units, angles are in degree units, and the imaginary frequency is in cm^−1^ unit.

First, the conversion from **1** to high-valent metal-oxo species **2** in the absence of substrates was investigated (Figure 1). For **1**, the ground state is a high-spin quintet states (*S* = 2), the exited triplet/singlet spin states lie at 19.7/46.9 kcal/mol higher. Various SCF and free energies of the reactant on singlet state (*S* = 0) was found to have high energy throughout the reaction, Thus, the singlet state pathway was ruled out. For the ground state ^5^**1**, the average distance of Mn-N is 2.202 Å, the distance of Mn-OIPh is 1.904 Å, the I-O distance is 1.938 Å, and the length of twisted T-shape halogen bond I-OH is 2.689 Å, which is consistent with Cho's experiment (Jeong et al., 2018). For the transition states ^3, 5^**TS**_12_, the energies is degenerate. ^5^**TS**_12_ lies 20.0 kcal mol^−1^ higher than ^5^**1**, and ^3^**TS**_12_ only lies 0.4 kcal mol^−1^ above ^5^**TS**_12_. For the low-lying ^5^**TS**_12_, the calculated Mn-O distance is 1.746 Å, the I-O one is 2.207 Å, and the I-OH one is 4.592 Å (For ^5^**1**, the length is 2.689 Å), indicating that the halogen bond between I and OH for ^5^**1** is broken on the transition state and thus raises the activation energy (Liu et al., 2016). The formed high-valent Mn(V)-oxo complex on the triplet ground state ^3^**2**′ lies 16.7 kcal mol^−1^ higher than the quintet manages(III)-iodosylarene complex ^5^**1**. For ^3^**2**′, the Mn–O distance is 2.642 Å, indicating that I-O bond is still not completely broken and has a strong interaction. Such high-valent MO–iodine interaction was also found in the conversion of the iron(III)-PhIO complex Fe(III)(O)(tpena)-OIPh to the high-valent iron-oxo species Fe(V)(O)(tpena) (Lennartson and Mckenzie, 2012). To obtain a non-PhI-interacting Mn(V)(O)(TBDAP) complex **2** from **2**′, an additional energy of 6.4 kcal mol is required (Figure 1). In short, conversion of the quintet complex (TBDAP)Mn(III)-OIPh to the high-valent species Mn(V)(O)(TBDAP) is a process of two-state reactivity (TSR) (Shaik et al., 1998, 2002; Ogliaro et al., 2000). The halogen bond between the iodine atom and the OH ligand is broken during the conversion, making such conversion kinetically and thermodynamically unfeasible.

### Sulfoxidation of Thioanisole by 1 via the Direct Oxygen-Atom Transfer (DOT) Mechanism

**Figure 2:**
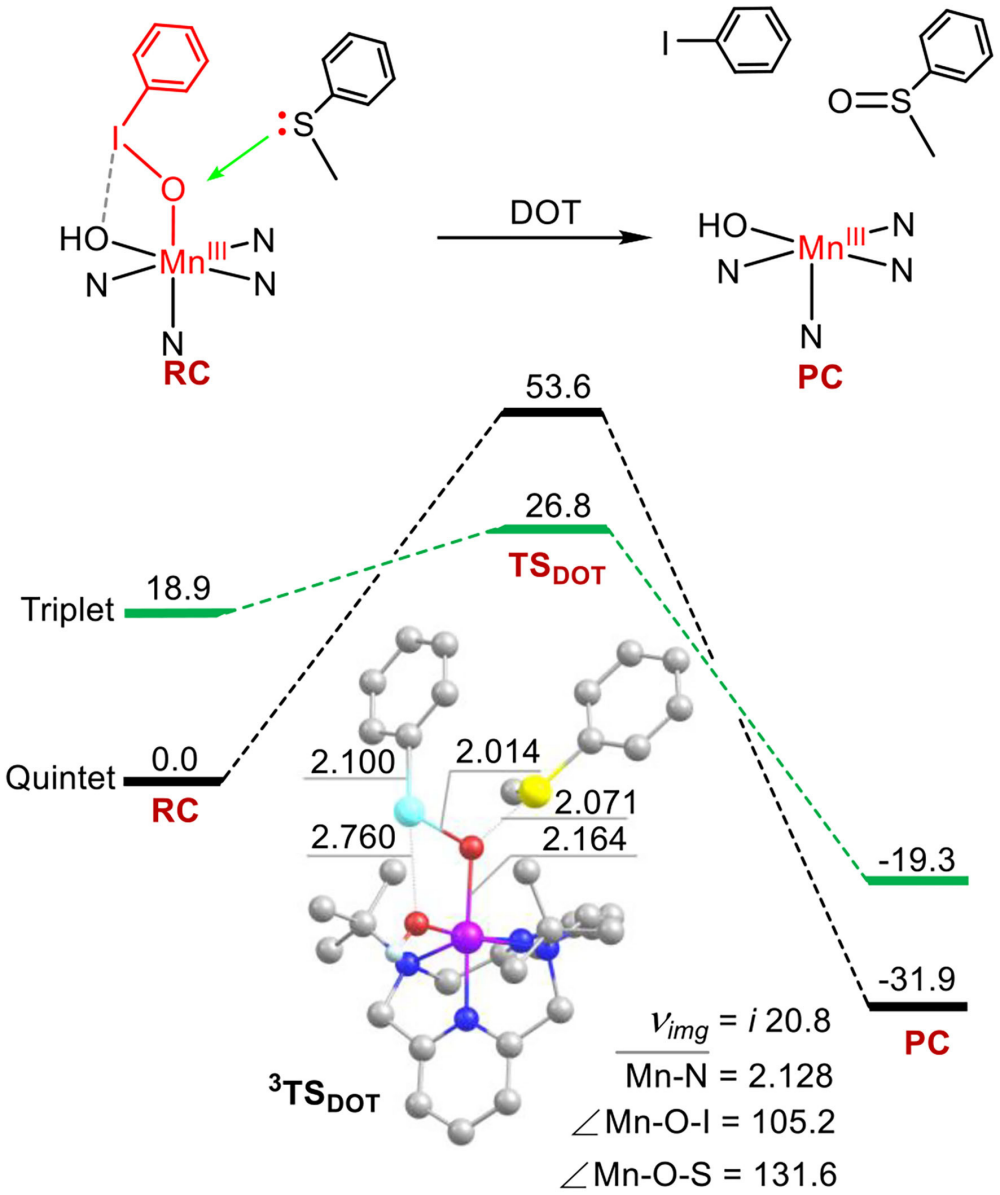
Energy profiles (in kcal mol^−1^) for thioanisole sulfoxidation via the direct oxygen-atom transfer mechanism. Energies were calculated at the UB3LYP-D3(BJ)/B2//B1+ZPE/B1 level in solvent. Key geometric information on transition states is presented. Hydrogen atoms are omitted for clarity. Energies are in kcal mol^−1^ units, lengths are in Å units, angles are in degree units, and imaginary frequencies are in cm^−1^ units.

Next, we investigated the structure-reactivity relationship of **1**. For sulfoxidation reaction of thioanisole by **1**, the oxidation via the DOT mechanism was firstly calculated. As shown in Figure 2, for the reactant complex (RC), the ground state is a high-spin quintet states (*S* = 2), as the elongation of I-O bond, spin reversion occurs and the reaction path switches from the quintet to the triplet spin state (*S* = 1) on the transition state. The formed product complex is on the quintet ground state. This reaction is a TSR process (Shaik et al., 1998, 2002; Ogliaro et al., 2000). For ^5^**RC**, the average distance of Mn-N is 2.172 Å, the length of Mn-OIPh is 1.951 Å, and the I-O one is 1.922 Å. For the transition states ^3, 5^**TS**_DOT_, ^5^**TS**_DOT_ lies 53.6 kcal mol^−1^ above by ^5^**RC** and ^3^**TS**_DOT_ lies 26.8 kcal mol^−1^. The activation energy barrier of the DOT process is very high. For the low-lying ^3^**TS**_DOT_, the Mn-O distance is 2.164 Å, the S-O one is 2.071 Å, the O-I one is 2.014 Å, and the length of between I and OH is 2.760 Å, which means I-O bond and halogen bond I-OH cannot completely broken and has a strong interaction. The elongation of the two bonds needs such high activation energy barrier (26.8 kcal/mol), indicating that it is very difficult to oxide the thioanisole by the DOT mechanism with the way of two-electron transfer for complex **1**. In previous work, Oae and his coworkers suggested ETOT mechanism for the sulfoxidation of a series of aromatic sulfides promoted by an iron (III) porphyrin (Oae et al., 1982). Lanzalunga also illustrate the aryl diphenylmethyl sulfides promoted by the non-heme iron(IV)-oxo complex occurs by electron transfer followed by oxygen transfer (ETOT) mechanism (Barbieri et al., 2016). Whether the sulfoxidation of thioanisole mediated by Mn(III)-iodosylarene complex was in the ETOT way?

### Sulfoxidation of Thioanisole by 1 via the Electron Transfer/Oxygen Transfer (ETOT) Mechanism

**Figure 3:**
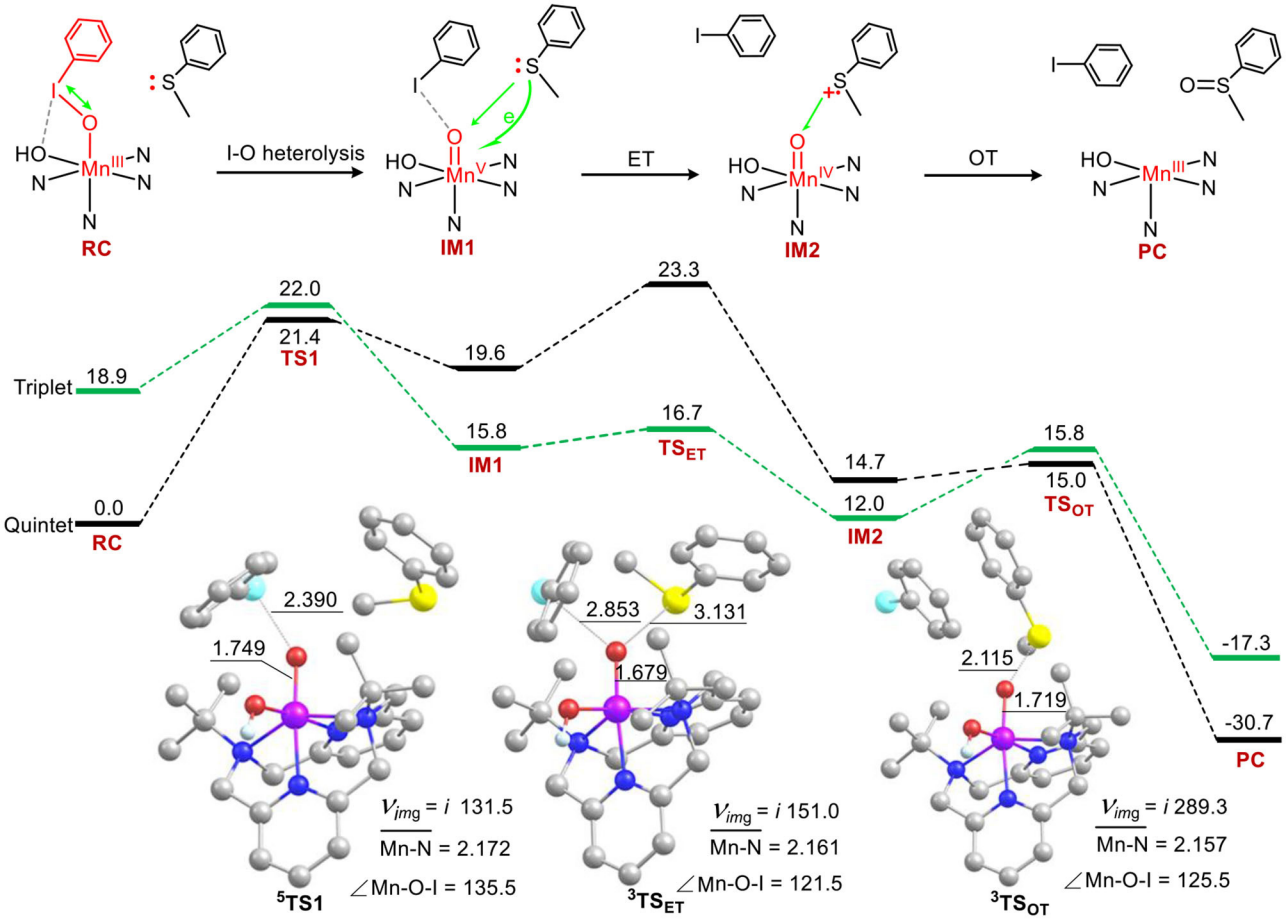
Energy profiles (in kcal mol^−1^) for the thioanisole sulfoxidation via the ETOT mechanism. Energies were calculated at the UB3LYP-D3(BJ)/B2//B1+ZPE/B1 level in solvent. The key geometric data of the transition states are presented. Hydrogen atoms are omitted for clarity. Lengths are in Å units, angles are in degree units, and imaginary frequencies are in cm^−1^ unit.

The calculated energy profiles for sulfoxidation via the ETOT mechanism have been presented in Figure 3. The activation barrier of the rate-limiting step of I-O bond cleavage is 21.4 kcal mol^−1^, which is 5.4 kcal mol^−1^ lower than the barrier of sulfoxidation in the DOT mechanism (Figure 2). For ^5^**TS**1, the I-O distance is 2.390 Å, the Mn-O distance is 1.749 Å, the S-O one was kept at 4.384 Å. The angle of Mn-O-I is 135.5°. Both the activation energy and the geometries of transitions states have introduced into the reaction system (comparing to Figure 1 for the case of absence of substrates). As the I-O bond further elongation, spin reversion occurs and the reaction path switches from the quintet state to the triplet one (*S* = 1) on the transition state 2. Thus, this is an another two-state reactivity (TSR) process (Shaik et al., 1998, 2002; Ogliaro et al., 2000). During the substrate thioanisole approaching, an intermolecular electron transfer occurs from thioanisole to the manganese-oxo moiety to form an Mn(IV)–oxo species with an one-electron oxidized thioanisole cation radical. For the low-lying ^3^**TS**_ET_, as depicted in Figure 3, the I-O distance is 2.853 Å, the Mn-O one is 1.679 Å, and the I-OH one is 3.460 Å, the angle of Mn-O-I is 121.5°. The spin of thioanisole is ca. 1.3. This conversion has a tiny barrier of 0.9 kcal mol^−1^. The following oxygen transfer step is a two-state reactivity process, with degenerate quintet and triplet transition states ^3, 5^**TS**_OT_ (^5^**TS**_OT_ is 0.8 kcal mol^−1^ lower than ^3^**TS**_OT_). The ground state of product complex is the quintet state and the products are the quintet Mn^III^ (TBDAP)(OH)^**−**^ complex and sulfoxide. This step is an exothermic process with large heat (42.7 kcal mol^−1^). In short, the reaction proceeds in a stepwise way; first involving a substrate-induced O-I bond small but obvious change when the substrate thioanisole is cleavage, following by an intermolecular electron transfer from the substrate to the Mn(V)–oxo core, and ended by an oxygen rebound step to form the sulfoxide product. In such way, the ETOT pathway has lower activation energy and becomes dominant over the DOT pathway.

### C-H Bond Activation of 9, 10-Dihydroanthracene by 1

**Figure 4:**
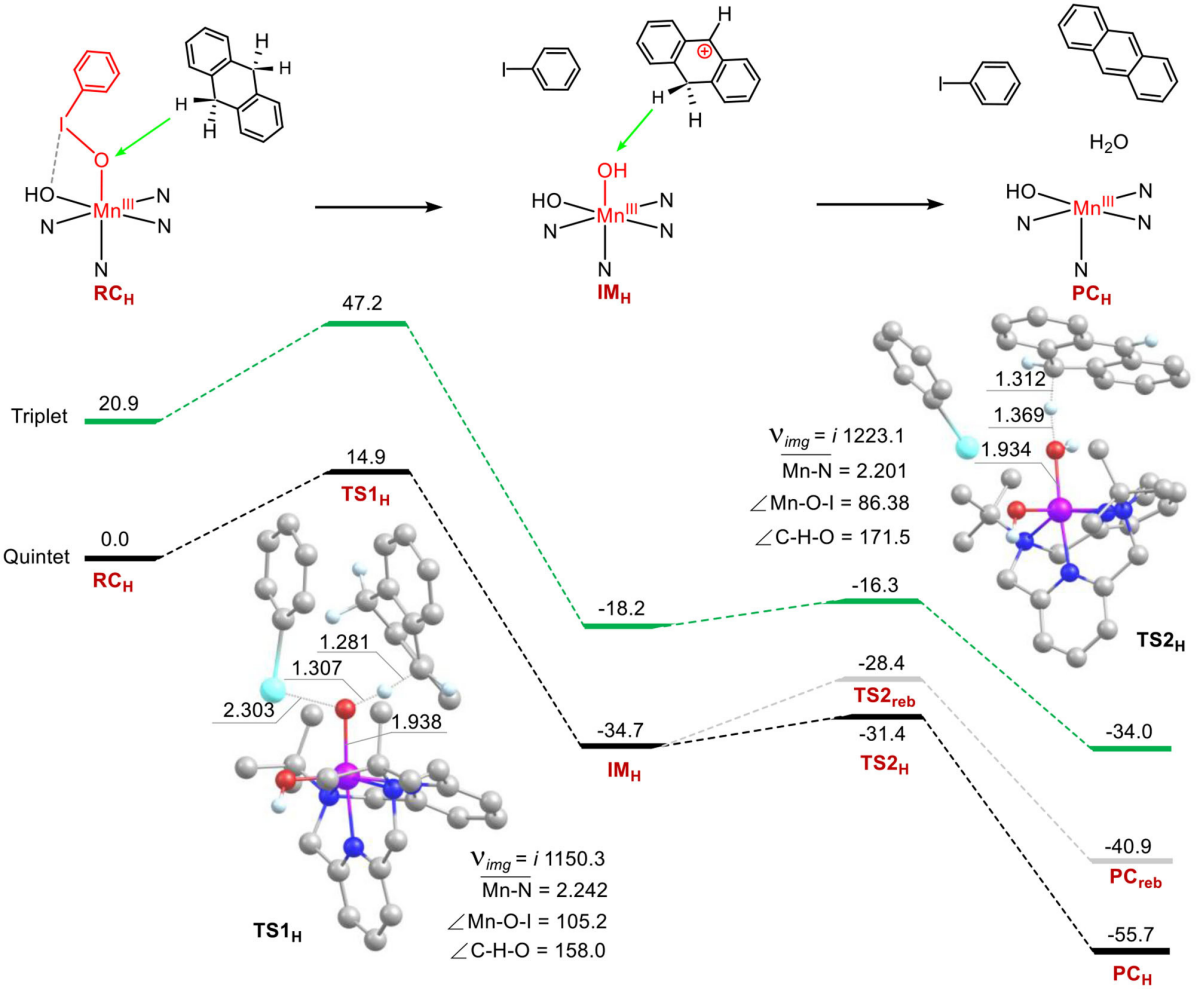
Energy profiles (in kcal mol^−1^) for the C-H bond activation of 9,10-dihydroanthracene. Energies were calculated at the UB3LYP-D3(BJ)/B2//B1+ZPE/B1 level in solvent. The key geometric information on the transition state TS1 and TS2 is presented. Unimportant hydrogen atoms are omitted for clarity. Lengths are in Å units, angles are in degree units, and imaginary frequencies are in cm^−1^ unit.

The C–H bond activation of hydrocarbons by metal-oxo active oxygen species is one of the most important subjects in bioinorganic and oxidation chemistry (Solomon et al., 2000; Nam, 2007; Shaik et al., 2007; Van Eldik, 2007; Gunay and Theopold, 2010; Mayer, 2010; Cho et al., 2012). The reactivity of **1** in the C–H activation reaction was investigated as well. Energy profiles for the C-H bond activation of 9, 10-dihydroanthracene (DHA) have been present in Figure 4. Surprisingly, the manganese-iodosylarene complex **1** exhibits robust oxidative ability toward DHA. The rate-limiting step of the first C-H abstraction step holds a barrier of 14.9 kcal mol^−1^, which is much lower than the barriers in sulfoxidation of thioanisole. The result is also in agreement with the rank of second-order rate constant (Jeong et al., 2018). For ^5^**TS**1_H_, the Mn-O distance is 1.938 Å, the I-O one is 2.303 Å, the Mn-O-I angle is 105.2°. For the reaction coordinates, the C-H distance is 1.281 Å, the O-H distance is 1.307 Å and the C-H-O angle is 158.0°. The lengths of the O-H moiety and the C-H one are consistent with normal C-H abstraction protocol, while the C-H-O angle is too bent (normally the angle is nearly 180° for C-H abstraction by high-valent metal-oxo species). Investigation of ^5^TS1_H_'s geometry, we can see there is a strong stacking interaction between the phenyl ring of PhI and the ring of DHA. Such a reaction is beneficial to lower the activation energy. After the transition state, the H-abstracted DHA moiety (DHA-H) automatically rotates and directs the second C-H moiety of the methylene moiety (-CH_2_-) to the nascent Mn(III)-(OH)_2_ complex (**IM**_H_). Surprisingly, There is no spin for H and the DHA-H moiety at the intermediate state **IM**_H_ (Supplementary Table S10), the Mulliken charge of DHA-H changes from a negative value (ca. −0.16) in the reagent complex to a positive one (ca. 0.95) in the **IM**_H_. Thus, the DHA-H becomes a carbonium species, which could well explain the rotation of DHA as the repulsive effect between cationic carbonium and H parts. Thus, the first C-H bond activation step is a novel hydride transfer (HT) process. Subsequently, a second C-H abstraction of the methylene group by the Mn(III)-(OH)_2_ complex occurs. The barrier of the second hydrogen abstraction is only 3.3 kcal mol^−1^, it's an easy process to occur. For the low-lying ^5^**TS**2_H_, there is no spin populated in the DHA-H moiety yet, and the Mulliken charge of DHA-H is decreased (ca. 0.38), demonstrating that the second step is a proton transfer (PT) process. We also calculated the possible oxygen rebound step and found that the energy of the transition state for the rebound step is 3.0 kcal mol^−1^ higher than the energy of the second H-abstraction step. The exothermicity of the rebound step is also 14.8 kcal mol^−1^ less than that of the second hydrogen abstraction step. Thus, C-H activation of DHA by **1** proceeds via a novel ionic hydride transfer/proton transfer (HT/PT) mechanism, not the H-abstraction/O-rebound mechanism, or the dual hydrogen abstraction mechanism in P450 chemistry. Interestingly, for the transition state of the second hydrogen abstraction, the reaction coordinate C-H-O is nearly colinear (the C-H-O angle is 171°). This is for the sake that in ^5^**TS2**_H_, there is no stacking interaction. At the product complex state, a H_2_O and the manganese(III) catalyst are formed for the second catalytic cycle. In short, our calculations support the mechanistic proposed by the Cho's experiment that **1** is a good electrophilic agent in oxidative C-H bond activation (Jeong et al., 2018).

### The Electrophilicity of the Iodosylbenzene Monomer PhIO in Thioanisole Sulfoxidation and in C-H Bond Activation

**Figure 5:**
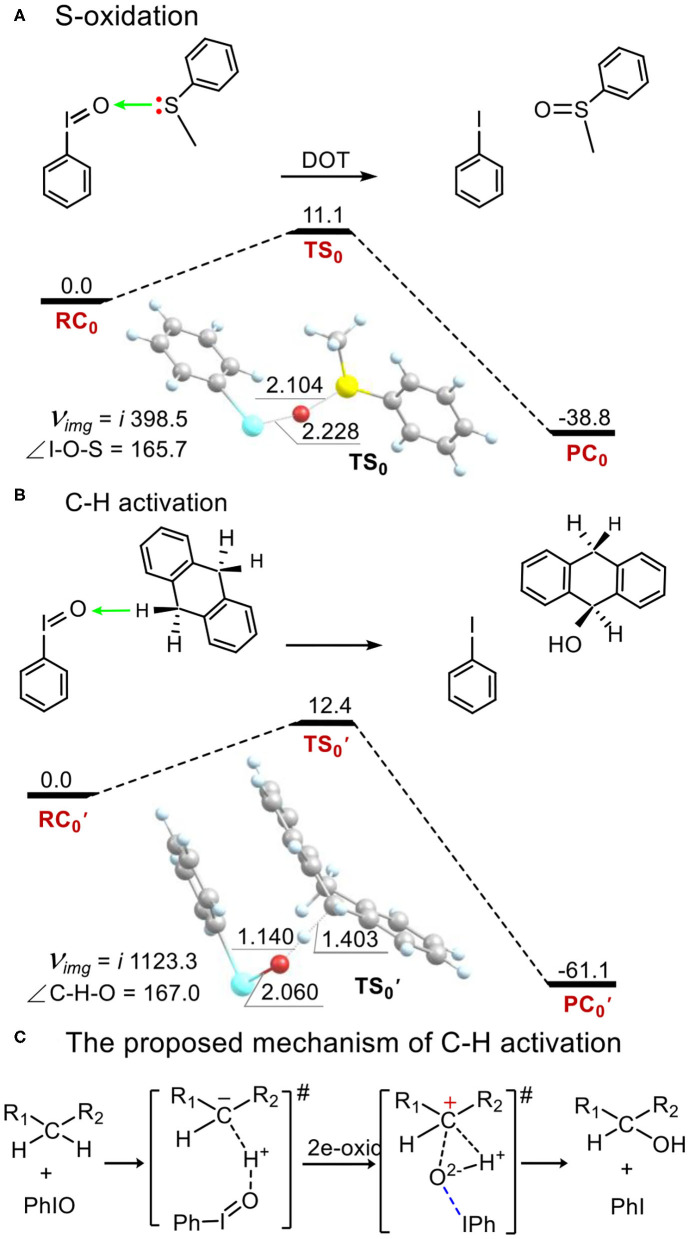
Energy profiles (in kcal mol^−1^) for **(A)** thioanisole sulfoxidation and **(B)** C-H bond activation of 9,10-dihydroanthracene by the PhIO monomer. **(C)** An ionic proton-transfer/hydroxyl-rebound mechanism was proposed for C-H activation by PhIO. Energies were calculated at the UB3LYP-D3(BJ)/B2//B1+ZPE/B1 level in solvent. Geometric data of transition states are presented. Lengths are in Å units, angles are in degree units, and imaginary frequencies are in cm^−1^ unit.

As a comparison to the electrophilicity of metal-iodosybenzene adduct **1**, we investigated the electrophilicity of an iodosylbenzene monomer PhIO in thioanisole sulfoxidation and in C-H bond activation of DHA. As shown in Figure 5, PhIO acts as a robust electrophilic agent in these two oxidations (Barbieri et al., 2016). In the activation energy is only 11.1 kcal mol^−1^ in thioanisole sulfoxidation (Figure 5A) and is only 12.4 kcal mol^−1^ in hydroxylation of DHA (Figure 5B), which is consistent with the calculated results of hydroxylation of ethylbenzene by PhIO (Kim et al., 2009; Kumar et al., 2009; Kang et al., 2017). In sulfoxidation, the mechanism is a direct oxygen transfer (DOT) mechanism. For the transition state **TS**_0_, the I-O distance is 2.228 Å, the S-O one is 2.104 Å and the angle of I-O-S is 165.7°. Mulliken spin of the relaying oxygen is zero. In hydroxylation of DHA, the reaction mechanism is not the stepwise radical-involving hydrogen-abstraction/oxygen-rebound mechanism shown in alkane hydroxylation by high-valent metal-oxo reaction intermediates. The reaction is concerted and for the sole transition state ${\text{TS}}_{0}^{{\;}^{\prime }}$ (Figure 5B), the O-H distance is 1.140 Å, the C-H one is 1.403 Å and the C-H-O angle is 167.0°, which is not as colinear as the one in alkane hydroxylation by high-valent metal-oxo. Such non-colinear hydrogen abstraction is also reported by de Visser and Nam (Kim et al., 2009). We can find that there is no spin in any moieties at the transition state ${\text{TS}}_{0}^{{\;}^{\prime }}$. Mulliken charge of H-abstracted DHA (DHA-H) changes from a negative value (ca. −0.3) in the reagent complex and the transition state to a positive one (ca. 0.3) after the transition state alongside the intrinsic reaction coordinate (Supplementary Figures S12, S13). Thus, hydroxylation by PhIO is an ionic and concerted proton-transfer/hydroxyl rebound process (Figure 5C).

## Conclusions

In the present manuscript, the structure-function relationship of a metal-iodosylarene adduct [Mn^III^(TBDAP)(OIPh)(OH)]^2+^ **1** in sulfoxidation and oxidative C-H bond activation were investigated by means of density functional theoretical calculation. The calculated results are consistent with the experimental results and the conclusion by Cho et al. that the metal-iodosylarene adduct **1** is a good electrophilic agent. The theoretical study also revealed some new interesting mechanistic insights into the electrophilicity of **1**. **1** behaves as a stepwise 1e+1e oxidant in sulfoxidations, oxygen transfer occurs via the electron transfer followed by oxygen transfer (ETOT). While In oxidation of DHA, *a novel and ionic hydride transfer/proton transfer (HT/PT) mechanism, not the normal hydrogen-abstraction/oxygen rebound mechanism or the dual hydrogen abstraction mechanism in P450 chemistry, was found to mediate the reaction*. Such new mechanistic properties are caused by the halogen bond between phenyl ring of PhIO and the ligated hydroxyl group, and also by the stacking interaction between the phenyl ring of PhIO and substrates. As a comparison to the electrophilicity of the metal-iodosylarene adduct **1**, the structure-function relationship of an iodosylbenzene monomer PhIO is also presented. The calculated results demonstrated that electrophilicity of PhIO is even more robust than that of **1**. PhIO behaves as a 2e-oxidant in both thioanisole oxidation and hydroxylation of DHA. *A new ionic and concerted proton transfer/hydroxyl rebound mechanism involving 2e-oxidation to form a transient carbonium species* was proposed for the hydroxylation of DHA by PhIO.

## Data Availability Statement

All datasets presented in this study are included in the article/Supplementary Material.

## Author Contributions

All authors listed have made a substantial, direct and intellectual contribution to the work, and approved it for publication.

## Conflict of Interest

The authors declare that the research was conducted in the absence of any commercial or financial relationships that could be construed as a potential conflict of interest.
