# Supplementary material for: Theoretical Study on the Structural-Function Relationship of Manganese(III)-Iodosylarene Adducts
Source: Front Chem. 2020 Aug 20;8:744. doi: 10.3389/fchem.2020.00744 (PMC7469263; doi:10.3389/fchem.2020.00744)
Supplement: Supplementary file 1 [file Data_Sheet_1.pdf]

Supplementary Material

Theoretical Study on the Structural-Function Relationship of Manganese(III)-iodosylarene Adducts

Dongru Sun[1]*, Xiaolu Chen[1], Lanping Gao[1], Yufen Zhao[1] and Yong Wang[1]*

[1]Institute of Drug Discovery Technology, School of Material Science and Chemical Engineering, Ningbo University, Ningbo 315211, China.

*** Correspondence:**
Dongru Sun
sundongru@nbu.edu.cn

Yong Wang
yong@nbu.edu.cn.

1 Supplementary Figures

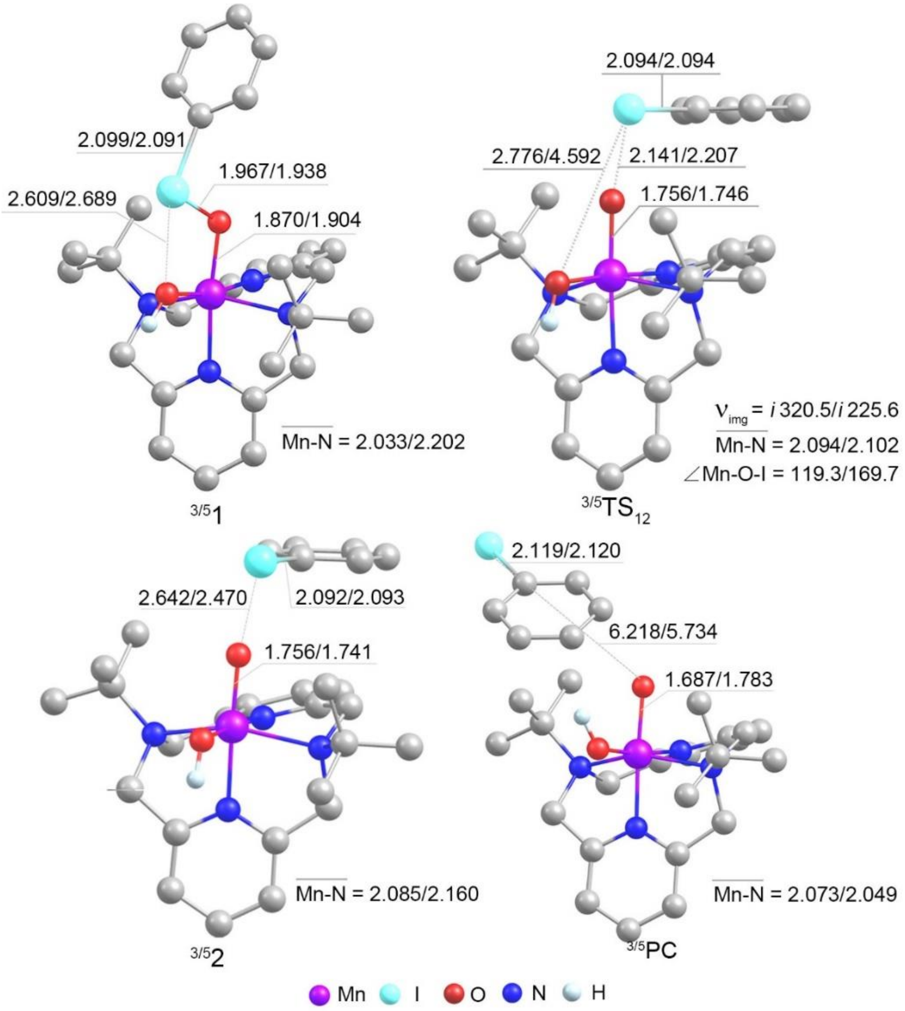

Supplementary Figure 1 The geometric information of reaction intermediates in the formation of high-valent manganese-oxo complex **2** from complex **1**. Length is in Å, bond angle is in degree and imaginary frequency is in cm^{-1} unit. Hydrogen atoms are omitted for clarity. Calculations were done at the UB3LYP-D3(BJ)/B1 level.

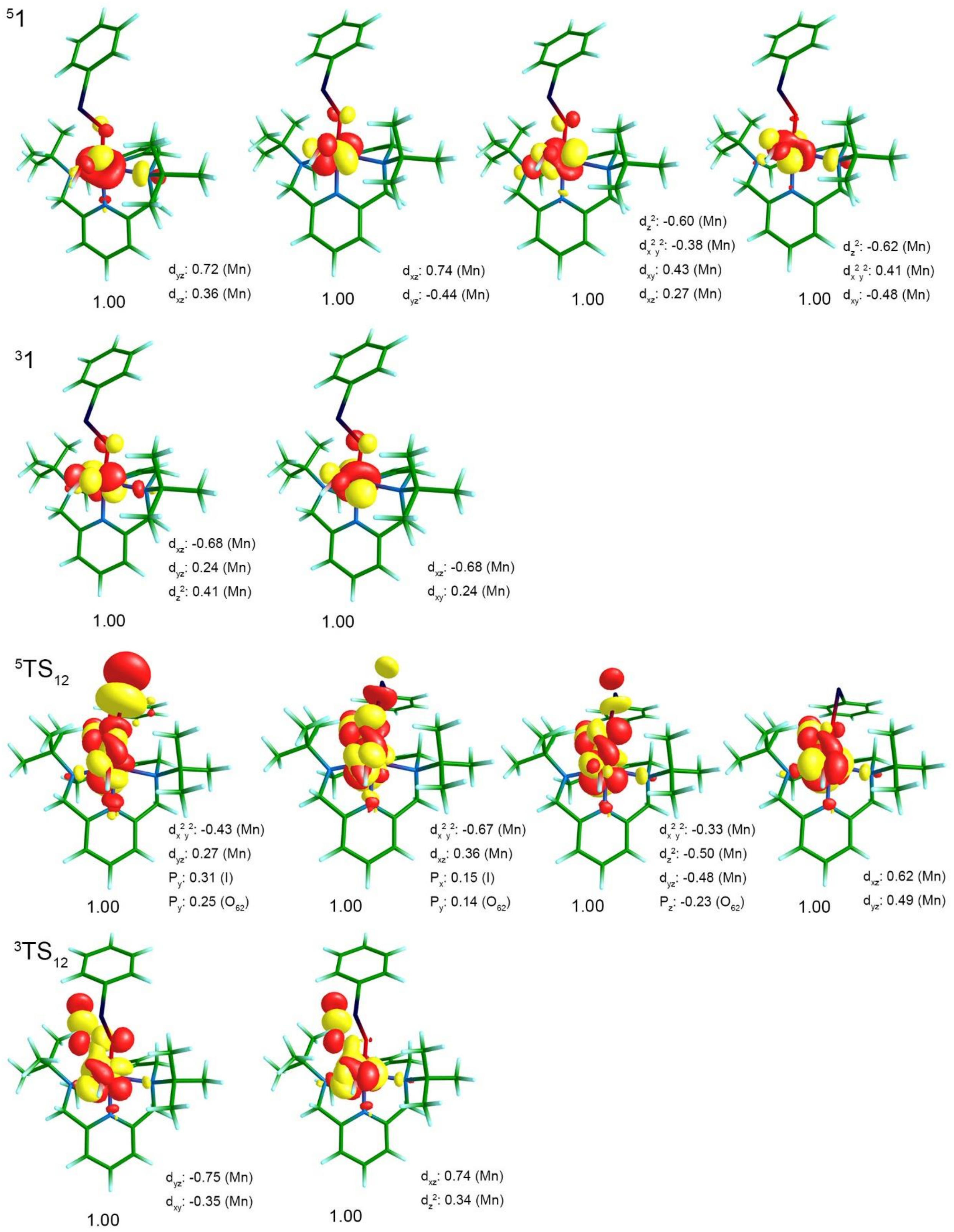

Supplementary Figure 2. Singly occupied spin natural orbitals (SNOs) of critical species along the formation of high-valent metal-oxo complex 2 from complex 1. Calculations were done at the UB3LYP-D3(BJ)/B1 level. Positive values represent alpha-electron occupations. (To be continued).

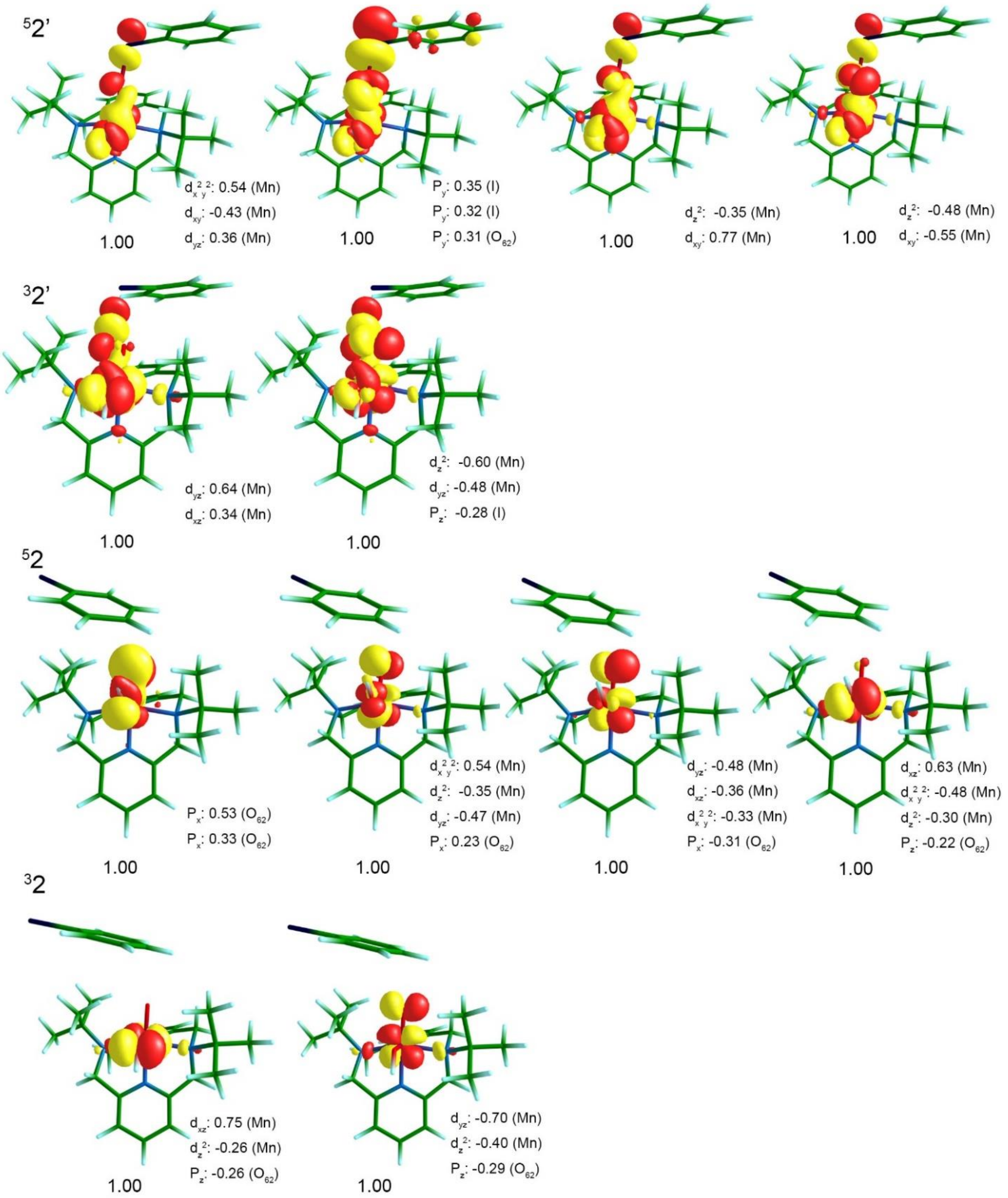

Supplementary Figure 2. Singly occupied spin natural orbitals (SNOs) of critical species along the formation of high-valent metal-oxo complex 2 from complex **1**. Calculations were done at the UB3LYP-D3(BJ)/B1 level. Positive values represent alpha-electron occupations.

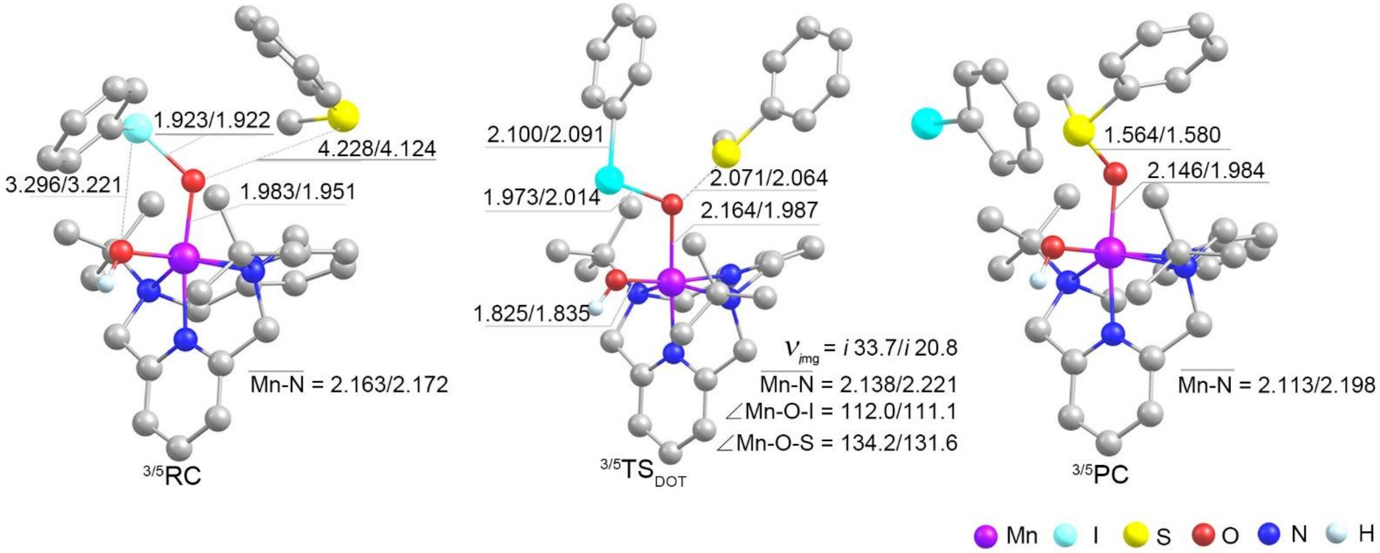

Supplementary Figure 3. The geometric information of the reaction intermediates in thioanisole sulfoxidation by complex **1** via direct oxygen-atom transfer mechanism. Length is in Å, bond angle is in degree and imaginary frequency is in cm^{-1} unit. Hydrogen atoms in the ligand are omitted for clarity. Calculations were done at the UB3LYP/B1 level.

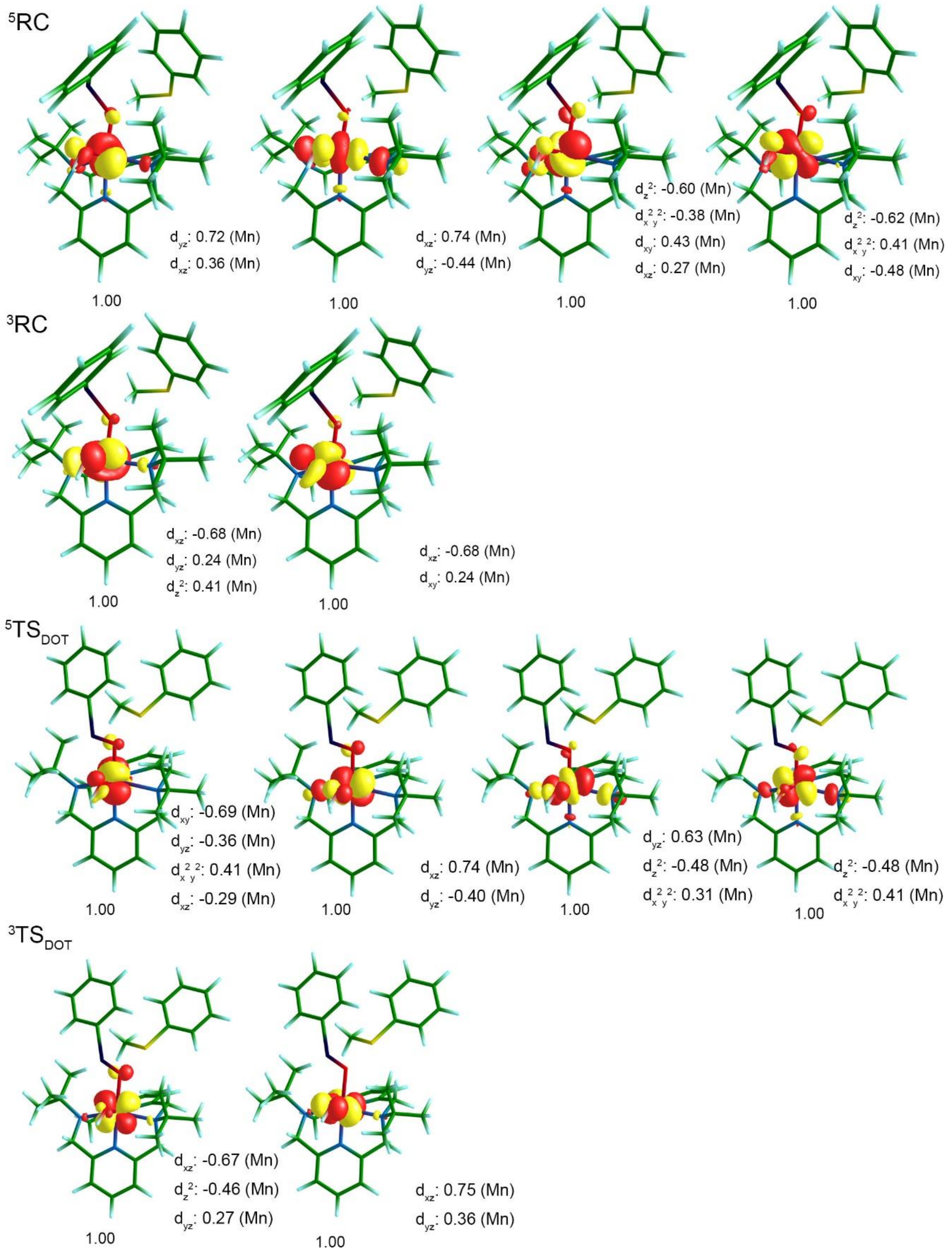

Supplementary Figure 4. Singly-occupied natural orbitals (SNOs) of the reaction intermediates in thioanisole sulfoxidation by complex **1** via the direct oxygen-atom transfer mechanism. Calculations were done at the UB3LYP/B1 level. Positive values represent alpha-electron occupations. (To be continued).

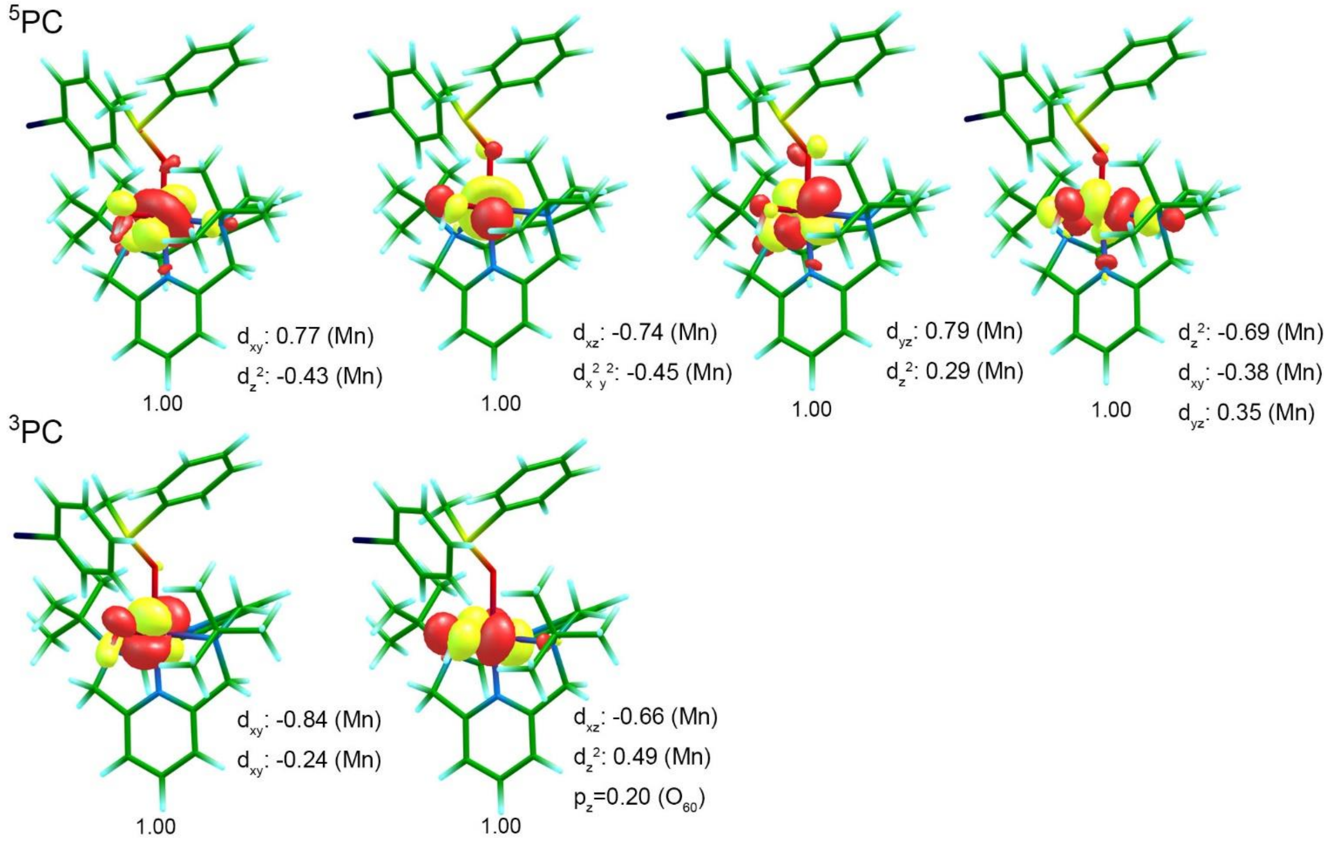

Supplementary Figure 4. Singly-occupied natural orbitals (SNOs) of the reaction intermediates in thioanisole sulfoxidation by complex **1** via the direct oxygen-atom transfer mechanism. Calculations were done at the UB3LYP/B1 level. Positive values represent alpha-electron occupations.

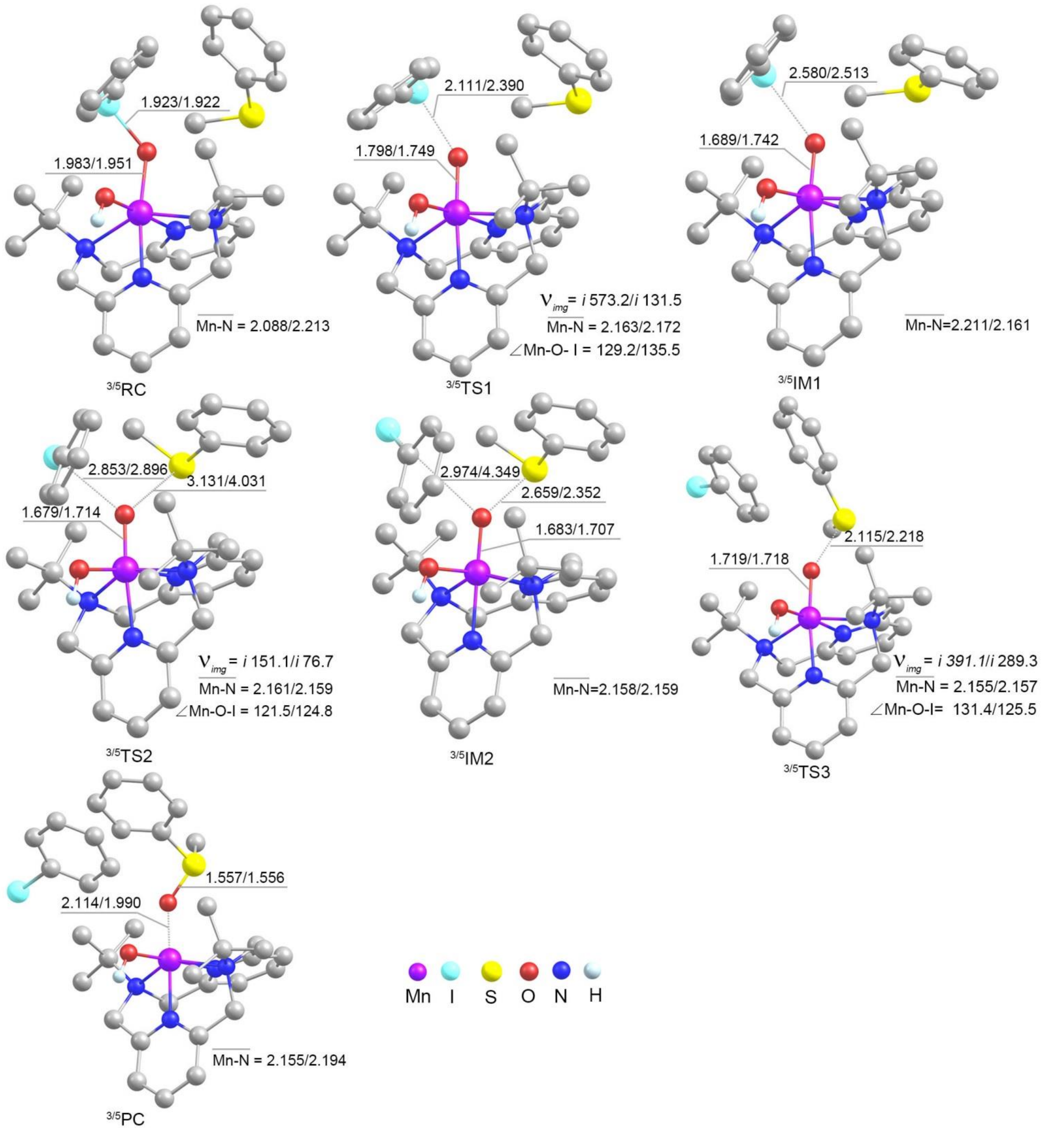

Supplementary Figure 5. The geometric information of the reaction intermediates in thioanisole sulfoxidation by complex **1** via the electron transfer/oxygen transfer mechanism. Length is in Å, bond angle is in degree and imaginary frequency is in cm^{-1} unit. Hydrogen atoms in the ligand are omitted for clarity. Calculations were done at the UB3LYP/B1 level.

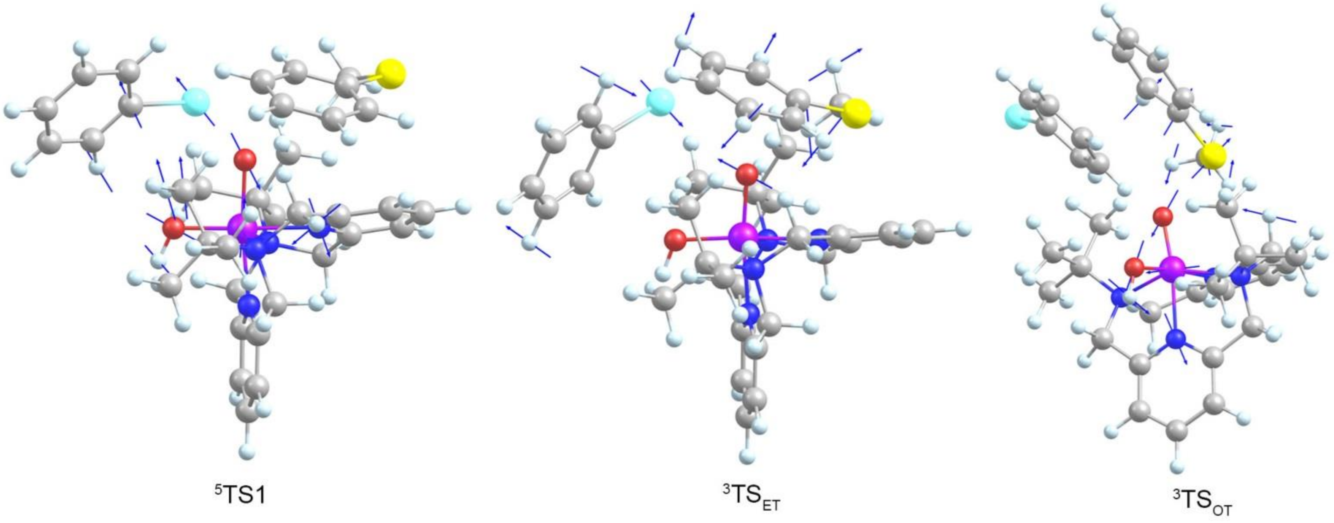

Supplementary Figure 6. The structures of all transition states (TSs) in the sulfoxidation reactions by complex **1** via the electron transfer/oxygen transfer mechanism.. The arrows indicate the vibrational directions of the imaginary frequency of transition states.

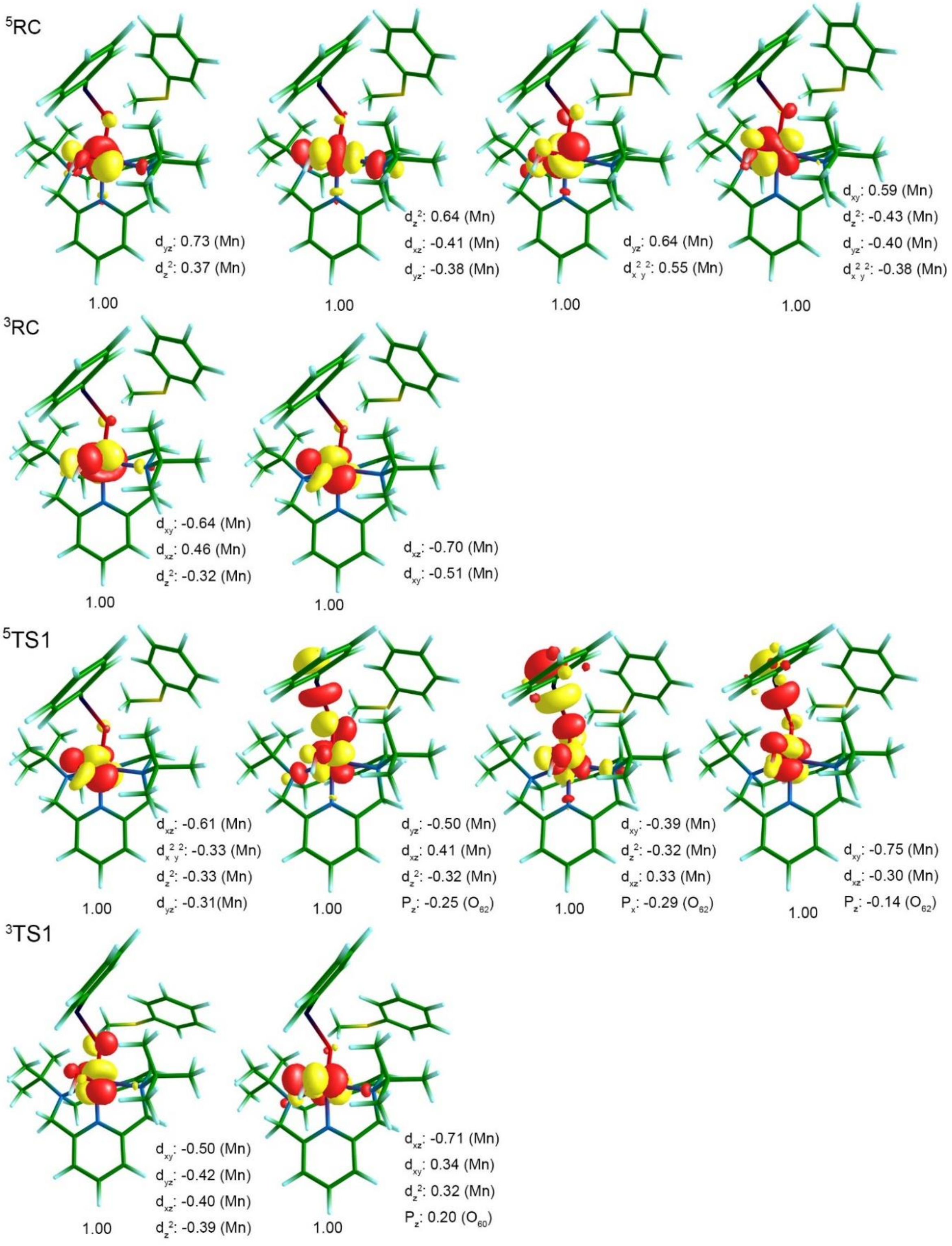

Supplementary Figure 7. Singly-occupied natural orbitals (SNOs) of the reaction intermediates in thioanisole sulfoxidation by **1** via electron transfer/oxygen transfer mechanism mechanism. Calculations were done at the UB3LYP/B1 level. Positive values represent alpha-electron occupations. (To be continued).

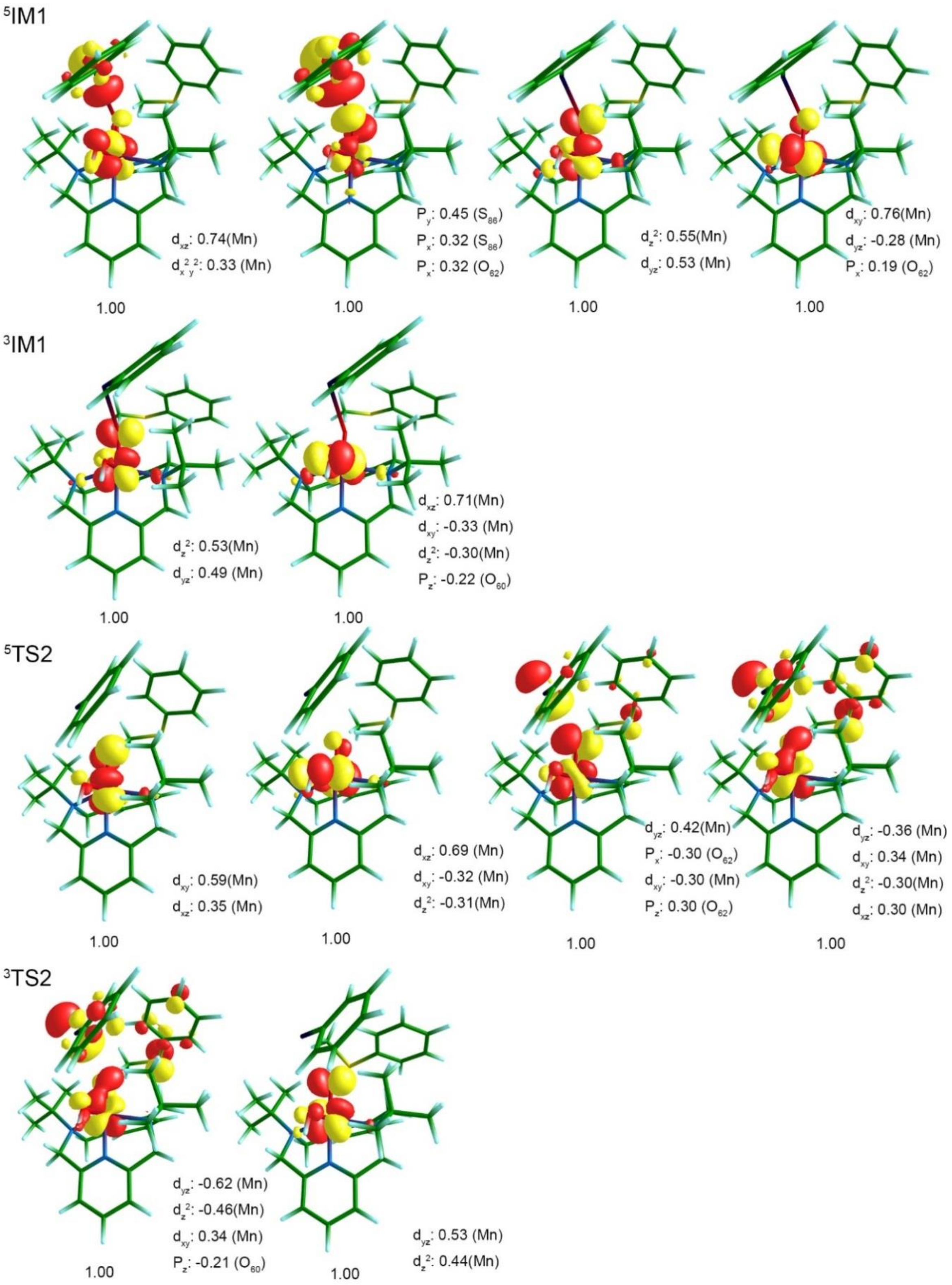

Supplementary Figure 7. Singly-occupied natural orbitals (SNOs) of the reaction intermediates in thioanisole sulfoxidation by **1** via electron transfer/oxygen transfer mechanism. Calculations were done at the UB3LYP/B1 level. Positive values represent alpha-electron occupations. (To be continued).

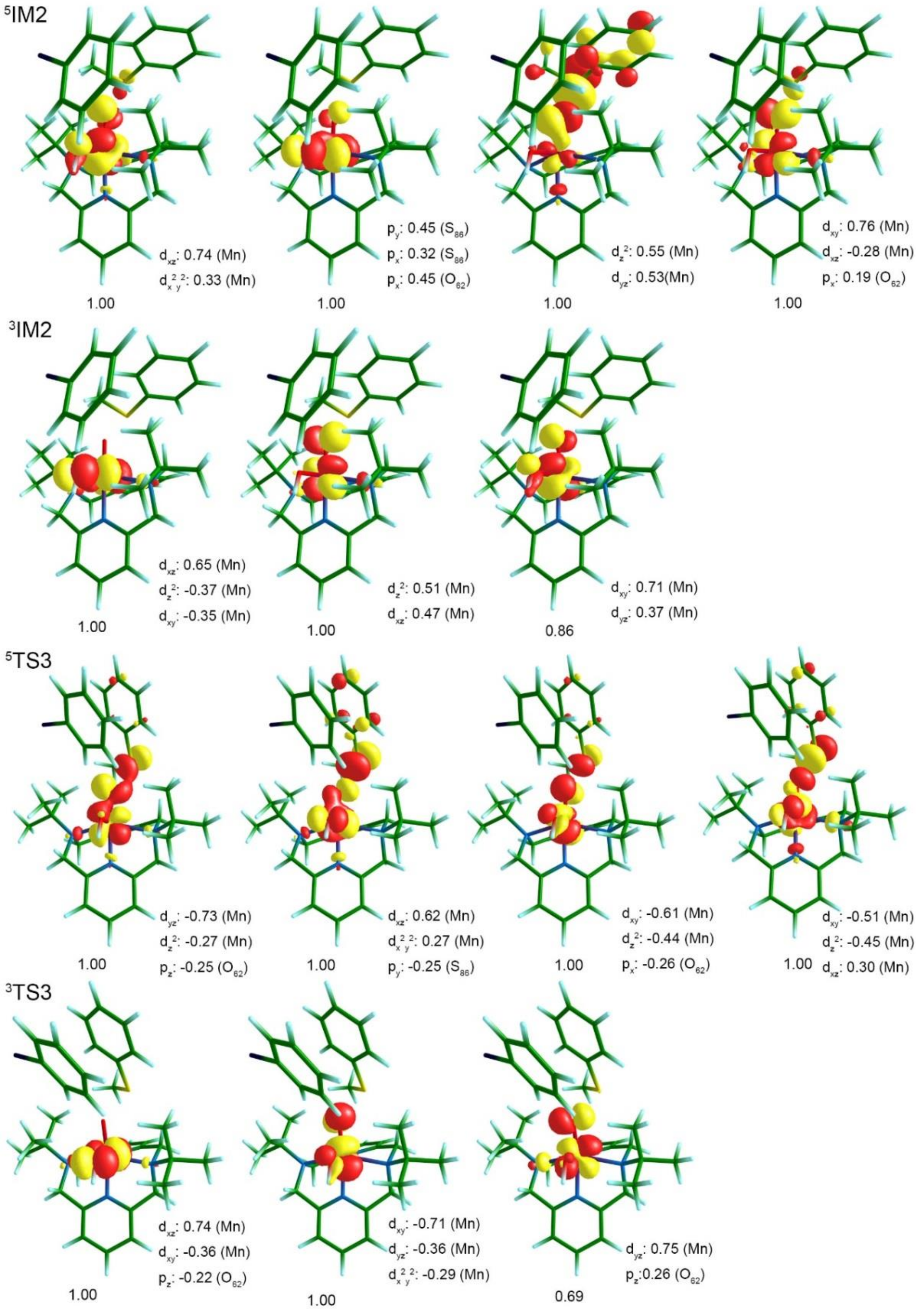

Supplementary Figure 7. Singly-occupied natural orbitals (SNOs) of the reaction intermediates in thioanisole sulfoxidation by **1** via electron transfer/oxygen transfer mechanism. Calculations were done at the UB3LYP/B1 level. Positive values represent alpha-electron occupations. (To be continued).

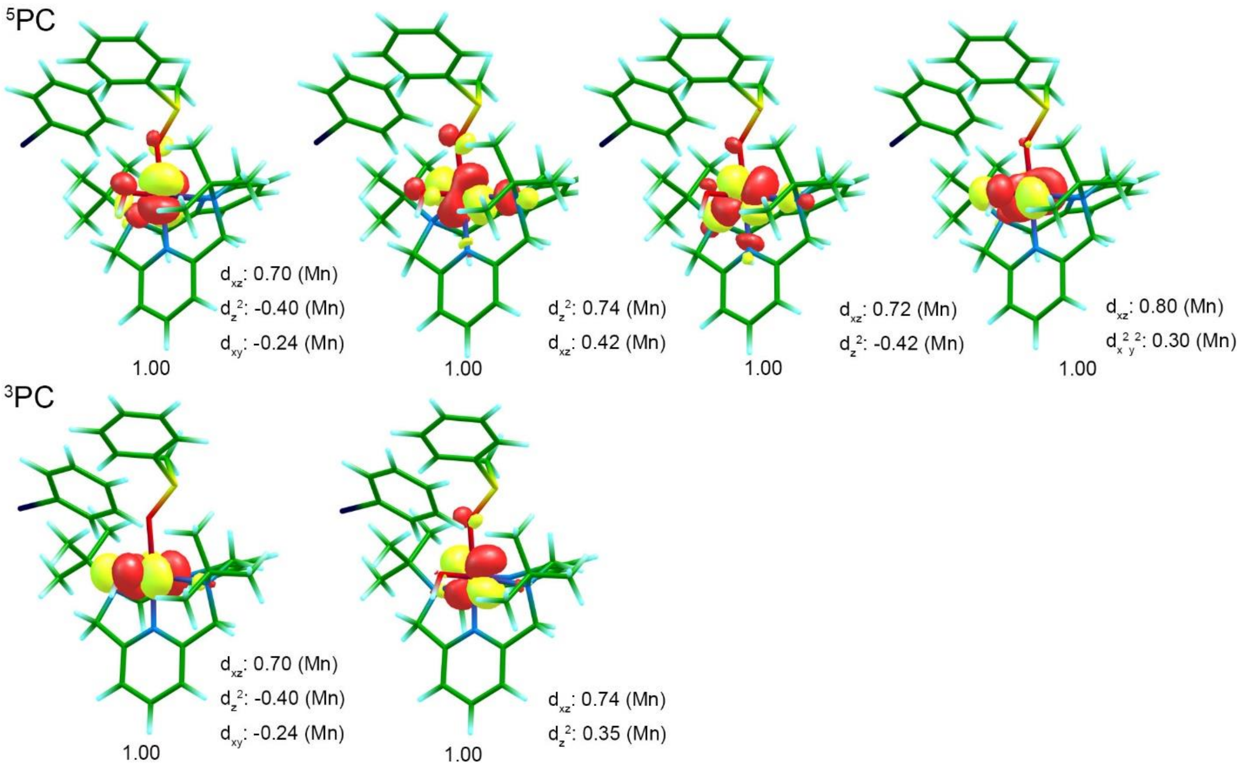

Supplementary Figure 7. Singly-occupied natural orbitals (SNOs) of the reaction intermediates in thioanisole sulfoxidation by **1** via electron transfer/oxygen transfer mechanism. Calculations were done at the UB3LYP/B1 level. Positive values represent alpha-electron occupations.

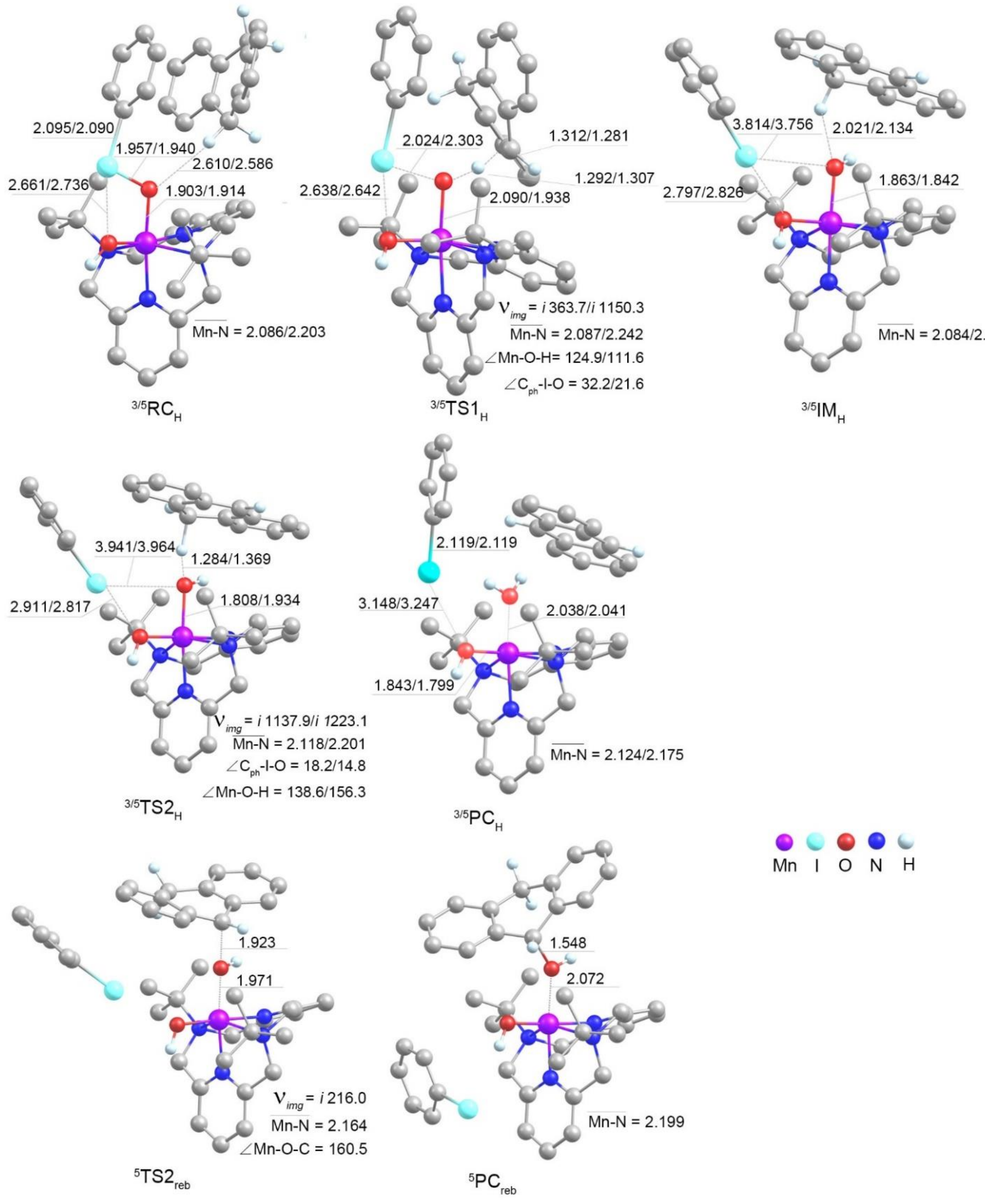

Supplementary Figure 8. The geometric information of different spin states of the reaction intermediates in the C-H bond activation of 9,10-dihydroanthracene mediated by complex **1**.Length is in Å, bond angle is in degree and imaginary frequency is in cm^{-1} unit. Hydrogen atoms in the ligand are omitted for clarity. Calculations were done at the UB3LYP/B1 level.

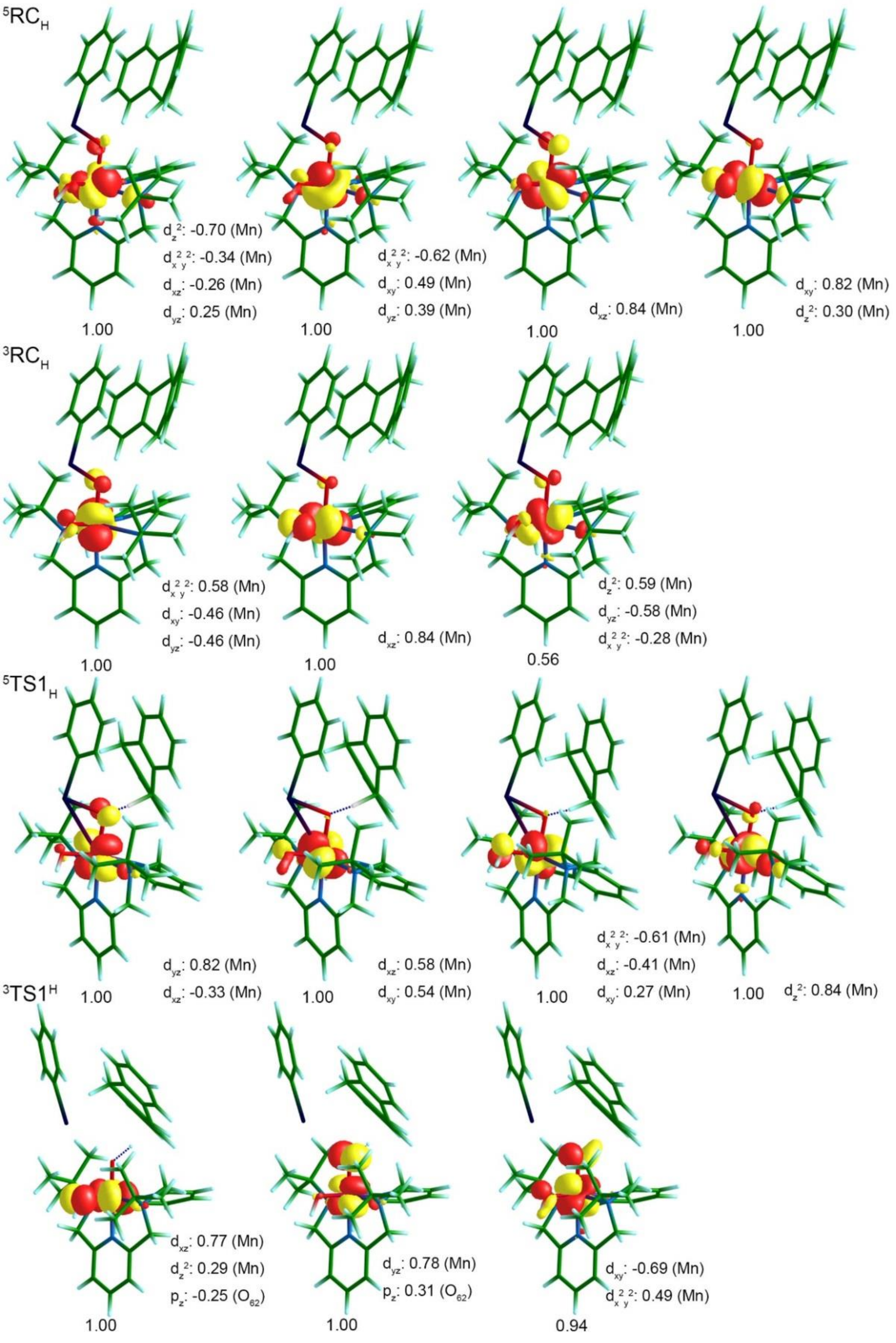

Supplementary Figure 9. Singly occupied spin natural orbitals (SNOs) of the reaction intermediates in C-H bond activation of 9,10-dihydroanthracene mediated by complex **1**. Calculations were done at the UB3LYP/B1 level. Positive values represent alpha-electron occupations. (To be continued).

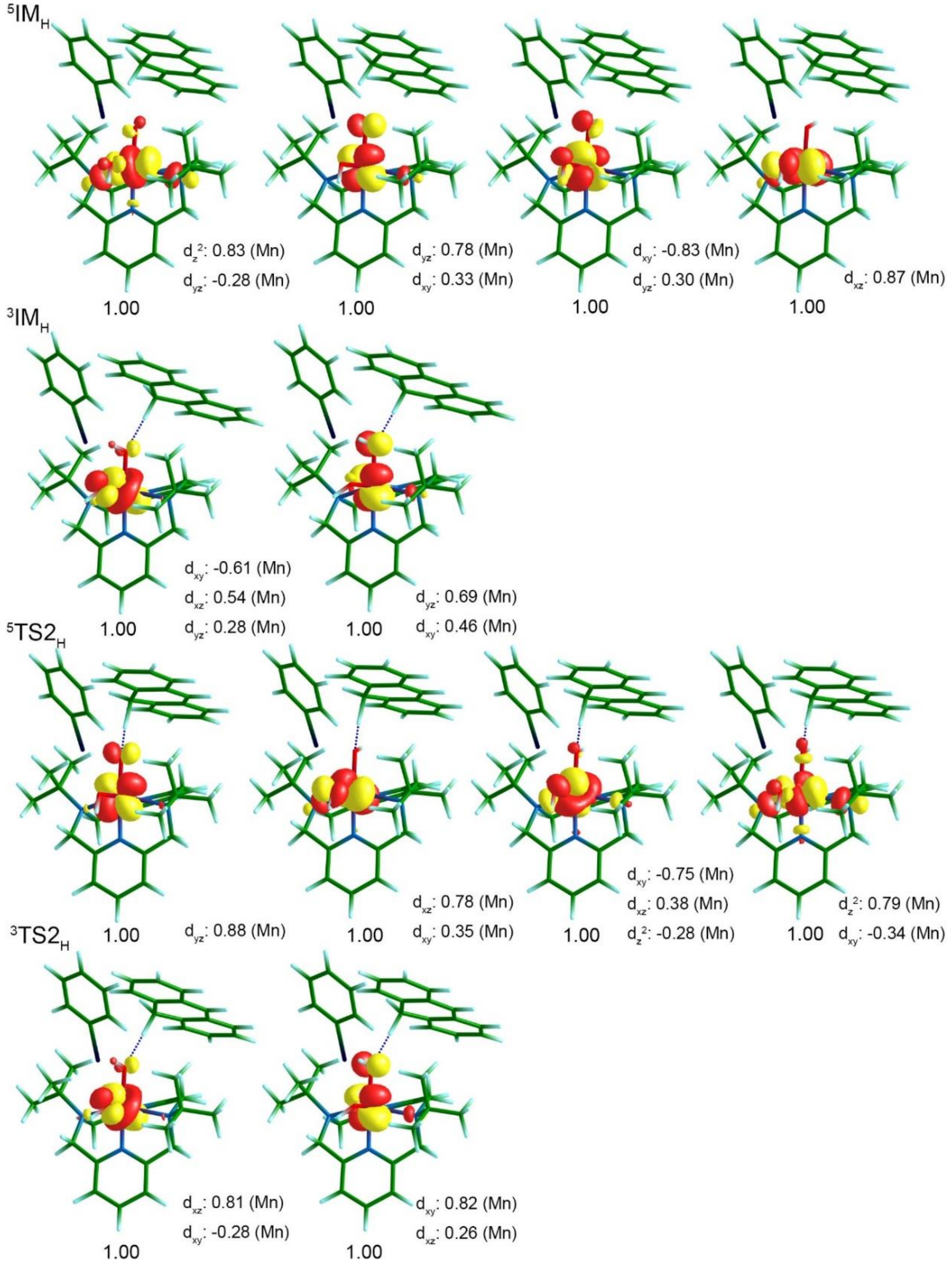

Supplementary Figure 9. Singly occupied spin natural orbitals (SNOs) of the reaction intermediates in C-H bond activation of 9,10-dihydroanthracene mediated by complex **1**. Calculations were done at the UB3LYP/B1 level. Positive values represent alpha-electron occupations. (To be continued).

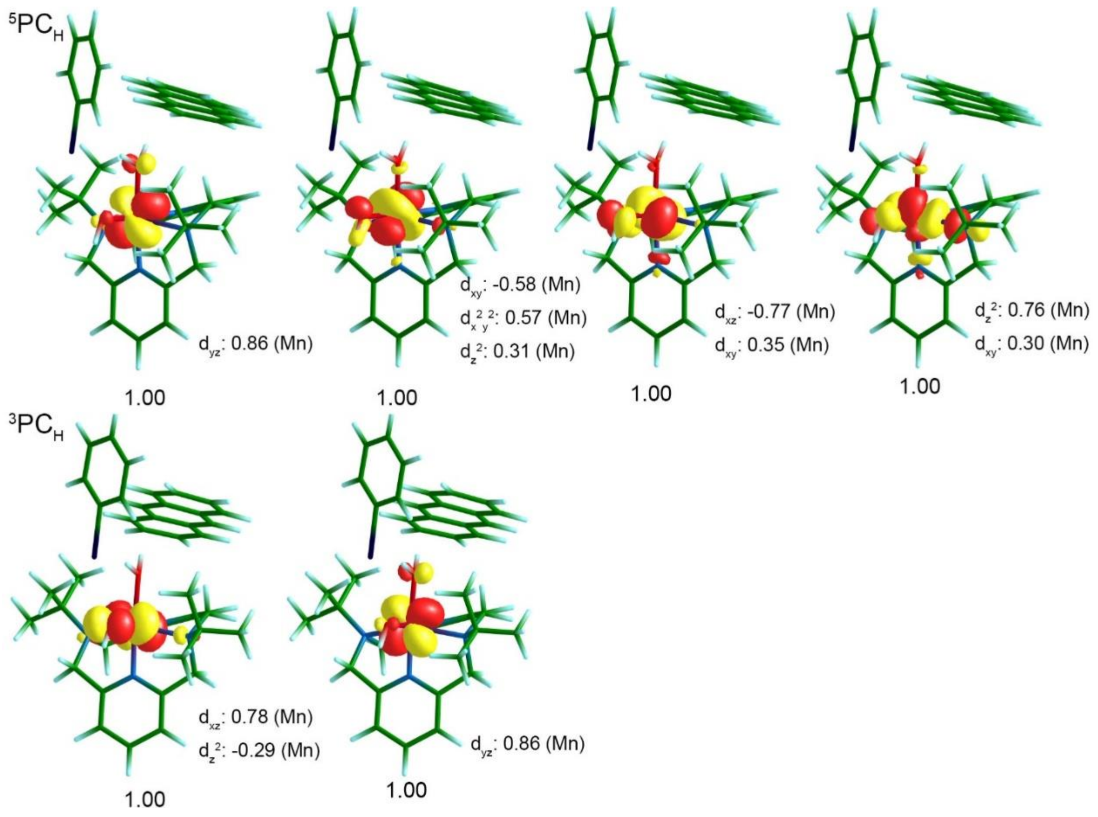

Supplementary Figure 9. Singly occupied spin natural orbitals (SNOs) of the reaction intermediates in C-H bond activation of 9,10-dihydroanthracene mediated by complex **1**. Calculations were done at the UB3LYP/B1 level. Positive values represent alpha-electron occupations.

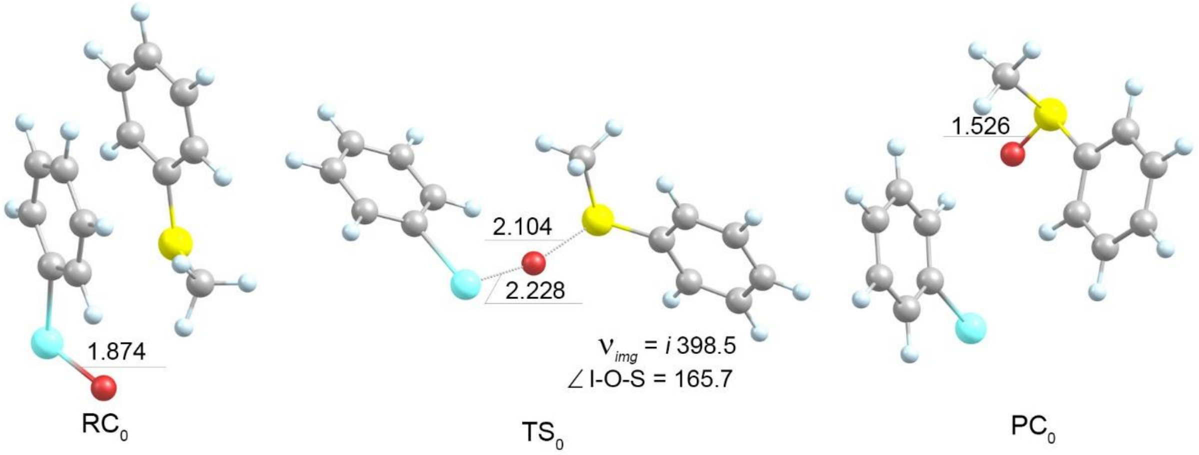

Supplementary Figure 10. The geometric information of the reaction intermediates in thioanisole sulfoxidation by the PhIO molecule. Lengths are in Å units, bond angles are in degree units and the imaginary frequency is in cm^{-1} units. Calculations were done at the Calculations were done at the UB3LYP-D3(BJ)/Lanl2dzdp(I), 6-31G*(S), 6-31G**(PhO), 6-31G(rest) level.

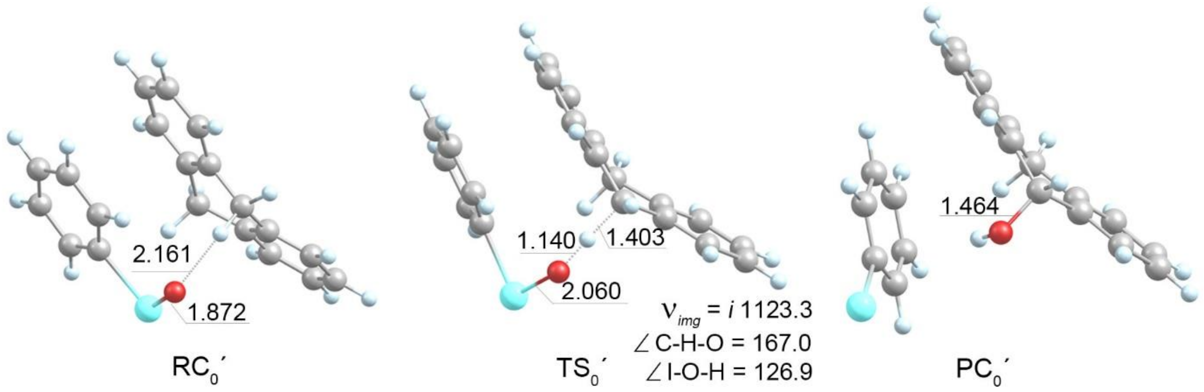

Supplementary Figure 11. The geometric information of the reaction intermediates in the C-H bond activation of 9,10-dihydroanthracene mediated by PhIO. Length is in Å, bond angle is in degree and imaginary frequency is in cm^{-1} unit. Calculations were done at the UB3LYP-D3(BJ)/Lanl2dzdp(I), 6-31G**(PhO), 6-31G(rest) level.

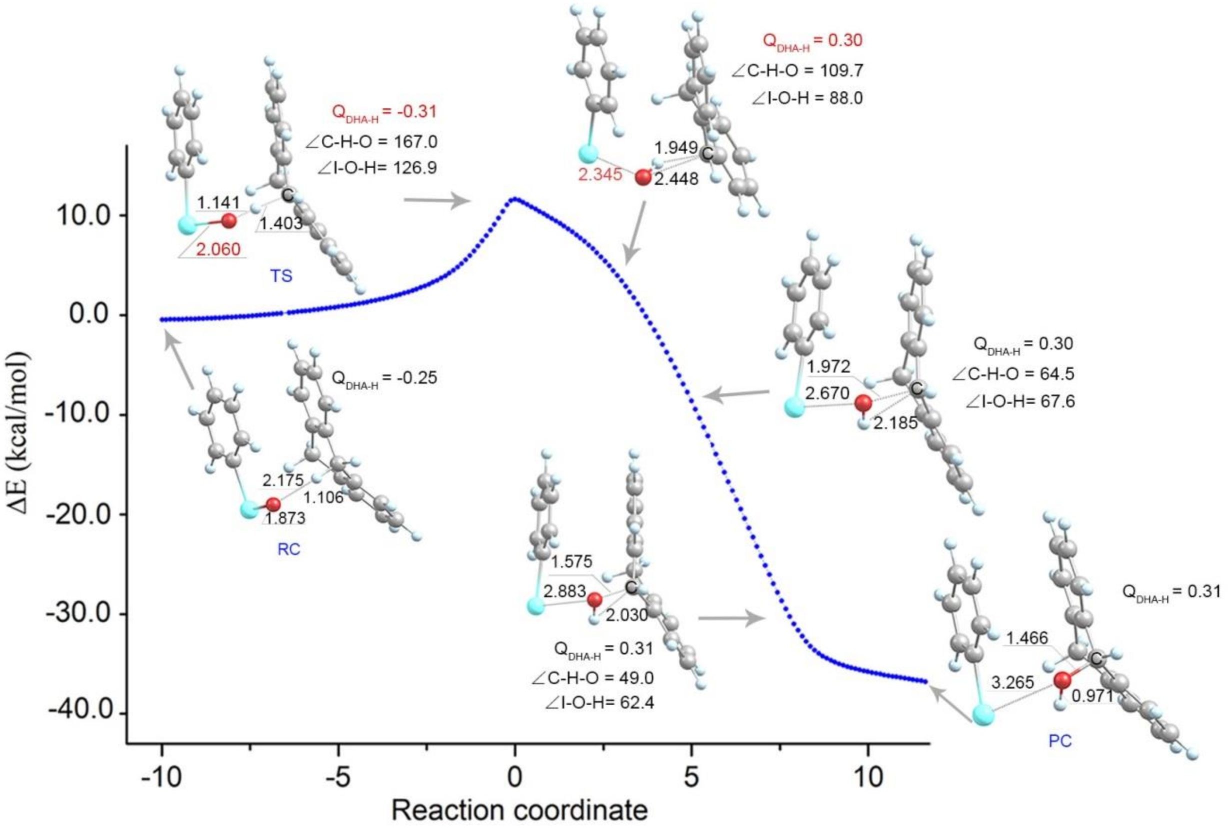

Supplementary Figure 12. Intrinsic reaction coordinate scan starting from the hydrogen abstraction transition state leading back to reactants or forward to products. All energies are in kcal mol^{-1} relative to the reactant complex. Also shown are geometries of critical points along the IRC.

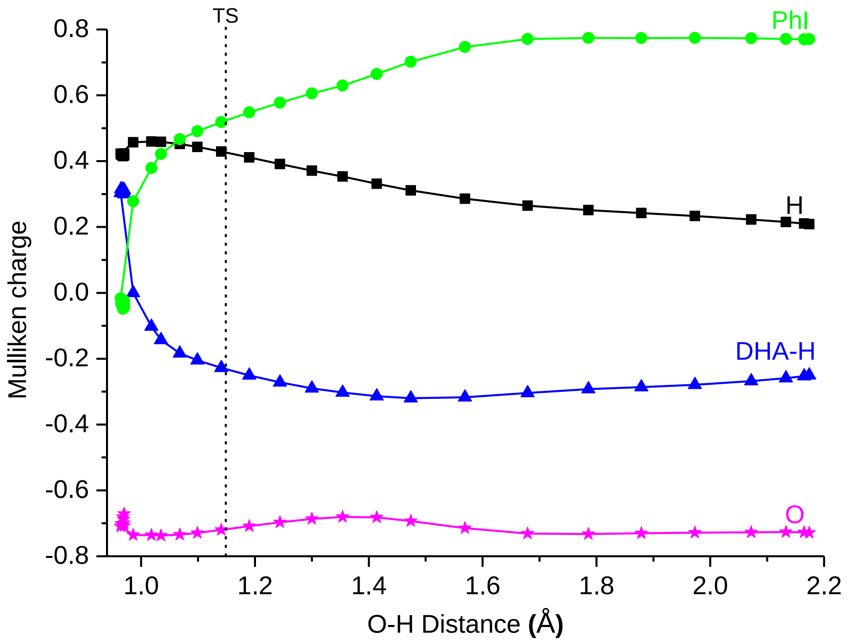

Supplementary Figure 13. The mulliken charge distribution of PhI, H, DHA-H and O atom as the change of O-H distance. The geometries of critical points are along the IRC.

2 Supplementary Tables

Supplementary Table 1. Comparison of the crystal structure and the optimized structure of **1**.

	Crystal srtucture	Optimized structure
Bond length(Å)		
Mn-N$_{avg}$	2.208	2.202
Mn-OIPh	1.904(4)	1.904
Mn-OH	1.832(4)	1.837
O-I	1.929(5)	1.938
Angles(°)		
Mn-O-I	116.2	116.6
O-Mn-OH	89.0	87.1

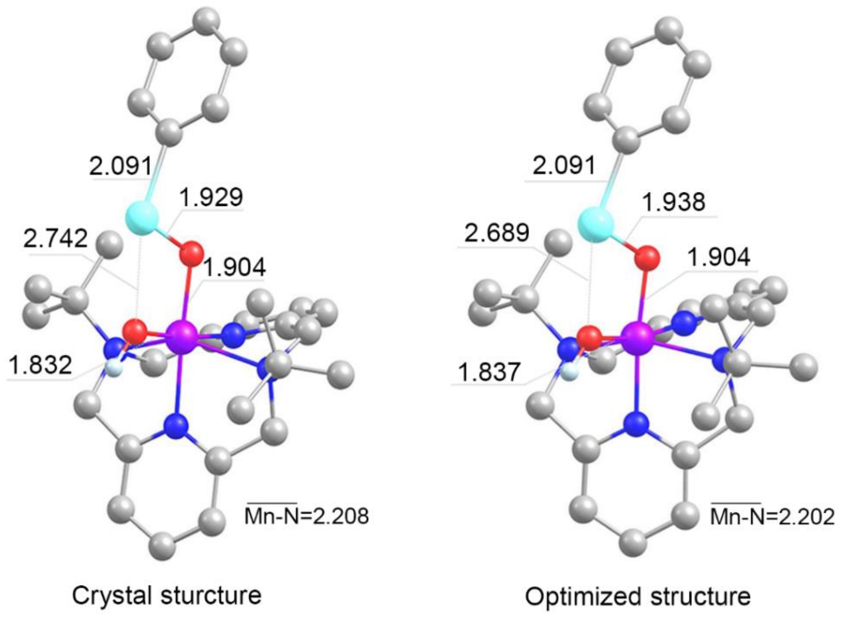

Supplementary Table 2. Various SCF and free energies of reaction intermediates in the conversion of the manganese-iodosylarene complex **1** to the high-valent manganese-oxo complex **2** in the absence of thioanisole.

	OPT/B1	ΔE	OPT +ZPE	ΔE	SPE/B2	ΔE	SPE+ZPE	ΔE	G	ΔG
^{5}**1**	-1574.306844	0.0	-1573.676613	0.0	-1574.975513	0.0	-1574.345282	0.0	-1573.741712	0.0
^{3}**1**	-1574.277044	18.7	-1573.644683	20.0	-1574.946285	18.3	-1574.313925	19.7	-1573.708534	20.8
^{1}**1**	-1574.234729	45.3	-1573.601867	46.9	-1574.903380	45.3	-1574.270518	46.9	-1573.663166	49.3
^{5}**TS**$_{12}$	-1574.271514	22.2	-1573.640332	22.8	-1574.944669	19.4	-1574.313487	20.0	-1573.702705	24.5
^{3}**TS**$_{12}$	-1574.274490	20.3	-1573.642844	21.2	-1574.944430	19.5	-1574.312784	20.4	-1573.705503	22.7
^{5}**2'**	-1574.278674	17.7	-1573.644207	20.3	-1574.950712	15.6	-1574.316245	18.2	-1573.708766	20.7
^{3}**2'**	-1574.282198	15.5	-1573.649682	16.9	-1574.951255	15.2	-1574.318738	16.7	-1573.712703	18.2
^{5}**2**	-1574.268004	24.4	-1573.636349	25.3	-1574.936011	24.8	-1574.304357	25.7	-1573.702356	24.7
^{3}**2**	-1574.272259	21.7	-1573.640176	22.9	-1574.940611	21.9	-1574.308528	23.1	-1573.702739	24.5
^{1}**2**	-1574.251151	34.9	-1573.617010	37.4	-1574.916868	36.8	-1574.282726	39.3	-1573.680320	38.5

a. Absolute energies are in au units and the relative ones are in kcal mol^{-1} units.

Supplementary Table 3. DFT functional and basis set benchmark on complex **1**. Absolute energies are in a.u. and the relative ones are in kcal mol^{-1}. Calculated were done at the UB3LYP-D3(BJ)/B1 level.The functions employ UB3LYP, UPBE0, UB3PW91, UM06, and UBP86 respectively.

		OPT/B1	ΔE	OPT +ZPE	ΔE	G	ΔE	H	ΔE
UB3LYP	^{5}**1**	-1574.306844	0.0	-1573.676613	0.0	-1573.741712	0.0	-1573.641151	0.0
	^{3}**1**	-1574.277044	18.7	-1573.644683	20.0	-1573.708534	20.8	-1573.610070	19.5
	^{1}**1**	-1574.234729	45.3	-1573.601867	46.9	-1573.663166	49.3	-1573.567554	46.2
UPBE0	^{5}**1**	-1572.484174	0.0	-1571.849024	0.0	-1571.913790	0.0	-1571.813979	0.0
	^{3}**1**	-1572.449886	21.5	-1571.813470	22.3	-1571.876724	23.3	-1571.779091	21.9
	^{1}**1**	-1572.433097	32.1	-1571.796787	32.8	-1571.858311	34.8	-1571.762389	32.4
UB3PW91	^{5}**1**	-1573.834459	0.0	-1573.201467	0.0	-1573.266223	0.0	-1573.166331	0.0
	^{3}**1**	-1573.803047	19.7	-1573.168500	20.7	-1573.230551	22.4	-1573.134233	20.1
	^{1}**1**	-1573.785623	30.6	-1573.151598	31.3	-1573.213153	33.3	-1573.117152	30.9
UM06	^{5}**1**	-1573.103994	0.0	-1572.476399	0.0	-1572.540589	0.0	-1572.441164	0.0
	^{3}**1**	-1573.043693	37.8	-1572.414195	39.0	-1572.475376	40.9	-1572.380342	38.2
	^{1}**1**	-1573.019801	52.8	-1572.391128	53.5	-1572.452954	55.0	-1572.356374	53.2
UBP86	^{5}**1**	-1574.417056	11.4	-1573.805751	10.0	-1573.871285	8.8	-1573.769508	10.4
	^{3}**1**	-1574.435286	0.0	-1573.821722	0.0	-1573.885255	0.0	-1573.786036	0.0
	^{1}**1**	-1574.429765	3.5	-1573.814037	4.8	-1573.878864	4.0	-1573.778071	5.0

Supplementary Table 4. Mulliken spin densities and charges of reaction intermediates in the formation of high-valent manganese-oxo complex **2** from complex **1**. Calculated were done at the UB3LYP-D3(BJ)/B1 level.

	Spin density					Charge				
	Mn	O	PhI	TBDAP	OH	Mn	O	PhI	TBDAP	OH
^{5}**1**	3.79	0.02	0.03	0.11	0.05	1.06	-0.72	1.01	1.04	-0.38
^{3}**1**	1.99	0.05	-0.03	-0.07	0.06	0.97	-0.69	0.90	1.20	-0.37
^{1}**1**	0.00	0.00	0.00	0.00	0.00	0.92	-0.68	0.92	1.20	-0.35
^{5}**TS**$_{12}$	2.97	0.39	0.61	-0.08	0.10	1.03	-0.68	0.67	1.28	-0.30
^{3}**TS**$_{12}$	2.22	0.08	-0.29	-0.11	0.09	0.99	-0.63	0.70	1.28	-0.34
^{5}**2'**	2.81	0.65	0.58	-0.15	0.12	0.98	-0.60	0.54	1.37	-0.29
^{3}**2'**	2.64	-0.30	-0.35	-0.13	0.14	1.01	-0.51	0.34	1.43	-0.28
^{5}**2**	2.66	1.09	0.02	-0.06	0.29	1.02	-0.37	0.06	1.55	-0.25
^{3}**2**	2.65	-0.61	-0.06	-0.16	0.17	0.97	-0.38	0.07	1.58	-0.25
^{1}**2**	0.00	0.00	0.00	0.00	0.00	0.83	-0.32	0.04	1.70	-0.25

Supplementary Table 5. Various SCF and free energies of the reaction intermediates in thioanisole sulfoxidation by 1 via the direct oxygen-atom transfer mechanism.

	OPT/B1	ΔE	OPT +ZPE	ΔE	SPE/B2	ΔE	SPE+ZPE	ΔE	G	ΔG
^{5}RC	-2244.048219	0.0	-2243.285255	0.0	-2244.881697	0.0	-2244.118733	0.0	-2243.362637	0.0
^{3}RC	-2244.022287	16.3	-2243.256452	18.1	-2244.854439	17.1	-2244.088604	18.9	-2243.333388	18.4
$^{5}TS_{DOT}$	-2244.003068	28.3	-2243.240776	27.9	-2244.838326	27.2	-2244.076035	26.8	-2243.315821	29.4
$^{3}TS_{DOT}$	-2243.969007	49.7	-2243.202275	52.1	-2244.800063	51.2	-2244.033330	53.6	-2243.275099	54.9
^{5}PC	-2244.108420	-37.8	-2243.343908	-36.8	-2244.938002	-35.3	-2244.169586	-31.9	-2243.419841	-35.9
^{3}PC	-2244.085247	-23.2	-2243.316831	-19.8	-2244.913999	-20.3	-2244.149487	-19.3	-2243.391837	-18.3

a. Absolute energies are in au units and the relative ones are in kcal mol^{-1} units.

Supplementary Table 6. Mulliken spin densities and charges of reaction intermediates in thioanisole sulfoxidation by complex 1 via the direct oxygen-atom transfer mechanism. Calculations were done at the UB3LYP/B1 level.

	Spin density						Charge					
	Mn	O	PhI	TBDAP	OH	Thio	Mn	O	PhI	TBDAP	OH	Thio
^{5}RC	3.82	0.00	0.04	0.09	0.05	0.00	1.04	-0.72	0.99	1.00	-0.35	0.05
^{3}RC	2.01	0.04	-0.03	-0.09	0.07	0.00	0.97	-0.70	0.93	1.16	-0.37	0.01
$^{5}TS_{DOT}$	3.86	-0.01	0.03	0.10	0.04	-0.01	1.06	-0.70	0.94	1.03	-0.37	0.04
$^{3}TS_{DOT}$	2.08	-0.01	0.00	-0.14	0.08	-0.01	0.95	-0.67	0.92	1.17	-0.36	0.00
^{5}PC	3.82	0.01	0.00	0.10	0.06	0.01	0.99	-0.70	0.00	2.05	-0.34	0.00
^{3}PC	1.98	-0.01	0.00	-0.08	0.12	0.00	0.91	-0.67	0.00	2.11	-0.34	0.00

Supplementary Table 7. Various SCF and free energies of the reaction intermediates in thioanisole sulfoxidation by complex **1** via the electron transfer/oxygen transfer mechanism. Calculations were done at the UB3LYP-D3(BJ)/ B1 level.

	OPT/B1	ΔE	OPT +ZPE	ΔE	SPE/B2	ΔE	SPE+ZPE	ΔE	G	ΔG
^{5}RC	-2244.048219	0.0	-2243.285255	0.0	-2244.881697	0.0	-2244.118733	0.0	-2243.362637	0.0
^{3}RC	-2244.022287	16.3	-2243.256452	18.1	-2244.854439	17.1	-2244.088604	18.9	-2243.333388	18.4
^{5}TS1	-2244.018256	18.8	-2243.252815	20.4	-2244.850095	19.8	-2244.084654	21.4	-2243.328833	21.2
^{3}TS1	-2244.019478	18.0	-2243.253111	20.2	-2244.850083	19.8	-2244.083716	22.0	-2243.326985	22.4
^{5}IM1	-2244.018402	18.7	-2243.254021	19.6	-2244.851906	18.7	-2244.087525	19.6	-2243.328424	21.5
^{3}IM1	-2244.029956	11.5	-2243.263136	13.9	-2244.860319	13.4	-2244.093499	15.8	-2243.340247	14.0
$^{5}TS_{ET}$	-2244.016042	20.2	-2243.250798	21.6	-2244.846799	21.9	-2244.081555	23.3	-2243.327281	22.2
$^{3}TS_{ET}$	-2244.028344	12.5	-2243.262322	14.4	-2244.858217	14.7	-2244.092195	16.7	-2243.337561	15.7
^{5}IM2	-2244.031100	10.7	-2243.263988	13.3	-2244.862483	12.1	-2244.095371	14.7	-2243.340739	13.7
^{3}IM2	-2244.037041	7.0	-2243.269271	10.0	-2244.867324	9.0	-2244.099554	12.0	-2243.345266	10.9
$^{5}TS_{OT}$	-2244.027573	13.0	-2243.262292	14.4	-2244.860036	13.6	-2244.094755	15.0	-2243.338618	15.1
$^{3}TS_{OT}$	-2244.028971	12.1	-2243.262275	14.4	-2244.860254	13.5	-2244.093559	15.8	-2243.337483	15.8
^{5}PC	-2244.101168	-33.2	-2243.337200	-32.6	-2244.931634	-31.3	-2244.167665	-30.7	-2243.414652	-32.6
^{3}PC	-2244.085699	-23.5	-2243.317703	-20.4	-2244.914220	-20.4	-2244.146223	-17.3	-2243.392736	-18.9

a. Absolute energies are in au units and the relative ones are in kcal mol^{-1} units.

Supplementary Table 8. Mulliken spin densities and charges of reaction intermediates in thioanisole sulfoxidation by complex 1 via electron transfer/oxygen transfer mechanism. Calculations were done at the UB3LYP/B1 level.

	Spin density						Charge					
	Mn	O	PhI	TBDAP	OH	Thio	Mn	O	PhI	TBDAP	OH	Thio
^{5}RC	3.82	0.00	0.04	0.09	0.05	0.00	1.04	-0.72	0.99	1.00	-0.35	0.05
^{3}RC	2.01	0.04	-0.03	-0.09	0.07	0.00	0.97	-0.70	0.93	1.16	-0.37	0.01
^{5}TS1	2.97	0.50	0.58	-0.15	0.10	0.01	1.02	-0.63	0.55	1.31	-0.30	0.04
^{3}TS1	2.25	0.05	-0.26	-0.13	0.09	0.00	0.97	-0.65	0.75	1.22	-0.33	0.04
^{5}IM1	2.82	0.64	0.58	-0.16	0.12	0.01	1.01	-0.59	0.50	1.34	-0.30	0.04
^{3}IM1	2.75	-0.23	-0.43	-0.20	0.12	-0.01	1.00	-0.55	0.39	1.40	-0.29	0.05
$^{5}TS_{ET}$	2.63	0.68	0.38	-0.12	0.11	0.31	0.98	-0.56	0.32	1.26	-0.31	0.31
$^{3}TS_{ET}$	2.76	-0.33	-0.24	-0.19	0.12	-0.13	1.00	-0.48	0.22	1.40	-0.28	0.15
^{5}IM2	2.68	0.62	0.00	-0.11	0.11	0.69	0.97	-0.60	0.02	1.25	-0.30	0.67
^{3}IM2	2.67	-0.08	0.00	-0.15	0.11	-0.55	0.98	-0.55	0.01	1.30	-0.29	0.54
$^{5}TS_{OT}$	2.91	0.41	0.01	-0.10	0.09	0.69	1.01	-0.64	-0.02	1.23	-0.31	0.73
$^{3}TS_{OT}$	2.41	0.09	0.00	-0.12	0.12	-0.50	0.99	-0.60	-0.03	1.27	-0.30	0.67
^{5}PC	3.82	0.02	0.00	0.09	0.06	0.01	1.00	-0.69	-0.04	1.05	-0.33	0.99
^{3}PC	2.04	0.00	0.00	-0.13	0.09	0.00	0.89	-0.65	-0.04	1.20	-0.34	0.94

Supplementary Table 9. Various SCF and free energies of the reaction intermediates along the C-H bond activation of 9,10-dihydroanthracene mediated by complex **1.** Calculations were done at the UB3LYP-D3(BJ)/B1 level.

	OPT/B1	ΔE	OPT +ZPE	ΔE	SPE/B2	ΔE	SPE+ZPE	ΔE	G	ΔG
^5RC_H	-2115.011248	0.0	-2114.159678	0.0	-2115.937251	0.0	-2115.085680	0.0	-2114.238116	0.0
^3RC_H	-2114.984003	17.1	-2114.128946	19.3	-2115.907372	18.7	-2115.052314	20.9	-2114.206683	19.7
$^5TS1_H$	-2114.980651	19.2	-2114.134384	15.9	-2115.908233	18.2	-2115.061966	14.9	-2114.210706	17.2
$^3TS1_H$	-2114.936286	47.0	-2114.087530	45.3	-2115.859160	49.0	-2115.010403	47.2	-2114.161077	48.3
^5IM_H	-2115.062862	-32.4	-2114.213169	-33.6	-2115.990693	-33.5	-2115.141000	-34.7	-2114.294881	-35.6
^3IM_H	-2115.036146	-15.6	-2114.182859	-14.5	-2115.960024	-14.3	-2115.106737	-13.2	-2114.264368	-16.5
$^5TS2_H$	-2115.054463	-27.1	-2114.207985	-30.3	-2115.982168	-28.2	-2115.135690	-31.4	-2114.287847	-31.2
$^3TS2_H$	-2115.036907	-16.1	-2114.187429	-17.4	-2115.961103	-15.0	-2115.111625	-16.3	-2114.265849	-17.4
$^5TS2_{reb}$	-2115.054124	-26.9	-2114.202655	-27.0	-2115.982476	-28.4	-2115.131007	-28.4	-2114.282626	-27.9
^5PC_H	-2115.094100	-52.0	-2114.243691	-52.7	-2116.024848	-55.0	-2115.174440	-55.7	-2114.325754	-55.0
^3PC_H	-2115.067268	-35.2	-2114.212173	-32.9	-2115.994911	-36.2	-2115.139816	-34.0	-2114.293074	-34.5
$^5PC_{reb}$	-2115.075349	-40.2	-2114.221344	-38.7	-2116.004821	-42.4	-2115.150816	-40.9	-2114.300067	-38.9

a. Absolute energies are in au units and the relative ones are in kcal mol^{-1} units.

Supplementary Table 10. Mulliken spin densities and charges of the reaction intermediates along C-H bond activation of 9,10-dihydroanthracene by complex **1.** Calculations were done at the UB3LYP/B1 level.

	Spin density							Charge						
	Mn	O	PhI	TBDAP	OH	H_{Step-1}	DHA-H	Mn	O	PhI	TBDAP	OH	H_{Step-1}	DHA-H
$^{5}RC_{H}$	3.80	0.01	0.03	0.11	0.05	0.00	0.00	1.08	-0.73	0.98	1.02	-0.37	0.18	-0.16
$^{3}RC_{H}$	2.00	0.02	-0.01	-0.09	0.07	0.00	0.00	0.97	-0.70	0.92	1.15	-0.36	0.18	-0.16
$^{5}TS1_{H}$	3.86	-0.02	-0.04	0.08	0.04	0.01	0.07	0.99	-0.67	0.60	0.99	-0.36	0.35	0.09
$^{3}TS1_{H}$	2.00	0.02	-0.01	-0.09	0.07	0.00	0.00	1.02	-0.65	-0.03	1.36	-0.28	0.35	0.23
$^{5}IM_{H}$	3.79	0.06	0.00	0.08	0.06	0.00	0.00	0.96	-0.80	-0.05	0.90	-0.38	0.42	0.95
$^{3}IM_{H}$	1.96	0.06	0.00	-0.06	0.03	0.00	0.00	0.88	-0.77	-0.05	1.01	-0.38	0.41	0.90
	Spin density							Charge						
	Mn	OH_{step-1}	PhI	TBDAP	OH	H_{Step-2}	DHA-H	Mn	OH_{step-1}	PhI	TBDAP	OH	H_{Step-2}	DHA-H
$^{5}TS2_{H}$	3.81	0.03	0.00	0.09	0.06	0.00	0.00	0.99	-0.36	-0.05	1.00	-0.36	0.40	0.38
$^{3}TS2_{H}$	1.98	0.00	0.00	-0.09	0.11	0.00	0.00	0.92	-0.32	-0.04	1.16	-0.34	0.40	0.23
$^{5}TS2_{reb}$	3.82	0.02	0.00	0.09	0.06	0.00	0.02	0.97	-0.28	-0.05	1.05	-0.35	0.22	0.44
$^{5}PC_{H}$	3.82	0.02	0.00	0.11	0.06	0.00	0.00	1.03	-0.32	0.05	1.04	-0.33	0.45	0.09
$^{3}PC_{H}$	2.04	0.01	0.00	-0.13	0.08	0.00	0.00	0.91	-0.31	0.03	1.19	-0.35	0.45	0.08
$^{5}PC_{reb}$	3.82	0.01	0.00	0.09	0.06	0.00	0.01	1.01	-0.25	0.04	1.04	-0.35	0.20	0.30

Supplementary Table 11. Various SCF and free energies of the reaction intermediates in thioanisole sulfoxidation by the PhIO molecule. Calculations were done at the UB3LYP-D3(BJ)/Lanl2dzdp(I), 6-31G*(S), 6-31G** (PhO), 6-31G(rest) level, UB3LYP-D3(BJ)/ Lanl2dztp(I), 6-311+G**(C,H,O,S) level for single point energies.

	OPT	ΔE	OPT +ZPE	ΔE	SPE	ΔE	SPE+ZPE	ΔE	G	ΔG
RC_0	-987.905993	0.0	-987.681619	0.0	-988.158339	0.0	-987.933965	0.0	-987.729203	0.0
TS_0	-987.882791	14.6	-987.659257	14.0	-988.139865	11.6	-987.916331	11.1	-987.707931	13.3
PC_0	-987.978211	-45.3	-987.752311	-44.4	-988.221767	-39.8	-987.995866	-38.8	-987.801199	-45.2

a. Absolute energies are in au units and the relative ones are in kcal mol^{-1} units.

Supplementary Table 12. Mulliken spin densities and charges of the reaction intermediates in thioanisole sulfoxidation by PhIO molecule. Calculations were done at the UB3LYP-D3(BJ)/Lanl2dzdp(I), 6-31G*(S), 6-31G**(PhO), 6-31G(rest) level.

	Spin densities			Charge		
	O	PhI	Thio	O	PhI	Thio
RC_0	0.00	0.00	0.00	-0.75	0.77	-0.03
TS_0	0.00	0.00	0.00	-0.62	0.39	0.23
PC_0	0.00	0.00	0.00	-0.67	0.02	0.71

Supplementary Table 13. Various SCF and free energies of the reaction intermediates of C-H bond activation by the PhIO molecule. Calculations were done at the UB3LYP-D3(BJ)/Lanl2dzdp(I), 6-31G**(PhO), 6-31G(rest) level, UB3LYP-D3(BJ)/Lanl2dzdp(I), 6-311+G**(C,H,O) level for single point energies.

	OPT	ΔE	OPT +ZPE	ΔE	SPE	ΔE	SPE+ZPE	ΔE	G	ΔG
RC_0'	-858.864177	0.0	-858.550993	0.0	-859.207819	0.0	-858.894635	0.0	-858.600821	0.0
TS_0'	-858.833429	19.3	-858.526695	15.2	-859.181597	16.5	-858.874863	12.4	-858.574779	16.3
PC_0'	-858.958316	-59.1	-858.643069	-57.8	-859.307190	-62.4	-858.991943	-61.1	-858.694873	-59.0

a. Absolute energies are in au units and the relative ones are in kcal mol^{-1} units.

Supplementary Table 14. Mulliken spin densities and charges of the reaction intermediates of C-H bond activation by PhIO molecule. Calculations were done at the UB3LYP-D3(BJ)/Lanl2dzdp(I), 6-31G**(PhO), 6-31G(rest) level.

	Spin densities				Charge			
	O	PhI	H	DHA-H	O	PhI	H	DHA-H
RC_0'	0.00	0.00	0.00	0.00	-0.73	0.77	0.21	-0.25
TS_0'	0.00	0.00	0.00	0.00	-0.72	0.52	0.43	-0.23
PC_0'	0.00	0.00	0.00	0.00	-0.71	0.00	0.41	0.30

Supplementary Table 15. The mulliken charge distribution of DHA-H, H, PhI and O atom as the change of O-H distance. The geometries of critical points are along the IRC. Lengths are in Å units.

$Dis_{O\text{-}H}$	DHA-H	H	PhI	O
2.17	-0.25	0.21	0.77	-0.73
2.16	-0.25	0.21	0.77	-0.73
2.13	-0.26	0.22	0.77	-0.73
2.07	-0.27	0.22	0.77	-0.73
1.97	-0.28	0.23	0.77	-0.73
1.88	-0.29	0.24	0.77	-0.73
1.79	-0.29	0.25	0.77	-0.73
1.68	-0.30	0.27	0.77	-0.73
1.57	-0.32	0.29	0.75	-0.72
1.47	-0.32	0.31	0.70	-0.69
1.41	-0.31	0.33	0.67	-0.68
1.35	-0.30	0.35	0.63	-0.68
1.30	-0.29	0.37	0.61	-0.69
1.24	-0.27	0.39	0.58	-0.70
1.19	-0.25	0.41	0.55	-0.71
1.14	-0.23	0.43	0.52	-0.72
1.10	-0.21	0.44	0.49	-0.73
1.06	-0.18	0.45	0.47	-0.73
1.04	-0.14	0.46	0.42	-0.74
1.02	-0.10	0.46	0.38	-0.74
0.99	-0.09	0.46	0.28	-0.74
0.97	0.30	0.42	-0.05	-0.67
0.96	0.31	0.42	-0.04	-0.69
0.96	0.31	0.42	-0.05	-0.68
0.96	0.30	0.42	-0.02	-0.71
0.96	0.32	0.42	-0.03	-0.70

3 Supplementary Cartesian Coordinates

A. The formation of the high-valent iron-oxo complex in the absence of thioanisol

^{5}1

Mn	-0.167510000	0.245842000	-0.010925000
N	-0.204160000	0.155808000	2.358680000
C	1.145859000	0.218576000	3.098121000
C	1.726162000	1.637203000	2.934185000
H	1.789331000	1.905197000	1.876769000
H	1.149672000	2.399246000	3.464002000
H	2.738792000	1.648909000	3.347975000
C	0.962447000	-0.118299000	4.593666000
H	0.683224000	-1.165950000	4.739636000
H	0.207974000	0.512529000	5.073880000
H	1.909936000	0.047525000	5.114836000
C	2.140020000	-0.767099000	2.463706000
H	3.045842000	-0.793617000	3.077043000
H	2.417129000	-0.424207000	1.467987000
H	1.757245000	-1.788423000	2.402535000
C	-0.909310000	-1.160975000	2.516228000
H	-1.265793000	-1.313734000	3.540319000
H	-0.198672000	-1.955061000	2.283044000
C	-2.079430000	-1.239848000	1.566315000
C	-3.243922000	-1.958551000	1.824954000
H	-3.337606000	-2.525954000	2.741782000
C	-4.284744000	-1.918096000	0.889489000
H	-5.192548000	-2.482000000	1.065321000
C	-4.167197000	-1.120407000	-0.252833000
H	-4.979912000	-1.040285000	-0.963246000
C	-2.988928000	-0.401994000	-0.460575000
N	-1.969008000	-0.519749000	0.421100000
C	-2.841641000	0.619450000	-1.560001000
H	-3.455207000	0.331169000	-2.414017000
H	-3.270879000	1.555711000	-1.185121000
N	-1.420384000	0.875788000	-1.968819000
C	-1.061441000	0.181569000	-3.296530000
C	-1.935641000	0.697468000	-4.462191000
H	-2.993948000	0.449161000	-4.342429000
H	-1.594096000	0.224804000	-5.388406000
H	-1.842864000	1.780129000	-4.589992000
C	0.416632000	0.466671000	-3.633321000
H	0.543720000	1.445447000	-4.106275000
H	0.773088000	-0.285070000	-4.343043000
H	1.046214000	0.433124000	-2.743920000
C	-1.281201000	-1.330987000	-3.111382000
H	-0.694080000	-1.714979000	-2.275249000
H	-0.978075000	-1.849413000	-4.025789000
H	-2.332808000	-1.574164000	-2.933260000
C	-1.113878000	2.343524000	-1.978387000
H	-1.783522000	2.899577000	-2.643700000
H	-0.098178000	2.471193000	-2.355413000
C	-1.218502000	2.913786000	-0.585796000
C	-1.626185000	4.217505000	-0.312628000
H	-1.855570000	4.893495000	-1.126031000
C	-1.750911000	4.621985000	1.021562000
H	-2.051591000	5.635945000	1.254834000
C	-1.532669000	3.701907000	2.051272000
H	-1.681220000	3.977572000	3.087435000
C	-1.147039000	2.400699000	1.727931000
N	-0.939829000	2.061408000	0.432987000
C	-1.104932000	1.289378000	2.745965000
H	-0.852489000	1.687770000	3.729607000
H	-2.127844000	0.906340000	2.831659000
O	1.526468000	0.781437000	-0.479622000
H	1.767021000	1.712007000	-0.573752000
O	0.419064000	-1.540695000	-0.307242000
I	2.188778000	-1.756989000	-1.069408000
C	1.960598000	-3.825792000	-1.273928000
C	1.429732000	-4.327767000	-2.467586000
H	1.164957000	-3.665645000	-3.283698000
C	1.243633000	-5.705159000	-2.579457000
H	0.832681000	-6.115947000	-3.495411000
C	1.583600000	-6.548788000	-1.518537000
H	1.435708000	-7.619284000	-1.614618000
C	2.113440000	-6.026875000	-0.335389000
H	2.375775000	-6.686882000	0.484463000
C	2.309479000	-4.652839000	-0.200391000
H	2.718259000	-4.239195000	0.714367000

^{3}1

Mn	-0.256647000	0.275996000	0.030358000
N	-0.202390000	0.124298000	2.199990000
C	1.169037000	0.226782000	2.945138000
C	1.694446000	1.667417000	2.803840000
H	1.743744000	1.954279000	1.751601000
H	1.098224000	2.398259000	3.354861000
H	2.708558000	1.702202000	3.212740000
C	0.993206000	-0.143050000	4.435522000
H	0.794090000	-1.209905000	4.567502000
H	0.203081000	0.424183000	4.934580000
H	1.930121000	0.082476000	4.952680000
C	2.199060000	-0.720738000	2.318251000
H	3.075708000	-0.754797000	2.972104000
H	2.512606000	-0.339310000	1.350256000
H	1.831479000	-1.742796000	2.208558000
C	-0.869025000	-1.221970000	2.421680000
H	-1.151106000	-1.350049000	3.469350000
H	-0.152617000	-1.996982000	2.151356000
C	-2.080125000	-1.309956000	1.533144000
C	-3.231911000	-2.051749000	1.777640000
H	-3.311790000	-2.661801000	2.667801000
C	-4.282255000	-1.980598000	0.850695000
H	-5.182727000	-2.560075000	1.012773000
C	-4.184281000	-1.144808000	-0.267862000
H	-5.000303000	-1.058031000	-0.973335000
C	-3.013533000	-0.407332000	-0.450023000
N	-1.998912000	-0.538325000	0.425691000
C	-2.801392000	0.635390000	-1.506684000
H	-3.367963000	0.400750000	-2.404713000
H	-3.200812000	1.581406000	-1.128353000
N	-1.326696000	0.843270000	-1.839981000
C	-0.972830000	0.133992000	-3.186996000

C	-1.859565000	0.639747000	-4.350284000
H	-2.907244000	0.342960000	-4.261952000
H	-1.477138000	0.190591000	-5.272017000
H	-1.807904000	1.724953000	-4.471460000
C	0.492075000	0.447671000	-3.552185000
H	0.574965000	1.399750000	-4.085046000
H	0.870118000	-0.332408000	-4.218141000
H	1.129801000	0.492107000	-2.672415000
C	-1.203416000	-1.374244000	-2.995840000
H	-0.646398000	-1.761163000	-2.142940000
H	-0.882473000	-1.897668000	-3.900948000
H	-2.262064000	-1.605301000	-2.845364000
C	-1.033796000	2.329432000	-1.915038000
H	-1.692740000	2.825373000	-2.632420000
H	-0.008647000	2.451248000	-2.263771000
C	-1.200560000	2.931644000	-0.549641000
C	-1.614141000	4.231296000	-0.272138000
H	-1.811595000	4.925310000	-1.078502000
C	-1.784545000	4.608321000	1.067404000
H	-2.092740000	5.618079000	1.308563000
C	-1.595860000	3.673179000	2.091176000
H	-1.768132000	3.935891000	3.126792000
C	-1.202363000	2.378953000	1.750961000
N	-0.968877000	2.061961000	0.460307000
C	-1.133884000	1.221876000	2.699912000
H	-0.851611000	1.543239000	3.700486000
H	-2.140906000	0.802695000	2.782157000
O	1.470912000	0.793048000	-0.437936000
H	1.680584000	1.690126000	-0.727724000
O	0.389363000	-1.463364000	-0.209073000
I	2.138339000	-1.643208000	-1.091601000
C	1.870592000	-3.718478000	-1.253398000
C	1.394498000	-4.248244000	-2.457138000
H	1.192307000	-3.606956000	-3.307236000
C	1.178522000	-5.624302000	-2.540204000
H	0.809968000	-6.052398000	-3.466439000
C	1.431062000	-6.443098000	-1.437004000
H	1.258664000	-7.511931000	-1.508715000
C	1.905734000	-5.895936000	-0.241953000
H	2.101936000	-6.535308000	0.612302000
C	2.133832000	-4.523627000	-0.140119000
H	2.504401000	-4.094115000	0.783751000

^{1}**1**

Mn	-0.225897000	0.269859000	-0.013269000
N	-0.261855000	0.094725000	2.131186000
C	1.118887000	0.200671000	2.877377000
C	1.631764000	1.648583000	2.778391000
H	1.687911000	1.971457000	1.737261000
H	1.025874000	2.359242000	3.344300000
H	2.642447000	1.678724000	3.195620000
C	0.941765000	-0.205992000	4.359377000
H	0.764262000	-1.279147000	4.466151000
H	0.138717000	0.333868000	4.867138000
H	1.871930000	0.027067000	4.885137000
C	2.166877000	-0.720102000	2.241332000
H	3.034318000	-0.758510000	2.906827000
H	2.494468000	-0.314309000	1.287480000
H	1.813293000	-1.743006000	2.103600000
C	-0.900352000	-1.269164000	2.342833000
H	-1.192702000	-1.408469000	3.385778000
H	-0.161206000	-2.023383000	2.076174000
C	-2.095432000	-1.356959000	1.439383000
C	-3.267636000	-2.068433000	1.681214000
H	-3.356037000	-2.694417000	2.559558000
C	-4.330548000	-1.932426000	0.777458000
H	-5.248909000	-2.483914000	0.936779000
C	-4.224926000	-1.048984000	-0.303130000
H	-5.055823000	-0.888703000	-0.977797000
C	-3.028667000	-0.354180000	-0.483459000
N	-1.989409000	-0.568931000	0.346257000
C	-2.806862000	0.758718000	-1.461411000
H	-3.406471000	0.633008000	-2.359709000
H	-3.144526000	1.686811000	-0.990297000
N	-1.335560000	0.909849000	-1.801619000
C	-1.020520000	0.165260000	-3.150011000
C	-1.931097000	0.674506000	-4.295896000
H	-2.971943000	0.358068000	-4.199021000
H	-1.552823000	0.243219000	-5.227675000
H	-1.897581000	1.761911000	-4.403148000
C	0.436158000	0.444268000	-3.571557000
H	0.519239000	1.392138000	-4.111797000
H	0.763925000	-0.347115000	-4.250930000
H	1.113237000	0.476148000	-2.721992000
C	-1.275018000	-1.336630000	-2.941838000
H	-0.698135000	-1.732705000	-2.106190000
H	-0.993195000	-1.867097000	-3.856089000
H	-2.332671000	-1.545744000	-2.758496000
C	-0.969767000	2.380942000	-1.899281000
H	-1.615821000	2.903785000	-2.608853000
H	0.054534000	2.442256000	-2.263030000
C	-1.094625000	2.981746000	-0.531032000
C	-1.508943000	4.277802000	-0.236218000
H	-1.691352000	4.986725000	-1.033267000
C	-1.715754000	4.625828000	1.105547000
H	-2.027293000	5.630716000	1.362313000
C	-1.576742000	3.658812000	2.107369000
H	-1.799723000	3.888816000	3.141257000
C	-1.173288000	2.373732000	1.745412000
N	-0.872780000	2.088647000	0.461372000
C	-1.186253000	1.179145000	2.648843000
H	-0.946076000	1.443006000	3.677239000
H	-2.205787000	0.783162000	2.658522000
O	1.441509000	0.773690000	-0.565708000
H	1.760265000	1.683844000	-0.640199000
O	0.413992000	-1.488409000	-0.254505000
I	2.152852000	-1.682371000	-1.134133000
C	1.938584000	-3.766537000	-1.214309000
C	1.334208000	-4.341692000	-2.337499000
H	1.005688000	-3.729650000	-3.169480000
C	1.157711000	-5.725163000	-2.362362000
H	0.690091000	-6.188757000	-3.224531000
C	1.579681000	-6.507217000	-1.284156000
H	1.438570000	-7.582663000	-1.311386000
C	2.183760000	-5.915357000	-0.172005000
H	2.511024000	-6.526317000	0.662596000
C	2.370857000	-4.533662000	-0.127328000

H	2.838727000	-4.068506000	0.732863000

$^{5}TS_{12}$

Mn	0.226697000	0.369566000	-0.295071000
N	0.469246000	-0.091448000	1.843993000
C	1.825708000	0.316387000	2.507553000
C	1.874619000	1.847598000	2.651260000
H	1.781316000	2.328642000	1.678986000
H	1.112777000	2.240607000	3.328318000
H	2.848468000	2.118218000	3.068928000
C	1.948308000	-0.344912000	3.899053000
H	2.064373000	-1.429462000	3.828859000
H	1.099002000	-0.124349000	4.552240000
H	2.844997000	0.049628000	4.385093000
C	2.998853000	-0.138898000	1.624773000
H	3.927710000	0.164910000	2.117477000
H	2.951709000	0.324804000	0.640722000
H	3.035955000	-1.224402000	1.507108000
C	0.277302000	-1.596519000	1.849641000
H	0.128777000	-1.964931000	2.866948000
H	1.183924000	-2.047163000	1.450155000
C	-0.897164000	-1.946310000	0.980691000
C	-1.728342000	-3.052179000	1.128158000
H	-1.558990000	-3.761770000	1.925877000
C	-2.774931000	-3.228209000	0.212505000
H	-3.417595000	-4.095799000	0.291175000
C	-3.005585000	-2.280144000	-0.790659000
H	-3.826451000	-2.392066000	-1.486615000
C	-2.162920000	-1.171280000	-0.868757000
N	-1.130070000	-1.058558000	-0.011327000
C	-2.366056000	0.009201000	-1.769323000
H	-2.811434000	-0.283310000	-2.718717000
H	-3.084438000	0.676566000	-1.283606000
N	-1.078007000	0.793332000	-2.001649000
C	-0.480182000	0.481253000	-3.411580000
C	-1.366853000	1.106478000	-4.510244000
H	-2.416470000	0.808439000	-4.430265000
H	-1.001892000	0.757439000	-5.480375000
H	-1.309957000	2.198061000	-4.510526000
C	0.943836000	1.049978000	-3.516400000
H	0.980995000	2.127061000	-3.339798000
H	1.303163000	0.868585000	-4.534201000
H	1.609584000	0.555886000	-2.810431000
C	-0.424553000	-1.046802000	-3.584886000
H	0.070685000	-1.503094000	-2.728967000
H	0.161244000	-1.271833000	-4.480491000
H	-1.410727000	-1.497600000	-3.719523000
C	-1.385426000	2.269334000	-1.824593000
H	-2.223165000	2.565103000	-2.460412000
H	-0.506334000	2.835167000	-2.133575000
C	-1.703790000	2.535663000	-0.374571000
C	-2.525274000	3.544440000	0.121112000
H	-3.013250000	4.236883000	-0.552066000
C	-2.705975000	3.634271000	1.509498000
H	-3.330700000	4.418122000	1.919403000
C	-2.103348000	2.706949000	2.368428000
H	-2.259053000	2.755178000	3.438190000
C	-1.306892000	1.703920000	1.813726000
N	-1.114963000	1.671302000	0.482038000
C	-0.712279000	0.547945000	2.567548000
H	-0.427910000	0.837905000	3.577708000
H	-1.493340000	-0.211332000	2.672845000
O	1.356224000	1.761080000	-0.544157000
H	1.005466000	2.599363000	-0.212854000
O	1.347009000	-0.822405000	-0.906680000
I	2.576866000	-2.572802000	-1.447461000
C	1.208102000	-4.066018000	-0.914966000
C	0.191532000	-4.409210000	-1.813243000
H	0.099610000	-3.912797000	-2.771219000
C	-0.704366000	-5.412956000	-1.447198000
H	-1.501048000	-5.688106000	-2.129889000
C	-0.577111000	-6.059443000	-0.215096000
H	-1.278383000	-6.840085000	0.059436000
C	0.445992000	-5.702936000	0.666773000
H	0.543808000	-6.203687000	1.624017000
C	1.350761000	-4.697374000	0.326517000
H	2.148301000	-4.421488000	1.006485000

$^{3}TS_{12}$

Mn	-0.209835000	0.258143000	0.006479000
N	-0.156794000	0.116020000	2.186771000
C	1.213932000	0.244178000	2.931281000
C	1.700494000	1.700508000	2.818602000
H	1.737741000	2.014699000	1.774548000
H	1.089253000	2.404461000	3.387364000
H	2.715692000	1.752599000	3.222303000
C	1.049644000	-0.155048000	4.415485000
H	0.885468000	-1.229498000	4.531077000
H	0.241784000	0.379375000	4.922384000
H	1.979090000	0.092638000	4.935842000
C	2.262490000	-0.665401000	2.283008000
H	3.149224000	-0.679604000	2.923710000
H	2.548801000	-0.269580000	1.312390000
H	1.922521000	-1.696595000	2.169422000
C	-0.796024000	-1.246524000	2.404303000
H	-1.058352000	-1.387587000	3.454922000
H	-0.069531000	-2.005656000	2.115380000
C	-2.017688000	-1.343232000	1.532925000
C	-3.161410000	-2.092228000	1.791352000
H	-3.228470000	-2.697246000	2.685923000
C	-4.219113000	-2.036470000	0.871816000
H	-5.113803000	-2.621638000	1.044439000
C	-4.133690000	-1.210351000	-0.254401000
H	-4.953785000	-1.137070000	-0.956666000
C	-2.970121000	-0.464528000	-0.451024000
N	-1.951923000	-0.576211000	0.421503000
C	-2.773511000	0.563016000	-1.523625000
H	-3.319189000	0.293288000	-2.424576000
H	-3.204618000	1.505144000	-1.171979000
N	-1.300854000	0.807544000	-1.847154000
C	-0.918845000	0.115576000	-3.198670000
C	-1.803340000	0.623678000	-4.362877000
H	-2.849644000	0.319778000	-4.281920000
H	-1.412255000	0.181049000	-5.284067000
H	-1.757804000	1.709385000	-4.479438000
C	0.545408000	0.455773000	-3.540232000
H	0.619147000	1.410839000	-4.069475000
H	0.946181000	-0.315883000	-4.202710000
H	1.167510000	0.510041000	-2.649607000
C	-1.128706000	-1.397932000	-3.031442000

H	-0.553675000	-1.790342000	-2.194166000
H	-0.810710000	-1.898527000	-3.950407000
H	-2.182329000	-1.647064000	-2.874313000
C	-1.049262000	2.303724000	-1.916383000
H	-1.718145000	2.777586000	-2.638695000
H	-0.024878000	2.452682000	-2.256233000
C	-1.245367000	2.897694000	-0.551097000
C	-1.689228000	4.187187000	-0.271741000
H	-1.901683000	4.878278000	-1.076755000
C	-1.867225000	4.557630000	1.068512000
H	-2.199722000	5.559173000	1.311467000
C	-1.650775000	3.627364000	2.091730000
H	-1.823482000	3.886392000	3.128210000
C	-1.226197000	2.343810000	1.749119000
N	-0.995345000	2.032424000	0.457696000
C	-1.114977000	1.188977000	2.695672000
H	-0.828525000	1.515453000	3.693241000
H	-2.108231000	0.741393000	2.790071000
O	1.435993000	0.903010000	-0.456163000
H	1.613501000	1.852731000	-0.486420000
O	0.388373000	-1.366133000	-0.267403000
I	2.325811000	-1.636986000	-1.136201000
C	1.860293000	-3.674668000	-1.256677000
C	1.394827000	-4.192520000	-2.469525000
H	1.306660000	-3.562758000	-3.346911000
C	1.038461000	-5.540475000	-2.524248000
H	0.675323000	-5.958281000	-3.457338000
C	1.143944000	-6.343625000	-1.386224000
H	0.862268000	-7.390176000	-1.436558000
C	1.611412000	-5.807322000	-0.183469000
H	1.693926000	-6.433109000	0.698966000
C	1.978095000	-4.463577000	-0.107868000
H	2.342922000	-4.043270000	0.822079000

^{5}2'

Mn	-0.397646000	0.353636000	-0.442636000
N	-0.278333000	-0.541091000	1.668622000
C	1.149287000	-0.911791000	2.165569000
C	1.941909000	0.391155000	2.372306000
H	1.963588000	0.975322000	1.450486000
H	1.543842000	1.005064000	3.184182000
H	2.968853000	0.127179000	2.640589000
C	1.052972000	-1.705834000	3.487550000
H	0.586786000	-2.683976000	3.340529000
H	0.507947000	-1.170335000	4.269716000
H	2.068500000	-1.877340000	3.855686000
C	1.861674000	-1.781469000	1.119378000
H	2.880296000	-1.969169000	1.470961000
H	1.913449000	-1.290694000	0.149180000
H	1.382399000	-2.752070000	0.992077000
C	-1.184645000	-1.742563000	1.514011000
H	-1.445467000	-2.157899000	2.491123000
H	-0.636137000	-2.502102000	0.959672000
C	-2.430637000	-1.344747000	0.764966000
C	-3.679337000	-1.944143000	0.902463000
H	-3.815098000	-2.769377000	1.588841000
C	-4.748065000	-1.451917000	0.140867000
H	-5.723536000	-1.915306000	0.219692000
C	-4.568937000	-0.344984000	-0.696695000
H	-5.395878000	0.067656000	-1.259533000
C	-3.303862000	0.237811000	-0.779029000
N	-2.274892000	-0.302389000	-0.087814000
C	-3.011161000	1.532841000	-1.486190000
H	-3.655485000	1.663744000	-2.354218000
H	-3.256858000	2.343016000	-0.793090000
N	-1.549792000	1.689904000	-1.883928000
C	-1.330662000	1.431593000	-3.403319000
C	-1.886861000	2.620557000	-4.217787000
H	-2.922766000	2.858953000	-3.957840000
H	-1.868064000	2.348092000	-5.276682000
H	-1.277048000	3.519455000	-4.096945000
C	0.168327000	1.253065000	-3.694281000
H	0.774159000	2.084143000	-3.327731000
H	0.300418000	1.200935000	-4.779096000
H	0.531623000	0.321978000	-3.257103000
C	-2.065079000	0.138749000	-3.793789000
H	-1.786898000	-0.664729000	-3.113308000
H	-1.753884000	-0.147230000	-4.801954000
H	-3.151971000	0.244253000	-3.804223000
C	-1.054093000	3.058485000	-1.457026000
H	-1.680525000	3.840927000	-1.891441000
H	-0.037378000	3.174788000	-1.832648000
C	-1.056133000	3.156858000	0.045572000
C	-1.210924000	4.333335000	0.774726000
H	-1.347533000	5.278530000	0.265829000
C	-1.191684000	4.260642000	2.173911000
H	-1.296657000	5.163983000	2.761843000
C	-1.062142000	3.023884000	2.816006000
H	-1.073195000	2.951902000	3.895776000
C	-0.934473000	1.873933000	2.036532000
N	-0.904495000	1.977141000	0.691035000
C	-0.944582000	0.475188000	2.584043000
H	-0.492852000	0.441528000	3.574417000
H	-1.989871000	0.177276000	2.710917000
O	1.203639000	1.123041000	-0.695786000
H	1.766103000	0.590534000	-1.280160000
O	-0.030767000	-1.070544000	-1.374752000
I	-1.071804000	-3.169800000	-2.155206000
C	0.303699000	-4.261880000	-1.018325000
C	-0.143296000	-4.932113000	0.126384000
H	-1.190385000	-4.923755000	0.405928000
C	0.793693000	-5.613698000	0.904153000
H	0.464842000	-6.135613000	1.796356000
C	2.142358000	-5.620199000	0.539309000
H	2.864617000	-6.147109000	1.153541000
C	2.564935000	-4.950515000	-0.612281000
H	3.612127000	-4.953729000	-0.894896000
C	1.646993000	-4.264503000	-1.406968000
H	1.970666000	-3.735290000	-2.294939000

^{3}2'

Mn	0.018752000	0.192153000	-0.369348000
N	0.189986000	-0.371547000	1.726171000
C	1.594133000	-0.187365000	2.397324000
C	1.851769000	1.311655000	2.630996000
H	1.774302000	1.874924000	1.701964000
H	1.187804000	1.752369000	3.377870000
H	2.874326000	1.422896000	3.002210000

C	1.625426000	-0.940490000	3.746175000
H	1.613147000	-2.024550000	3.609079000
H	0.804363000	-0.659475000	4.412086000
H	2.559500000	-0.684180000	4.253640000
C	2.685786000	-0.747456000	1.476984000
H	3.642936000	-0.675784000	2.002531000
H	2.750429000	-0.173692000	0.554366000
H	2.523941000	-1.798151000	1.235661000
C	-0.231870000	-1.833236000	1.692396000
H	-0.426487000	-2.197506000	2.702924000
H	0.582515000	-2.414215000	1.260298000
C	-1.457728000	-1.954799000	0.832954000
C	-2.440784000	-2.935308000	0.923180000
H	-2.373328000	-3.709528000	1.675472000
C	-3.508005000	-2.896676000	0.014791000
H	-4.276482000	-3.658357000	0.056559000
C	-3.594726000	-1.873802000	-0.937580000
H	-4.422518000	-1.826943000	-1.632587000
C	-2.592925000	-0.904010000	-0.969200000
N	-1.565397000	-0.986335000	-0.103088000
C	-2.583854000	0.318368000	-1.833686000
H	-3.058856000	0.132044000	-2.794521000
H	-3.179296000	1.088941000	-1.335755000
N	-1.172708000	0.874818000	-2.042147000
C	-0.636862000	0.510577000	-3.471567000
C	-1.436295000	1.285590000	-4.542882000
H	-2.516434000	1.133491000	-4.466988000
H	-1.124805000	0.916574000	-5.524131000
H	-1.229217000	2.358340000	-4.515179000
C	0.847775000	0.887409000	-3.587395000
H	1.022627000	1.957265000	-3.452911000
H	1.181540000	0.624753000	-4.595687000
H	1.453955000	0.344206000	-2.867244000
C	-0.801407000	-1.004908000	-3.684036000
H	-0.333450000	-1.560553000	-2.871821000
H	-0.300600000	-1.274163000	-4.618108000
H	-1.846970000	-1.307710000	-3.779620000
C	-1.226609000	2.379824000	-1.826427000
H	-2.001133000	2.825940000	-2.453683000
H	-0.264502000	2.797700000	-2.122986000
C	-1.499332000	2.650750000	-0.370783000
C	-2.139507000	3.765108000	0.162821000
H	-2.500801000	4.553433000	-0.484054000
C	-2.308514000	3.833887000	1.553846000
H	-2.793259000	4.696778000	1.993105000
C	-1.877371000	2.786266000	2.377111000
H	-2.028175000	2.819400000	3.447992000
C	-1.260718000	1.684242000	1.785160000
N	-1.067470000	1.667787000	0.452110000
C	-0.878970000	0.414481000	2.484614000
H	-0.558614000	0.597685000	3.508444000
H	-1.774733000	-0.210102000	2.546610000
O	1.384656000	1.327279000	-0.607819000
H	1.237347000	2.221821000	-0.269329000
O	0.728720000	-1.213746000	-0.963064000
I	2.937371000	-2.210060000	-2.015452000
C	1.985955000	-3.730139000	-0.917351000
C	0.700169000	-4.124635000	-1.301581000
H	0.241574000	-3.725539000	-2.197024000
C	0.009904000	-5.030778000	-0.495391000
H	-0.991619000	-5.336712000	-0.778360000
C	0.597972000	-5.531364000	0.668397000
H	0.051370000	-6.228438000	1.294622000
C	1.891067000	-5.139341000	1.025578000
H	2.352445000	-5.530605000	1.926214000
C	2.599397000	-4.233679000	0.232818000
H	3.597069000	-3.919286000	0.515556000

^{5}**2**

Mn	-0.814529000	-0.067136000	-0.056344000
N	-0.843855000	-0.460197000	2.060241000
C	0.452182000	-1.082965000	2.695062000
C	1.577367000	-0.035090000	2.637282000
H	1.750529000	0.299978000	1.613987000
H	1.384314000	0.828563000	3.278021000
H	2.495406000	-0.505748000	3.000253000
C	0.169220000	-1.484749000	4.160691000
H	-0.534211000	-2.318171000	4.229158000
H	-0.201597000	-0.659031000	4.773037000
H	1.113258000	-1.815654000	4.602206000
C	0.877701000	-2.335953000	1.916373000
H	1.759519000	-2.753482000	2.411031000
H	1.145805000	-2.090947000	0.893389000
H	0.110974000	-3.113811000	1.910236000
C	-2.040654000	-1.389530000	2.207191000
H	-2.351061000	-1.448878000	3.252131000
H	-1.738326000	-2.382565000	1.876573000
C	-3.157001000	-0.874227000	1.344242000
C	-4.521250000	-1.044064000	1.551006000
H	-4.879227000	-1.614578000	2.397255000
C	-5.414015000	-0.449350000	0.647155000
H	-6.480099000	-0.580640000	0.782458000
C	-4.941961000	0.339474000	-0.410999000
H	-5.626765000	0.834251000	-1.086455000
C	-3.567226000	0.493221000	-0.565762000
N	-2.731624000	-0.142199000	0.286212000
C	-2.881719000	1.421649000	-1.520160000
H	-3.434116000	1.529981000	-2.450951000
H	-2.849406000	2.411924000	-1.056087000
N	-1.443185000	0.984368000	-1.802805000
C	-1.366659000	0.233633000	-3.184652000
C	-1.591394000	1.247935000	-4.328849000
H	-2.502644000	1.839944000	-4.210006000
H	-1.691238000	0.682165000	-5.259148000
H	-0.743039000	1.926590000	-4.447268000
C	0.006249000	-0.424274000	-3.364254000
H	0.840540000	0.262091000	-3.209074000
H	0.071124000	-0.785859000	-4.393447000
H	0.109947000	-1.289645000	-2.713181000
C	-2.448389000	-0.860505000	-3.222020000
H	-2.357045000	-1.533675000	-2.368260000
H	-2.293984000	-1.451599000	-4.129438000
H	-3.462868000	-0.457970000	-3.266348000
C	-0.540946000	2.213287000	-1.780585000
H	-0.921557000	2.974767000	-2.463271000
H	0.449540000	1.905624000	-2.116378000
C	-0.471570000	2.736581000	-0.372999000
C	-0.238057000	4.048960000	0.023629000
H	-0.072302000	4.824396000	-0.712058000

C	-0.233937000	4.339439000	1.396524000
H	-0.044930000	5.352652000	1.728301000
C	-0.496454000	3.338751000	2.341332000
H	-0.522051000	3.561958000	3.399531000
C	-0.740626000	2.044064000	1.887735000
N	-0.689831000	1.787198000	0.564933000
C	-1.176091000	0.879395000	2.724100000
H	-0.751119000	0.920779000	3.724713000
H	-2.262231000	0.936151000	2.842763000
O	0.908598000	-0.180013000	-0.466616000
H	1.230120000	-0.898073000	-1.037252000
O	-1.002741000	-1.770646000	-0.549524000
I	-0.103123000	-3.992054000	-5.581541000
C	1.008601000	-3.632788000	-3.813104000
C	0.498810000	-4.067257000	-2.589005000
H	-0.437016000	-4.610665000	-2.538316000
C	1.210349000	-3.781534000	-1.421062000
H	0.820969000	-4.122485000	-0.468655000
C	2.408627000	-3.063351000	-1.478932000
H	2.963946000	-2.854813000	-0.570131000
C	2.902028000	-2.633835000	-2.714220000
H	3.832714000	-2.078344000	-2.767479000
C	2.207096000	-2.920549000	-3.891946000
H	2.588858000	-2.581850000	-4.847763000

^{3}2

Mn	-0.645010000	-0.038705000	-0.051800000
N	-0.628144000	-0.360096000	2.082907000
C	0.721080000	-0.877940000	2.701554000
C	1.744466000	0.269968000	2.726385000
H	1.906668000	0.682442000	1.732175000
H	1.470497000	1.074513000	3.411928000
H	2.697298000	-0.138728000	3.073736000
C	0.465811000	-1.375700000	4.142289000
H	-0.126459000	-2.293462000	4.162391000
H	-0.020637000	-0.625670000	4.772243000
H	1.435306000	-1.601425000	4.594536000
C	1.271052000	-2.032508000	1.851762000
H	2.166167000	-2.418969000	2.348141000
H	1.551168000	-1.687718000	0.857231000
H	0.564338000	-2.860427000	1.762474000
C	-1.748869000	-1.374784000	2.263863000
H	-2.021390000	-1.451995000	3.317774000
H	-1.386336000	-2.344854000	1.923859000
C	-2.922563000	-0.941293000	1.433511000
C	-4.263808000	-1.213942000	1.679705000
H	-4.554269000	-1.789178000	2.548494000
C	-5.222093000	-0.726618000	0.778924000
H	-6.270869000	-0.938442000	0.944265000
C	-4.835075000	0.045527000	-0.322585000
H	-5.567429000	0.443525000	-1.012135000
C	-3.479125000	0.307107000	-0.512763000
N	-2.580001000	-0.204064000	0.351418000
C	-2.904036000	1.214736000	-1.552403000
H	-3.465198000	1.166146000	-2.483104000
H	-2.992059000	2.242553000	-1.188242000
N	-1.424133000	0.930499000	-1.813471000
C	-1.232433000	0.150911000	-3.165210000
C	-1.560637000	1.088968000	-4.348153000
H	-2.554154000	1.540611000	-4.276082000
H	-1.541844000	0.489031000	-5.262233000
H	-0.818510000	1.882922000	-4.461968000
C	0.218207000	-0.332468000	-3.290382000
H	0.944538000	0.479505000	-3.216147000
H	0.332134000	-0.784813000	-4.278448000
H	0.456211000	-1.086533000	-2.543453000
C	-2.178145000	-1.063027000	-3.180375000
H	-2.018122000	-1.701579000	-2.311793000
H	-1.950229000	-1.652635000	-4.072542000
H	-3.231784000	-0.780592000	-3.240660000
C	-0.691053000	2.264050000	-1.817756000
H	-1.173584000	2.958194000	-2.508375000
H	0.327186000	2.090918000	-2.165675000
C	-0.691718000	2.816569000	-0.417773000
C	-0.632015000	4.156484000	-0.050063000
H	-0.584594000	4.930711000	-0.804075000
C	-0.639614000	4.474281000	1.316584000
H	-0.582497000	5.510297000	1.625952000
C	-0.739755000	3.466108000	2.283278000
H	-0.768279000	3.704897000	3.338078000
C	-0.816770000	2.140430000	1.858192000
N	-0.764895000	1.862521000	0.540114000
C	-1.066712000	0.952332000	2.736935000
H	-0.592208000	1.062356000	3.709961000
H	-2.143199000	0.892846000	2.921976000
O	1.079496000	0.213131000	-0.432614000
H	1.435247000	1.093057000	-0.242123000
O	-0.753143000	-1.672405000	-0.458861000
I	0.678870000	-3.437501000	-6.246350000
C	0.955278000	-3.591966000	-4.150753000
C	-0.103278000	-4.036130000	-3.357015000
H	-1.046749000	-4.327859000	-3.802628000
C	0.069328000	-4.088419000	-1.973351000
H	-0.749122000	-4.424220000	-1.346206000
C	1.282833000	-3.702796000	-1.395727000
H	1.412231000	-3.759634000	-0.322373000
C	2.332975000	-3.264540000	-2.206784000
H	3.278201000	-2.967619000	-1.763713000
C	2.177499000	-3.206783000	-3.592453000
H	2.989181000	-2.861090000	-4.221611000

B. Thioanisole sulfoxidation by complex 1 via the “1e+1e” electron transfer mechanism

^{5}RC

Mn	-0.501374000	-0.223673000	-0.257421000
N	-0.690189000	-0.753622000	2.051794000
C	0.539351000	-1.380330000	2.735048000
C	1.638596000	-0.305136000	2.841579000
H	1.861607000	0.108566000	1.855404000
H	1.380220000	0.508851000	3.523158000
H	2.549074000	-0.775029000	3.224916000
C	0.173460000	-1.919812000	4.133770000
H	-0.503998000	-2.776450000	4.069798000
H	-0.288503000	-1.157430000	4.769029000
H	1.086012000	-2.257500000	4.634133000
C	1.085263000	-2.517195000	1.862998000
H	1.935359000	-2.979266000	2.374207000
H	1.438127000	-2.113983000	0.917782000
H	0.355137000	-3.305195000	1.669824000
C	-1.858597000	-1.694434000	1.954531000
H	-2.280073000	-1.929138000	2.937137000
H	-1.511147000	-2.625235000	1.505015000
C	-2.928099000	-1.085904000	1.086105000
C	-4.294024000	-1.258988000	1.296546000
H	-4.637831000	-1.886111000	2.107047000
C	-5.196891000	-0.612091000	0.445796000
H	-6.262631000	-0.750325000	0.579653000
C	-4.721537000	0.243588000	-0.552645000
H	-5.404774000	0.791249000	-1.188945000
C	-3.344684000	0.415438000	-0.701791000
N	-2.490038000	-0.290057000	0.077292000
C	-2.761297000	1.477959000	-1.596730000
H	-3.400688000	1.617680000	-2.469085000
H	-2.807673000	2.421335000	-1.040451000
N	-1.337228000	1.227486000	-1.995245000
C	-1.219971000	0.731322000	-3.446923000
C	-1.747708000	1.778454000	-4.453554000
H	-2.814271000	1.986840000	-4.331321000
H	-1.606198000	1.390063000	-5.467058000
H	-1.196701000	2.720767000	-4.380962000
C	0.261933000	0.450940000	-3.771487000
H	0.799839000	1.371171000	-4.020110000
H	0.318337000	-0.202553000	-4.646715000
H	0.778463000	-0.028602000	-2.939667000
C	-2.050740000	-0.560511000	-3.559385000
H	-1.763718000	-1.280442000	-2.791295000
H	-1.891448000	-1.010204000	-4.543399000
H	-3.122837000	-0.370595000	-3.455843000
C	-0.478449000	2.423545000	-1.714781000
H	-0.829871000	3.315923000	-2.244327000
H	0.528659000	2.200907000	-2.070186000
C	-0.445923000	2.707886000	-0.233667000
C	-0.347122000	3.987282000	0.307769000
H	-0.260778000	4.845684000	-0.345733000
C	-0.379050000	4.136705000	1.699465000
H	-0.288322000	5.121318000	2.141282000
C	-0.570782000	3.018249000	2.515358000
H	-0.651613000	3.118767000	3.590111000
C	-0.686994000	1.758723000	1.924733000
N	-0.565212000	1.630937000	0.583388000
C	-1.097718000	0.533128000	2.702029000
H	-0.732631000	0.599204000	3.727878000
H	-2.190769000	0.557922000	2.772401000
O	1.263135000	-0.123650000	-0.653496000
H	1.731140000	0.672616000	-0.368513000
O	-0.600629000	-2.079497000	-0.852852000
I	0.613739000	-2.945200000	-2.065962000
C	2.459198000	-3.205244000	-1.094345000
C	2.682657000	-4.404565000	-0.413005000
H	1.912130000	-5.160300000	-0.342793000
C	3.919649000	-4.585307000	0.204547000
H	4.114744000	-5.504835000	0.745874000
C	4.893290000	-3.586091000	0.131835000
H	5.851610000	-3.732589000	0.618970000
C	4.644319000	-2.397059000	-0.561039000
H	5.404846000	-1.625319000	-0.612933000
C	3.416418000	-2.189930000	-1.186708000
H	3.193487000	-1.261922000	-1.694840000
C	-0.184333000	-6.619231000	-0.253734000
C	-1.380874000	-5.922744000	-0.456672000
C	-2.057868000	-5.356232000	0.636331000
C	-1.513695000	-5.484959000	1.928480000
C	-0.318287000	-6.183577000	2.123416000
C	0.350215000	-6.756227000	1.034237000
H	0.322947000	-7.063893000	-1.104039000
H	-1.787773000	-5.841120000	-1.457149000
H	-2.026003000	-5.038390000	2.774206000
H	0.091434000	-6.273756000	3.123600000
H	1.275521000	-7.301090000	1.186407000
S	-3.635245000	-4.532696000	0.481759000
C	-3.517772000	-3.744137000	-1.166379000
H	-3.549755000	-4.478982000	-1.972513000
H	-4.386393000	-3.087579000	-1.253865000
H	-2.605006000	-3.148287000	-1.225231000

^{3}RC

Mn	-0.594727000	-0.132501000	-0.173061000
N	-0.780418000	-0.779138000	1.897110000
C	0.452425000	-1.439893000	2.591486000
C	1.533581000	-0.364284000	2.806565000
H	1.786375000	0.114818000	1.859745000
H	1.247740000	0.396635000	3.536508000
H	2.432974000	-0.857240000	3.187220000
C	0.035867000	-2.060544000	3.943187000
H	-0.587648000	-2.947557000	3.805204000
H	-0.490340000	-1.358811000	4.596940000
H	0.942997000	-2.376798000	4.466235000
C	1.039613000	-2.531652000	1.692144000
H	1.839125000	-3.039086000	2.241074000
H	1.464421000	-2.078507000	0.802294000
H	0.309449000	-3.288544000	1.403838000
C	-1.959789000	-1.728668000	1.830699000
H	-2.343441000	-1.954040000	2.828345000
H	-1.618311000	-2.654522000	1.370421000
C	-3.031175000	-1.099902000	0.986002000
C	-4.403296000	-1.294979000	1.120285000
H	-4.785015000	-1.966775000	1.875916000
C	-5.265375000	-0.608830000	0.254518000
H	-6.335337000	-0.758825000	0.329164000
C	-4.752288000	0.291230000	-0.687154000
H	-5.409560000	0.851550000	-1.339289000
C	-3.371299000	0.475496000	-0.752595000
N	-2.559949000	-0.244916000	0.049355000
C	-2.686218000	1.528268000	-1.571634000
H	-3.231804000	1.721361000	-2.493521000
H	-2.713048000	2.461267000	-0.999233000
N	-1.230527000	1.192680000	-1.862460000
C	-1.068850000	0.690815000	-3.324761000
C	-1.499203000	1.762305000	-4.353236000
H	-2.558536000	2.023854000	-4.291271000
H	-1.326316000	1.351818000	-5.353094000

H	-0.905243000	2.676118000	-4.268396000
C	0.417495000	0.365146000	-3.576175000
H	0.977591000	1.263270000	-3.855166000
H	0.498548000	-0.338111000	-4.409145000
H	0.888300000	-0.067163000	-2.694430000
C	-1.967793000	-0.549633000	-3.481251000
H	-1.832657000	-1.246098000	-2.653536000
H	-1.726870000	-1.056254000	-4.419725000
H	-3.026959000	-0.279572000	-3.519847000
C	-0.378372000	2.416288000	-1.603016000
H	-0.737905000	3.278636000	-2.171088000
H	0.635531000	2.193358000	-1.936282000
C	-0.390451000	2.721047000	-0.130928000
C	-0.249201000	3.980538000	0.444923000
H	-0.092129000	4.851676000	-0.177518000
C	-0.328663000	4.091426000	1.840075000
H	-0.209089000	5.059254000	2.311123000
C	-0.595437000	2.963288000	2.624703000
H	-0.698654000	3.043292000	3.698987000
C	-0.747679000	1.729095000	1.993236000
N	-0.597355000	1.637777000	0.654022000
C	-1.202014000	0.467068000	2.663235000
H	-0.865968000	0.421230000	3.697812000
H	-2.295711000	0.491856000	2.697164000
O	1.195225000	-0.132601000	-0.489960000
H	1.670435000	0.688264000	-0.306135000
O	-0.683095000	-2.025394000	-0.758665000
I	0.413443000	-2.923883000	-2.057778000
C	2.365996000	-3.130672000	-1.284152000
C	2.686428000	-4.306096000	-0.601514000
H	1.938645000	-5.065165000	-0.416545000
C	3.991222000	-4.460264000	-0.132793000
H	4.260073000	-5.362678000	0.405897000
C	4.938146000	-3.456894000	-0.350043000
H	5.950157000	-3.582149000	0.020607000
C	4.592162000	-2.290573000	-1.039338000
H	5.331295000	-1.513900000	-1.204783000
C	3.294436000	-2.112589000	-1.516765000
H	3.007643000	-1.201541000	-2.023624000
C	-0.076397000	-6.503406000	0.110073000
C	-1.312534000	-5.913036000	-0.175756000
C	-2.082796000	-5.359620000	0.860200000
C	-1.595497000	-5.400879000	2.180660000
C	-0.358745000	-5.991471000	2.457817000
C	0.407299000	-6.544005000	1.423915000
H	0.499442000	-6.945405000	-0.696947000
H	-1.675722000	-5.903892000	-1.196016000
H	-2.184028000	-4.970871000	2.984334000
H	0.007288000	-6.011246000	3.478581000
H	1.366746000	-7.001347000	1.638672000
S	-3.703992000	-4.655858000	0.593881000
C	-3.545283000	-3.909317000	-1.071198000
H	-3.477314000	-4.667916000	-1.852726000
H	-4.450469000	-3.318966000	-1.229760000
H	-2.675442000	-3.249970000	-1.100018000

^{5}TS1

Mn	-0.587205000	-0.282323000	-0.226400000
N	-0.809967000	-0.880528000	1.929985000
C	0.400824000	-1.629649000	2.561562000
C	1.546964000	-0.627687000	2.787547000
H	1.810509000	-0.125150000	1.856570000
H	1.323950000	0.118705000	3.553345000
H	2.423752000	-1.187454000	3.125152000
C	-0.024128000	-2.264849000	3.903806000
H	-0.717968000	-3.095405000	3.756992000
H	-0.473325000	-1.542900000	4.592230000
H	0.870813000	-2.666670000	4.387177000
C	0.888633000	-2.719977000	1.600805000
H	1.669193000	-3.297661000	2.103570000
H	1.315861000	-2.265567000	0.710752000
H	0.104409000	-3.418339000	1.307516000
C	-2.046869000	-1.753774000	1.849238000
H	-2.441505000	-1.953613000	2.848078000
H	-1.760645000	-2.701574000	1.397112000
C	-3.083168000	-1.069193000	1.002523000
C	-4.460412000	-1.215261000	1.140456000
H	-4.862518000	-1.868065000	1.902323000
C	-5.299911000	-0.507667000	0.270686000
H	-6.374115000	-0.620854000	0.346782000
C	-4.756467000	0.366545000	-0.677323000
H	-5.395085000	0.945058000	-1.332102000
C	-3.370158000	0.503532000	-0.751789000
N	-2.581338000	-0.240610000	0.054656000
C	-2.677025000	1.538073000	-1.592021000
H	-3.233194000	1.721050000	-2.510520000
H	-2.691725000	2.478942000	-1.032232000
N	-1.232264000	1.201540000	-1.905616000
C	-1.060757000	0.700658000	-3.364687000
C	-1.412244000	1.820430000	-4.370479000
H	-2.446945000	2.162907000	-4.284633000
H	-1.285370000	1.418471000	-5.380161000
H	-0.747051000	2.682636000	-4.273929000
C	0.402521000	0.279121000	-3.586977000
H	1.065132000	1.142462000	-3.699240000
H	0.462788000	-0.298585000	-4.513293000
H	0.768344000	-0.333263000	-2.765489000
C	-2.001775000	-0.500046000	-3.566989000
H	-1.821605000	-1.262554000	-2.808291000
H	-1.812878000	-0.930412000	-4.554437000
H	-3.055633000	-0.210003000	-3.535494000
C	-0.348726000	2.387432000	-1.591554000
H	-0.678922000	3.273662000	-2.140506000
H	0.661997000	2.143665000	-1.919700000
C	-0.361612000	2.661810000	-0.109345000
C	-0.213531000	3.915551000	0.478118000
H	-0.068780000	4.793888000	-0.137200000
C	-0.266807000	4.011003000	1.875577000
H	-0.139445000	4.973693000	2.354852000
C	-0.517279000	2.874355000	2.652604000
H	-0.601477000	2.943594000	3.729407000
C	-0.682373000	1.646610000	2.010099000
N	-0.555784000	1.569843000	0.668089000
C	-1.143629000	0.389897000	2.695523000
H	-0.756040000	0.329139000	3.711439000
H	-2.232683000	0.451241000	2.784537000
O	1.189607000	-0.193231000	-0.491017000
H	1.622802000	0.606179000	-0.158636000
O	-0.791435000	-1.864527000	-0.942799000
I	0.571168000	-3.334239000	-2.244132000
C	2.509448000	-3.182595000	-1.483397000

C	2.956483000	-4.151171000	-0.575703000
H	2.310482000	-4.963550000	-0.267642000
C	4.244278000	-4.030791000	-0.058070000
H	4.606852000	-4.773028000	0.645130000
C	5.058250000	-2.958062000	-0.434666000
H	6.057882000	-2.868944000	-0.022555000
C	4.590745000	-1.998491000	-1.337332000
H	5.223665000	-1.166488000	-1.627507000
C	3.307407000	-2.099725000	-1.871556000
H	2.935544000	-1.354006000	-2.561847000
C	-0.049508000	-6.326637000	0.362989000
C	-1.279660000	-5.772445000	-0.010063000
C	-2.174608000	-5.320457000	0.972609000
C	-1.822525000	-5.437044000	2.332027000
C	-0.595285000	-5.998293000	2.696003000
C	0.299496000	-6.441896000	1.713141000
H	0.627914000	-6.679460000	-0.408031000
H	-1.536321000	-5.704097000	-1.059273000
H	-2.506209000	-5.083211000	3.096853000
H	-0.334516000	-6.076955000	3.745862000
H	1.254234000	-6.870583000	1.996393000
S	-3.777720000	-4.630073000	0.587787000
C	-3.515865000	-3.899310000	-1.071131000
H	-3.414609000	-4.667331000	-1.840335000
H	-4.407061000	-3.304102000	-1.283388000
H	-2.640110000	-3.245038000	-1.066571000

^{3}TS1

Mn	-0.400047000	-0.229177000	-0.262085000
N	-0.508974000	-0.909849000	1.905140000
C	0.807438000	-1.434414000	2.543578000
C	1.760847000	-0.244364000	2.757157000
H	1.921876000	0.288518000	1.817966000
H	1.415205000	0.457474000	3.519469000
H	2.725631000	-0.637360000	3.090475000
C	0.495822000	-2.132695000	3.884898000
H	-0.046679000	-3.069681000	3.734150000
H	-0.075860000	-1.500803000	4.571311000
H	1.443777000	-2.374736000	4.373892000
C	1.483457000	-2.420752000	1.587681000
H	2.352217000	-2.857351000	2.089725000
H	1.825915000	-1.895305000	0.701268000
H	0.828451000	-3.238135000	1.289877000
C	-1.580787000	-1.975364000	1.801368000
H	-1.931107000	-2.269261000	2.793431000
H	-1.144145000	-2.846105000	1.314763000
C	-2.725685000	-1.445257000	0.978280000
C	-4.059199000	-1.807828000	1.133312000
H	-4.340030000	-2.537022000	1.880186000
C	-5.018955000	-1.215658000	0.301828000
H	-6.059187000	-1.503003000	0.389586000
C	-4.639682000	-0.239681000	-0.623478000
H	-5.373607000	0.251705000	-1.248986000
C	-3.291354000	0.113477000	-0.718590000
N	-2.377197000	-0.521879000	0.045399000
C	-2.796746000	1.271144000	-1.542519000
H	-3.410147000	1.390852000	-2.434765000
H	-2.941796000	2.180007000	-0.949382000
N	-1.330158000	1.182249000	-1.915003000
C	-1.135025000	0.771514000	-3.394362000
C	-1.728650000	1.829017000	-4.352183000
H	-2.808273000	1.955202000	-4.235524000
H	-1.549506000	1.494109000	-5.378488000
H	-1.245443000	2.802795000	-4.235328000
C	0.373334000	0.641778000	-3.683590000
H	0.830071000	1.618015000	-3.872869000
H	0.513001000	0.040639000	-4.586235000
H	0.906422000	0.170837000	-2.857611000
C	-1.856549000	-0.572015000	-3.598434000
H	-1.558673000	-1.294334000	-2.838314000
H	-1.606912000	-0.969297000	-4.585809000
H	-2.944086000	-0.467200000	-3.554114000
C	-0.625948000	2.474122000	-1.578630000
H	-1.099064000	3.322187000	-2.082440000
H	0.399896000	2.398466000	-1.940599000
C	-0.638752000	2.691078000	-0.088450000
C	-0.680087000	3.938655000	0.528348000
H	-0.683459000	4.840210000	-0.070195000
C	-0.731539000	3.998123000	1.926746000
H	-0.747827000	4.958440000	2.426982000
C	-0.801128000	2.818902000	2.675073000
H	-0.888254000	2.846919000	3.753501000
C	-0.783572000	1.594587000	2.006345000
N	-0.650878000	1.558576000	0.659920000
C	-1.053963000	0.277248000	2.674761000
H	-0.681944000	0.271977000	3.698409000
H	-2.140616000	0.166653000	2.742109000
O	1.357716000	0.047087000	-0.620001000
H	1.703383000	0.921323000	-0.388434000
O	-0.384562000	-1.917357000	-0.879299000
I	0.890539000	-2.804028000	-2.309703000
C	2.710471000	-3.220892000	-1.354843000
C	2.932917000	-4.517170000	-0.880659000
H	2.189279000	-5.295067000	-1.004322000
C	4.145834000	-4.785435000	-0.245383000
H	4.340197000	-5.784371000	0.130062000
C	5.097203000	-3.774449000	-0.089784000
H	6.034343000	-3.990114000	0.412401000
C	4.848984000	-2.485742000	-0.572571000
H	5.588746000	-1.702564000	-0.444956000
C	3.646887000	-2.192342000	-1.213734000
H	3.416958000	-1.188573000	-1.547635000
C	-0.368512000	-5.538976000	1.137055000
C	-1.318270000	-5.273722000	0.144005000
C	-2.671142000	-5.108499000	0.490577000
C	-3.054421000	-5.217572000	1.840697000
C	-2.099189000	-5.485681000	2.826579000
C	-0.751256000	-5.643397000	2.480287000
H	0.671907000	-5.666812000	0.859818000
H	-1.003651000	-5.203678000	-0.889483000
H	-4.095416000	-5.086412000	2.116446000
H	-2.407957000	-5.561119000	3.863552000
H	-0.009732000	-5.846185000	3.244686000
S	-3.949559000	-4.793681000	-0.715974000
C	-3.047931000	-3.828504000	-1.984396000
H	-2.455140000	-4.471377000	-2.638524000
H	-3.802571000	-3.312554000	-2.581950000
H	-2.401736000	-3.092802000	-1.501624000

^{5}IM1

Mn	-0.571488000	-0.328235000	-0.277761000
N	-0.771350000	-0.894992000	1.884629000
C	0.439313000	-1.650259000	2.512501000
C	1.598842000	-0.658638000	2.713086000
H	1.856645000	-0.169295000	1.773472000
H	1.394426000	0.098394000	3.473561000
H	2.473651000	-1.224766000	3.045189000
C	0.020643000	-2.263157000	3.867037000
H	-0.680019000	-3.091003000	3.737538000
H	-0.417484000	-1.528533000	4.549164000

H	0.916980000	-2.664420000	4.348192000
C	0.904371000	-2.758393000	1.561413000
H	1.677760000	-3.343478000	2.066507000
H	1.335100000	-2.320467000	0.665009000
H	0.107967000	-3.446250000	1.277561000
C	-2.019695000	-1.755735000	1.833287000
H	-2.400626000	-1.938323000	2.840451000
H	-1.750192000	-2.711905000	1.388769000
C	-3.060093000	-1.071173000	0.991972000
C	-4.436891000	-1.201358000	1.147679000
H	-4.836754000	-1.838817000	1.923581000
C	-5.279358000	-0.498726000	0.276687000
H	-6.353653000	-0.600047000	0.366648000
C	-4.738404000	0.354492000	-0.691681000
H	-5.378818000	0.927812000	-1.349198000
C	-3.351844000	0.476778000	-0.784280000
N	-2.561170000	-0.261228000	0.026109000
C	-2.659148000	1.487501000	-1.653064000
H	-3.223285000	1.653507000	-2.569720000
H	-2.660960000	2.441007000	-1.114941000
N	-1.219147000	1.133676000	-1.975947000
C	-1.065114000	0.611593000	-3.431311000
C	-1.440817000	1.713243000	-4.448466000
H	-2.482226000	2.035409000	-4.366603000
H	-1.306374000	1.301684000	-5.453316000
H	-0.793338000	2.590012000	-4.363636000
C	0.398762000	0.200127000	-3.668442000
H	1.048184000	1.067629000	-3.819675000
H	0.448621000	-0.404653000	-4.577932000
H	0.787342000	-0.384497000	-2.837076000
C	-1.997541000	-0.600169000	-3.601202000
H	-1.784526000	-1.358773000	-2.846987000
H	-1.835223000	-1.029626000	-4.593772000
H	-3.053372000	-0.322712000	-3.536086000
C	-0.321479000	2.316292000	-1.687183000
H	-0.648040000	3.196588000	-2.247480000
H	0.683835000	2.057129000	-2.019964000
C	-0.317986000	2.612628000	-0.209676000
C	-0.153693000	3.873768000	0.357112000
H	-0.010058000	4.741468000	-0.273326000
C	-0.188663000	3.990811000	1.753391000
H	-0.048280000	4.959542000	2.216509000
C	-0.437281000	2.867810000	2.550534000
H	-0.506922000	2.953834000	3.627121000
C	-0.619866000	1.631868000	1.929177000
N	-0.511197000	1.533195000	0.586312000
C	-1.081212000	0.389697000	2.639332000
H	-0.676827000	0.337846000	3.649082000
H	-2.167983000	0.461790000	2.746015000
O	1.200995000	-0.269365000	-0.572719000
H	1.657001000	0.522885000	-0.253293000
O	-0.818037000	-1.915206000	-0.953732000
I	0.726814000	-3.390863000	-2.276348000
C	2.628399000	-3.237197000	-1.432066000
C	3.062956000	-4.234515000	-0.548889000
H	2.432144000	-5.082396000	-0.313839000
C	4.318136000	-4.100194000	0.039529000
H	4.669986000	-4.864724000	0.724090000
C	5.113986000	-2.984957000	-0.242123000
H	6.087751000	-2.885298000	0.225772000
C	4.660668000	-1.997489000	-1.121303000
H	5.278969000	-1.132827000	-1.338396000
C	3.410037000	-2.112562000	-1.725706000
H	3.046504000	-1.344515000	-2.395231000
C	-0.044429000	-6.309528000	0.330333000
C	-1.271021000	-5.745213000	-0.039622000
C	-2.170708000	-5.310403000	0.946627000
C	-1.824838000	-5.450403000	2.305791000
C	-0.600778000	-6.020441000	2.665730000
C	0.297348000	-6.450308000	1.679539000
H	0.636282000	-6.648693000	-0.443781000
H	-1.520009000	-5.654194000	-1.088907000
H	-2.510909000	-5.107369000	3.073464000
H	-0.344962000	-6.117273000	3.715313000
H	1.249532000	-6.886411000	1.960058000
S	-3.770463000	-4.610322000	0.568867000
C	-3.518637000	-3.898527000	-1.099221000
H	-3.443103000	-4.675143000	-1.862780000
H	-4.401045000	-3.287523000	-1.303513000
H	-2.629402000	-3.262425000	-1.111661000

^{3}IM1

Mn	-0.440285000	-0.327078000	-0.304126000
N	-0.478572000	-0.980471000	1.858435000
C	0.848967000	-1.569420000	2.422330000
C	1.854995000	-0.424224000	2.634846000
H	1.996664000	0.142541000	1.713769000
H	1.574594000	0.257885000	3.440325000
H	2.817958000	-0.867089000	2.903880000
C	0.562833000	-2.292683000	3.757127000
H	-0.010004000	-3.210696000	3.603896000
H	0.035266000	-1.661570000	4.478644000
H	1.521256000	-2.572026000	4.203544000
C	1.442963000	-2.554887000	1.412323000
H	2.307165000	-3.045026000	1.869921000
H	1.781187000	-2.023033000	0.527155000
H	0.740317000	-3.332538000	1.116175000
C	-1.599926000	-2.001622000	1.807957000
H	-1.903447000	-2.284956000	2.817601000
H	-1.228370000	-2.887129000	1.293762000
C	-2.758180000	-1.412665000	1.049756000
C	-4.099491000	-1.711698000	1.257641000
H	-4.385166000	-2.425025000	2.017845000
C	-5.061140000	-1.079390000	0.458209000
H	-6.109478000	-1.315870000	0.589027000
C	-4.673821000	-0.134838000	-0.496298000
H	-5.407720000	0.378206000	-1.103856000
C	-3.315799000	0.153932000	-0.647812000
N	-2.408864000	-0.507129000	0.100742000
C	-2.789611000	1.253436000	-1.524525000
H	-3.401899000	1.352235000	-2.419164000
H	-2.893651000	2.194882000	-0.975911000
N	-1.325726000	1.093952000	-1.907856000
C	-1.159173000	0.662299000	-3.394980000
C	-1.716443000	1.751789000	-4.339771000
H	-2.785942000	1.932663000	-4.204411000
H	-1.573330000	1.405744000	-5.367693000
H	-1.182187000	2.699677000	-4.236213000
C	0.334988000	0.458254000	-3.701674000
H	0.859992000	1.409499000	-3.829393000
H	0.423459000	-0.088268000	-4.644699000

H	0.833637000	-0.112467000	-2.920475000
C	-1.927573000	-0.652312000	-3.599712000
H	-1.553517000	-1.423637000	-2.928128000
H	-1.785640000	-0.983003000	-4.632183000
H	-3.003191000	-0.538957000	-3.439575000
C	-0.579483000	2.374910000	-1.601299000
H	-1.043223000	3.220404000	-2.116114000
H	0.438024000	2.266563000	-1.977228000
C	-0.564397000	2.616393000	-0.115810000
C	-0.545021000	3.868241000	0.492933000
H	-0.535472000	4.767420000	-0.108864000
C	-0.548291000	3.933558000	1.892613000
H	-0.518751000	4.896234000	2.387528000
C	-0.622476000	2.761209000	2.652547000
H	-0.664624000	2.799280000	3.733251000
C	-0.666199000	1.534079000	1.990640000
N	-0.592447000	1.492958000	0.642140000
C	-0.937753000	0.222317000	2.667503000
H	-0.500110000	0.192143000	3.664002000
H	-2.020584000	0.143716000	2.803351000
O	1.294048000	-0.041943000	-0.668080000
H	1.660665000	0.794563000	-0.344621000
O	-0.575483000	-1.873817000	-0.969554000
I	1.160252000	-3.130345000	-2.405974000
C	3.010412000	-3.253167000	-1.421832000
C	3.363747000	-4.449314000	-0.789747000
H	2.717061000	-5.317340000	-0.837844000
C	4.569321000	-4.500333000	-0.088907000
H	4.858557000	-5.421805000	0.405496000
C	5.391557000	-3.373285000	-0.016843000
H	6.323005000	-3.418484000	0.537621000
C	5.016693000	-2.186362000	-0.652895000
H	5.653723000	-1.309931000	-0.593752000
C	3.819229000	-2.113934000	-1.363799000
H	3.503836000	-1.188341000	-1.828640000
C	-0.504290000	-5.578405000	0.945464000
C	-1.476869000	-5.239634000	-0.002130000
C	-2.810516000	-5.041823000	0.397659000
C	-3.151696000	-5.190866000	1.755814000
C	-2.173657000	-5.530740000	2.695847000
C	-0.844446000	-5.722660000	2.296433000
H	0.520235000	-5.730831000	0.623829000
H	-1.191314000	-5.134095000	-1.040426000
H	-4.177419000	-5.035828000	2.073465000
H	-2.449626000	-5.636316000	3.739437000
H	-0.085683000	-5.982009000	3.026046000
S	-4.115673000	-4.629395000	-0.747352000
C	-3.203119000	-3.743544000	-2.062970000
H	-2.669580000	-4.431035000	-2.722733000
H	-3.947320000	-3.196274000	-2.645428000
H	-2.497987000	-3.038861000	-1.617215000

^{5}TS$_{ET}$

Mn	-0.583740000	-0.306395000	-0.329122000
N	-0.630841000	-0.828134000	1.872434000
C	0.618501000	-1.566976000	2.441311000
C	1.804717000	-0.588027000	2.465340000
H	2.013975000	-0.221360000	1.461174000
H	1.652225000	0.257851000	3.140121000
H	2.687852000	-1.129869000	2.815259000
C	0.322638000	-2.087451000	3.865558000
H	-0.419960000	-2.889622000	3.859077000
H	-0.012938000	-1.302155000	4.549057000
H	1.248918000	-2.502496000	4.273076000
C	0.971347000	-2.749249000	1.532169000
H	1.882964000	-3.218083000	1.913023000
H	1.158695000	-2.406317000	0.520111000
H	0.195607000	-3.515502000	1.510632000
C	-1.867924000	-1.700946000	1.918001000
H	-2.179702000	-1.880756000	2.949476000
H	-1.612869000	-2.655058000	1.460511000
C	-2.972609000	-1.045317000	1.139216000
C	-4.333224000	-1.159038000	1.414385000
H	-4.669470000	-1.753081000	2.252979000
C	-5.244077000	-0.497452000	0.581483000
H	-6.307499000	-0.588497000	0.763732000
C	-4.781903000	0.300580000	-0.470960000
H	-5.472885000	0.841831000	-1.104143000
C	-3.406242000	0.414576000	-0.678369000
N	-2.552226000	-0.282389000	0.100424000
C	-2.791466000	1.376612000	-1.653628000
H	-3.397959000	1.439470000	-2.555528000
H	-2.818042000	2.371818000	-1.197760000
N	-1.348477000	1.060204000	-2.010369000
C	-1.225527000	0.513777000	-3.464232000
C	-1.713704000	1.565680000	-4.486413000
H	-2.773801000	1.810866000	-4.381116000
H	-1.576455000	1.146750000	-5.487794000
H	-1.132210000	2.490125000	-4.436158000
C	0.248012000	0.184168000	-3.761165000
H	0.835246000	1.083801000	-3.969742000
H	0.288561000	-0.442579000	-4.656705000
H	0.710691000	-0.353710000	-2.935964000
C	-2.078749000	-0.760677000	-3.568520000
H	-1.718971000	-1.513774000	-2.867121000
H	-1.998718000	-1.151455000	-4.587066000
H	-3.138509000	-0.572199000	-3.373478000
C	-0.497629000	2.294770000	-1.796180000
H	-0.889825000	3.138443000	-2.370058000
H	0.504842000	2.072446000	-2.161876000
C	-0.445778000	2.639602000	-0.330730000
C	-0.315874000	3.924189000	0.190122000
H	-0.250967000	4.778870000	-0.470494000
C	-0.277753000	4.080529000	1.582719000
H	-0.163233000	5.068484000	2.011207000
C	-0.415213000	2.969654000	2.421863000
H	-0.421278000	3.082444000	3.498299000
C	-0.570138000	1.707703000	1.844458000
N	-0.542484000	1.576039000	0.502382000
C	-0.905753000	0.465909000	2.623397000
H	-0.392078000	0.460726000	3.583773000
H	-1.976238000	0.503138000	2.850013000
O	1.174048000	-0.173189000	-0.713587000
H	1.590002000	0.645434000	-0.406298000
O	-0.803343000	-1.894112000	-0.936219000
I	1.167110000	-3.362617000	-2.467749000
C	2.834951000	-3.364596000	-1.204951000
C	3.199049000	-4.544244000	-0.542307000
H	2.667083000	-5.468869000	-0.721784000
C	4.258596000	-4.505029000	0.361906000
H	4.548832000	-5.412099000	0.881733000
C	4.936794000	-3.306096000	0.603119000
H	5.755358000	-3.281785000	1.314846000
C	4.564539000	-2.139835000	-0.072119000
H	5.090571000	-1.209412000	0.115119000
C	3.512664000	-2.157834000	-0.985835000
H	3.187649000	-1.251677000	-1.478455000
C	-0.071677000	-6.147797000	-0.061062000
C	-1.255302000	-5.476733000	-0.374885000

C	-2.172579000	-5.161612000	0.647923000
C	-1.877125000	-5.516384000	1.985203000
C	-0.684964000	-6.171916000	2.285037000
C	0.223417000	-6.493410000	1.262629000
H	0.614321000	-6.405527000	-0.859233000
H	-1.465543000	-5.216238000	-1.402903000
H	-2.574689000	-5.263563000	2.776319000
H	-0.459153000	-6.428837000	3.313642000
H	1.148588000	-7.006405000	1.498504000
S	-3.718427000	-4.359270000	0.356230000
C	-3.480370000	-3.542849000	-1.250920000
H	-3.429302000	-4.265379000	-2.068125000
H	-4.351640000	-2.901330000	-1.398227000
H	-2.566101000	-2.935179000	-1.221755000

$^{3}TS_{ET}$

Mn	-0.544098000	-0.277052000	-0.280423000
N	-0.419758000	-0.877695000	1.921334000
C	0.914355000	-1.508011000	2.425350000
C	2.028068000	-0.454552000	2.321249000
H	2.202146000	-0.186812000	1.280305000
H	1.817281000	0.448830000	2.899424000
H	2.950245000	-0.889281000	2.716329000
C	0.763415000	-1.982437000	3.890110000
H	-0.000640000	-2.757003000	3.993430000
H	0.542913000	-1.172217000	4.589893000
H	1.717566000	-2.423094000	4.193347000
C	1.261169000	-2.716827000	1.550626000
H	2.293894000	-3.010500000	1.752050000
H	1.175989000	-2.471900000	0.496041000
H	0.626472000	-3.578343000	1.763304000
C	-1.564504000	-1.867562000	1.969436000
H	-1.797492000	-2.142846000	2.999943000
H	-1.252992000	-2.761723000	1.430310000
C	-2.762003000	-1.269077000	1.292775000
C	-4.088787000	-1.508991000	1.634576000
H	-4.326924000	-2.156798000	2.467045000
C	-5.098197000	-0.894606000	0.881311000
H	-6.138121000	-1.084304000	1.115586000
C	-4.765486000	-0.020058000	-0.156627000
H	-5.532851000	0.482216000	-0.730863000
C	-3.417897000	0.221919000	-0.430922000
N	-2.467570000	-0.425358000	0.273229000
C	-2.944982000	1.266325000	-1.397289000
H	-3.606195000	1.310936000	-2.260384000
H	-3.022497000	2.238485000	-0.900279000
N	-1.504207000	1.083727000	-1.851018000
C	-1.427687000	0.613573000	-3.337332000
C	-2.058036000	1.669909000	-4.273895000
H	-3.123407000	1.830241000	-4.090120000
H	-1.958733000	1.301840000	-5.299420000
H	-1.541076000	2.631388000	-4.220515000
C	0.046632000	0.421935000	-3.733148000
H	0.539387000	1.374821000	-3.948480000
H	0.084946000	-0.174882000	-4.648774000
H	0.608618000	-0.093296000	-2.956874000
C	-2.188351000	-0.714720000	-3.455335000
H	-1.752090000	-1.461430000	-2.794585000
H	-2.118235000	-1.066359000	-4.488417000
H	-3.250873000	-0.612014000	-3.217192000
C	-0.744010000	2.375713000	-1.633856000
H	-1.240071000	3.200775000	-2.151229000
H	0.250038000	2.253460000	-2.063948000
C	-0.642221000	2.668921000	-0.162117000
C	-0.581816000	3.939324000	0.402516000
H	-0.611952000	4.819231000	-0.226559000
C	-0.488285000	4.048025000	1.796844000
H	-0.426246000	5.025543000	2.258508000
C	-0.501830000	2.899855000	2.595353000
H	-0.462082000	2.971282000	3.674562000
C	-0.590202000	1.651731000	1.976047000
N	-0.619920000	1.569409000	0.630111000
C	-0.780493000	0.363735000	2.722561000
H	-0.221214000	0.378086000	3.655890000
H	-1.837124000	0.293907000	2.999058000
O	1.163834000	0.038044000	-0.741081000
H	1.513964000	0.880811000	-0.415925000
O	-0.661453000	-1.810550000	-0.953131000
I	1.460808000	-2.927322000	-2.498615000
C	3.084355000	-3.159236000	-1.178303000
C	3.373402000	-4.426919000	-0.667527000
H	2.803366000	-5.294935000	-0.977634000
C	4.404212000	-4.550985000	0.266114000
H	4.638649000	-5.529598000	0.672346000
C	5.120111000	-3.425723000	0.681791000
H	5.914035000	-3.529087000	1.414075000
C	4.811836000	-2.166680000	0.159677000
H	5.361418000	-1.289868000	0.486654000
C	3.788774000	-2.021411000	-0.777968000
H	3.514414000	-1.044427000	-1.155489000
C	-0.574935000	-5.802545000	0.931855000
C	-1.157305000	-5.066712000	-0.105157000
C	-2.469607000	-4.581800000	0.030317000
C	-3.187971000	-4.842870000	1.213039000
C	-2.588394000	-5.556811000	2.254724000
C	-1.280692000	-6.040716000	2.118348000
H	0.439614000	-6.169224000	0.817846000
H	-0.581953000	-4.853018000	-0.995630000
H	-4.210019000	-4.494924000	1.310600000
H	-3.147545000	-5.745534000	3.164563000
H	-0.818787000	-6.598798000	2.924871000
S	-3.248336000	-3.556371000	-1.198685000
C	-2.525833000	-4.176215000	-2.756986000
H	-2.634255000	-5.260571000	-2.819474000
H	-3.085729000	-3.706243000	-3.567798000
H	-1.475781000	-3.898890000	-2.844430000

^{5}IM2

Mn	-0.778464000	-0.265583000	-0.247711000
N	-0.672146000	-0.781255000	1.984650000
C	0.602405000	-1.540985000	2.453006000
C	1.803098000	-0.583869000	2.379127000
H	1.960250000	-0.248056000	1.355147000
H	1.703327000	0.282172000	3.037672000
H	2.695670000	-1.131543000	2.691274000
C	0.414810000	-2.046144000	3.901242000
H	-0.361658000	-2.813107000	3.966602000
H	0.177677000	-1.244101000	4.606444000
H	1.354328000	-2.501785000	4.227055000
C	0.853674000	-2.741020000	1.531014000
H	1.791339000	-3.216396000	1.829508000
H	0.943151000	-2.434362000	0.491064000

H	0.068796000	-3.491783000	1.615452000
C	-1.924713000	-1.615000000	2.137357000
H	-2.172715000	-1.752352000	3.193274000
H	-1.723021000	-2.593104000	1.705792000
C	-3.068735000	-0.954318000	1.415171000
C	-4.415348000	-1.088205000	1.744897000
H	-4.713901000	-1.707845000	2.580410000
C	-5.365141000	-0.400211000	0.977518000
H	-6.418897000	-0.502684000	1.205297000
C	-4.956250000	0.440908000	-0.063544000
H	-5.677194000	1.004729000	-0.641207000
C	-3.592417000	0.556077000	-0.336069000
N	-2.704464000	-0.166781000	0.377654000
C	-2.996542000	1.538381000	-1.304629000
H	-3.668662000	1.728374000	-2.140321000
H	-2.883826000	2.490386000	-0.777044000
N	-1.623048000	1.121065000	-1.812288000
C	-1.699894000	0.538644000	-3.253671000
C	-1.984306000	1.668367000	-4.266428000
H	-2.870268000	2.255106000	-4.005158000
H	-2.169451000	1.213152000	-5.243599000
H	-1.133071000	2.345613000	-4.374089000
C	-0.374007000	-0.154100000	-3.604583000
H	0.481529000	0.523192000	-3.552998000
H	-0.444288000	-0.520634000	-4.633630000
H	-0.188952000	-0.997585000	-2.941530000
C	-2.834762000	-0.499397000	-3.289331000
H	-2.724897000	-1.198165000	-2.460803000
H	-2.766852000	-1.053781000	-4.229293000
H	-3.828281000	-0.046776000	-3.251792000
C	-0.683982000	2.307344000	-1.716334000
H	-1.095427000	3.166500000	-2.252162000
H	0.257024000	2.026925000	-2.190668000
C	-0.461163000	2.654511000	-0.265490000
C	-0.181908000	3.925144000	0.230198000
H	-0.100267000	4.769817000	-0.441333000
C	-0.018348000	4.081587000	1.613944000
H	0.213014000	5.057880000	2.021551000
C	-0.180047000	2.989262000	2.473231000
H	-0.088681000	3.105283000	3.545350000
C	-0.483922000	1.742127000	1.923518000
N	-0.576587000	1.606416000	0.585145000
C	-0.853704000	0.528972000	2.731061000
H	-0.309560000	0.507902000	3.674800000
H	-1.913051000	0.623910000	2.990173000
O	0.943775000	-0.180364000	-0.776673000
H	1.413923000	0.605775000	-0.463484000
O	-1.097861000	-1.826791000	-0.858895000
I	2.156387000	-3.494664000	-3.212094000
C	3.174532000	-3.417976000	-1.351334000
C	3.453505000	-4.609091000	-0.679048000
H	3.152398000	-5.563985000	-1.094065000
C	4.141406000	-4.551887000	0.535753000
H	4.359397000	-5.473375000	1.066318000
C	4.552001000	-3.323358000	1.057240000
H	5.092671000	-3.285330000	1.997330000
C	4.262201000	-2.144007000	0.368832000
H	4.572158000	-1.185697000	0.772559000
C	3.562340000	-2.179710000	-0.839212000
H	3.301679000	-1.262578000	-1.351467000
C	0.227425000	-6.140127000	0.475637000
C	-0.428795000	-5.304802000	-0.428175000
C	-1.679478000	-4.758872000	-0.079736000
C	-2.260988000	-5.049399000	1.174615000
C	-1.587129000	-5.878821000	2.070993000
C	-0.343541000	-6.425845000	1.724095000
H	1.193068000	-6.554588000	0.213335000
H	0.037338000	-5.067800000	-1.374857000
H	-3.230344000	-4.637879000	1.434453000
H	-2.032658000	-6.101514000	3.033165000
H	0.178577000	-7.068775000	2.422979000
S	-2.565376000	-3.648166000	-1.107267000
C	-1.854087000	-3.916601000	-2.752005000
H	-1.843435000	-4.983968000	-2.977708000
H	-2.493419000	-3.395008000	-3.462894000
H	-0.848766000	-3.497345000	-2.786439000

^{3}IM2

Mn	-0.754111000	-0.288627000	-0.250255000
N	-0.647059000	-0.798442000	1.982929000
C	0.637623000	-1.535848000	2.466818000
C	1.824849000	-0.563714000	2.383055000
H	1.987964000	-0.250793000	1.352934000
H	1.704614000	0.316079000	3.019694000
H	2.721699000	-1.090477000	2.718578000
C	0.449873000	-2.023413000	3.921242000
H	-0.327870000	-2.787955000	3.997266000
H	0.218300000	-1.214197000	4.619639000
H	1.389380000	-2.478401000	4.247953000
C	0.903802000	-2.746817000	1.564568000
H	1.861843000	-3.187751000	1.850136000
H	0.959767000	-2.460655000	0.516704000
H	0.144385000	-3.519514000	1.682624000
C	-1.884198000	-1.655667000	2.123755000
H	-2.143224000	-1.793881000	3.176618000
H	-1.656604000	-2.632407000	1.702135000
C	-3.030147000	-1.017104000	1.386065000
C	-4.376180000	-1.158622000	1.712892000
H	-4.670525000	-1.772379000	2.553995000
C	-5.329889000	-0.486996000	0.936670000
H	-6.383272000	-0.597766000	1.161602000
C	-4.925201000	0.352643000	-0.107037000
H	-5.649047000	0.908732000	-0.688285000
C	-3.562674000	0.481670000	-0.376659000
N	-2.668590000	-0.234946000	0.340389000
C	-2.979490000	1.479902000	-1.335376000
H	-3.644944000	1.654003000	-2.179542000
H	-2.900959000	2.432935000	-0.803220000
N	-1.588877000	1.101210000	-1.824063000
C	-1.625415000	0.539052000	-3.276462000
C	-1.914506000	1.679412000	-4.276379000
H	-2.812185000	2.249351000	-4.017862000
H	-2.082046000	1.233581000	-5.261006000
H	-1.072138000	2.369973000	-4.365992000
C	-0.278329000	-0.118755000	-3.612517000
H	0.561368000	0.574758000	-3.527884000
H	-0.319268000	-0.463067000	-4.650705000
H	-0.088903000	-0.973935000	-2.966096000
C	-2.739996000	-0.516771000	-3.348286000
H	-2.652545000	-1.209031000	-2.513170000
H	-2.627904000	-1.078748000	-4.279010000
H	-3.741373000	-0.080276000	-3.350827000
C	-0.679361000	2.308228000	-1.703972000
H	-1.105495000	3.159212000	-2.240891000
H	0.273779000	2.054158000	-2.168251000
C	-0.485284000	2.652853000	-0.250165000
C	-0.241281000	3.927253000	0.253663000
H	-0.172266000	4.776636000	-0.413260000
C	-0.095395000	4.080971000	1.639654000

H	0.109219000	5.060241000	2.054202000
C	-0.240044000	2.980869000	2.491772000
H	-0.161761000	3.093539000	3.565230000
C	-0.510016000	1.730170000	1.932663000
N	-0.586637000	1.597365000	0.593082000
C	-0.860551000	0.507025000	2.731490000
H	-0.320797000	0.491348000	3.677549000
H	-1.922989000	0.578850000	2.985482000
O	0.984126000	-0.120981000	-0.743121000
H	1.389113000	0.690994000	-0.405320000
O	-0.902133000	-1.822200000	-0.918207000
I	2.047619000	-3.555543000	-3.194589000
C	3.127999000	-3.435685000	-1.371818000
C	3.447647000	-4.612731000	-0.692512000
H	3.145791000	-5.577622000	-1.083037000
C	4.175252000	-4.528603000	0.497273000
H	4.425200000	-5.439471000	1.032083000
C	4.583475000	-3.286935000	0.988847000
H	5.154300000	-3.227716000	1.909835000
C	4.250674000	-2.121891000	0.295758000
H	4.555867000	-1.153105000	0.677676000
C	3.512580000	-2.184826000	-0.888427000
H	3.216391000	-1.277375000	-1.398497000
C	0.147850000	-6.084411000	0.467321000
C	-0.520076000	-5.200311000	-0.381736000
C	-1.765553000	-4.674700000	0.008361000
C	-2.330503000	-5.031850000	1.250715000
C	-1.644084000	-5.906372000	2.095240000
C	-0.405972000	-6.435433000	1.705607000
H	1.109702000	-6.484134000	0.169582000
H	-0.063864000	-4.909934000	-1.318205000
H	-3.300687000	-4.643961000	1.542079000
H	-2.079334000	-6.180704000	3.049020000
H	0.123270000	-7.115625000	2.362821000
S	-2.659626000	-3.515693000	-0.976634000
C	-2.047741000	-3.859267000	-2.650576000
H	-2.015507000	-4.937157000	-2.813703000
H	-2.745979000	-3.401355000	-3.349727000
H	-1.058736000	-3.414193000	-2.770145000

$^{5}TS_{OT}$

Mn	-0.787424000	-0.262703000	0.065283000
N	-0.894163000	-0.372416000	2.386441000
C	0.278373000	-1.102045000	3.099361000
C	1.561413000	-0.275926000	2.905901000
H	1.810388000	-0.209270000	1.847421000
H	1.488230000	0.730098000	3.326512000
H	2.380919000	-0.784580000	3.421627000
C	-0.023369000	-1.271010000	4.605434000
H	-0.900493000	-1.900423000	4.778607000
H	-0.168893000	-0.317886000	5.121529000
H	0.833333000	-1.766599000	5.071464000
C	0.477325000	-2.490259000	2.469372000
H	1.411162000	-2.914112000	2.850347000
H	0.535814000	-2.422230000	1.384260000
H	-0.324993000	-3.180728000	2.744670000
C	-2.210681000	-1.085401000	2.573370000
H	-2.568363000	-0.981970000	3.602213000
H	-2.052967000	-2.144564000	2.381118000
C	-3.233962000	-0.532429000	1.617431000
C	-4.611321000	-0.550275000	1.825296000
H	-5.019592000	-0.979947000	2.730640000
C	-5.447466000	-0.001231000	0.843499000
H	-6.521803000	-0.021168000	0.977043000
C	-4.897341000	0.593874000	-0.296921000
H	-5.528773000	1.048318000	-1.049056000
C	-3.509011000	0.606663000	-0.443143000
N	-2.731506000	0.018072000	0.487758000
C	-2.785030000	1.340549000	-1.533800000
H	-3.359442000	1.315479000	-2.457388000
H	-2.719222000	2.392507000	-1.238405000
N	-1.367193000	0.835919000	-1.783409000
C	-1.273369000	0.051978000	-3.135314000
C	-1.664026000	0.959210000	-4.324330000
H	-2.704339000	1.292562000	-4.293639000
H	-1.540585000	0.371169000	-5.238722000
H	-1.014970000	1.834423000	-4.409756000
C	0.171977000	-0.434148000	-3.343176000
H	0.824870000	0.367223000	-3.702403000
H	0.166561000	-1.215624000	-4.107146000
H	0.593697000	-0.842941000	-2.426668000
C	-2.225491000	-1.147232000	-3.052144000
H	-1.919459000	-1.800379000	-2.236567000
H	-2.174170000	-1.703116000	-3.991766000
H	-3.266633000	-0.847560000	-2.900777000
C	-0.414654000	2.015424000	-1.776293000
H	-0.739991000	2.772783000	-2.493517000
H	0.563436000	1.651477000	-2.089815000
C	-0.330641000	2.605064000	-0.395759000
C	-0.054364000	3.938402000	-0.106727000
H	0.111333000	4.649060000	-0.905893000
C	-0.002325000	4.332464000	1.237265000
H	0.224925000	5.360782000	1.489858000
C	-0.269315000	3.407717000	2.252001000
H	-0.263922000	3.705215000	3.292790000
C	-0.567382000	2.089405000	1.899893000
N	-0.553336000	1.720651000	0.604264000
C	-1.041876000	1.053736000	2.877853000
H	-0.547495000	1.180242000	3.840426000
H	-2.105807000	1.240837000	3.056640000
O	0.987879000	-0.373163000	-0.273468000
H	1.454839000	0.463078000	-0.131191000
O	-1.120936000	-1.922324000	-0.230171000
I	-0.031096000	-4.395463000	-4.425502000
C	1.421293000	-4.409560000	-2.873824000
C	2.493250000	-5.300201000	-2.957570000
H	2.573878000	-5.995584000	-3.785064000
C	3.465539000	-5.280692000	-1.954661000
H	4.299535000	-5.973458000	-2.011123000
C	3.371603000	-4.374657000	-0.896231000
H	4.137534000	-4.356188000	-0.127292000
C	2.289852000	-3.496538000	-0.829413000
H	2.202864000	-2.776889000	-0.023417000
C	1.295366000	-3.516559000	-1.811075000
H	0.442872000	-2.858018000	-1.711243000
C	-0.846490000	-7.035367000	-1.876463000
C	-1.596143000	-5.935723000	-1.461902000
C	-1.205941000	-5.233260000	-0.304256000
C	-0.053484000	-5.630152000	0.416650000
C	0.692081000	-6.718509000	-0.021151000
C	0.298991000	-7.423204000	-1.168603000

H	-1.146932000	-7.578992000	-2.763930000
H	-2.466823000	-5.639443000	-2.029711000
H	0.251054000	-5.081112000	1.299159000
H	1.583179000	-7.010563000	0.520389000
H	0.885058000	-8.267940000	-1.510922000
S	-2.055710000	-3.850368000	0.343540000
C	-3.487967000	-3.626945000	-0.739932000
H	-4.174483000	-4.466177000	-0.614028000
H	-3.971914000	-2.704911000	-0.419054000
H	-3.180579000	-3.531917000	-1.780003000

$^{3}TS_{OT}$

Mn	-0.807837000	-0.363784000	0.182982000
N	-0.902036000	-0.414807000	2.398570000
C	0.266094000	-1.155595000	3.132216000
C	1.562454000	-0.345746000	2.963142000
H	1.837844000	-0.281604000	1.912042000
H	1.500003000	0.659002000	3.387116000
H	2.361480000	-0.870986000	3.493772000
C	-0.065118000	-1.314207000	4.632375000
H	-0.933046000	-1.957344000	4.797933000
H	-0.234177000	-0.358306000	5.135656000
H	0.791664000	-1.790335000	5.117353000
C	0.453483000	-2.543742000	2.505631000
H	1.364615000	-2.989935000	2.914302000
H	0.559950000	-2.476317000	1.423308000
H	-0.370997000	-3.216078000	2.754292000
C	-2.242564000	-1.068900000	2.667996000
H	-2.562960000	-0.868811000	3.693581000
H	-2.130031000	-2.146129000	2.567415000
C	-3.264043000	-0.540530000	1.693916000
C	-4.639144000	-0.527664000	1.910000000
H	-5.050394000	-0.913219000	2.833556000
C	-5.469368000	-0.006545000	0.907794000
H	-6.543306000	-0.002228000	1.045088000
C	-4.913076000	0.529174000	-0.258149000
H	-5.541133000	0.961495000	-1.025987000
C	-3.524953000	0.517528000	-0.415085000
N	-2.752987000	-0.045992000	0.539246000
C	-2.819382000	1.212806000	-1.547343000
H	-3.419170000	1.146490000	-2.453814000
H	-2.769681000	2.277172000	-1.292896000
N	-1.407084000	0.724461000	-1.811782000
C	-1.300706000	-0.050873000	-3.161079000
C	-1.680776000	0.860320000	-4.351147000
H	-2.719553000	1.200350000	-4.317132000
H	-1.561032000	0.275302000	-5.268130000
H	-1.027603000	1.732609000	-4.434305000
C	0.150592000	-0.532960000	-3.341409000
H	0.800485000	0.256128000	-3.732078000
H	0.162054000	-1.348939000	-4.068160000
H	0.564426000	-0.895653000	-2.400763000
C	-2.246119000	-1.258137000	-3.114841000
H	-1.924268000	-1.943691000	-2.335157000
H	-2.205998000	-1.771121000	-4.079804000
H	-3.286582000	-0.971202000	-2.935280000
C	-0.452772000	1.894963000	-1.813668000
H	-0.770030000	2.655656000	-2.531973000
H	0.523229000	1.524723000	-2.127370000
C	-0.344075000	2.496370000	-0.445484000
C	-0.053974000	3.834113000	-0.187603000
H	0.094771000	4.525707000	-1.006521000
C	0.036265000	4.257263000	1.143848000
H	0.276830000	5.288780000	1.369192000
C	-0.212803000	3.354759000	2.182951000
H	-0.183644000	3.672021000	3.217387000
C	-0.529153000	2.034483000	1.863044000
N	-0.546714000	1.630426000	0.574366000
C	-1.007051000	1.032530000	2.867078000
H	-0.496285000	1.155146000	3.820406000
H	-2.063507000	1.245293000	3.056895000
O	0.968768000	-0.505706000	-0.155285000
H	1.440803000	0.330072000	-0.025478000
O	-1.120274000	-1.954019000	-0.389417000
I	0.395588000	-4.413705000	-4.719755000
C	1.693175000	-4.418433000	-3.035611000
C	2.813679000	-5.251417000	-3.047827000
H	3.010791000	-5.904525000	-3.890135000
C	3.681516000	-5.228743000	-1.953430000
H	4.552865000	-5.876418000	-1.953945000
C	3.438108000	-4.374900000	-0.875504000
H	4.123966000	-4.352510000	-0.034430000
C	2.310479000	-3.553887000	-0.882060000
H	2.110472000	-2.871785000	-0.063816000
C	1.418774000	-3.579700000	-1.957178000
H	0.529334000	-2.966953000	-1.920567000
C	-0.866065000	-6.566087000	-2.032279000
C	-1.590837000	-5.488595000	-1.521394000
C	-1.239828000	-4.957439000	-0.268239000
C	-0.155815000	-5.494601000	0.456807000
C	0.571722000	-6.555373000	-0.078087000
C	0.218242000	-7.094431000	-1.321475000
H	-1.137246000	-6.978301000	-2.996235000
H	-2.409581000	-5.077128000	-2.095594000
H	0.112750000	-5.086924000	1.423254000
H	1.414386000	-6.956684000	0.471677000
H	0.786637000	-7.918660000	-1.735932000
S	-2.089193000	-3.616599000	0.489044000
C	-3.620537000	-3.444710000	-0.461246000
H	-4.179089000	-4.381021000	-0.414161000
H	-4.196995000	-2.653277000	0.017156000
H	-3.412935000	-3.168891000	-1.492748000

^{5}PC

Mn	-1.141496000	-0.510913000	-0.150420000
N	-1.170635000	-0.616486000	2.093002000
C	-0.028713000	-1.440185000	2.756399000
C	1.298434000	-0.684814000	2.562658000
H	1.481902000	-0.487705000	1.506163000
H	1.334935000	0.255514000	3.118227000
H	2.110105000	-1.314226000	2.938864000
C	-0.314277000	-1.634928000	4.260884000
H	-1.189027000	-2.268511000	4.430853000
H	-0.459160000	-0.688341000	4.789656000
H	0.546934000	-2.134226000	4.714114000
C	0.067426000	-2.816371000	2.077190000
H	0.885294000	-3.373013000	2.544832000
H	0.290168000	-2.718457000	1.015362000
H	-0.837915000	-3.411786000	2.203121000
C	-2.531376000	-1.226788000	2.351247000
H	-2.838264000	-1.083486000	3.390668000
H	-2.466410000	-2.300195000	2.168289000
C	-3.545500000	-0.621736000	1.421501000
C	-4.904289000	-0.492374000	1.691814000
H	-5.308876000	-0.853301000	2.627705000
C	-5.725260000	0.120701000	0.737090000
H	-6.788500000	0.212925000	0.919921000
C	-5.170604000	0.652170000	-0.432997000
H	-5.785140000	1.175934000	-1.153180000
C	-3.798984000	0.527141000	-0.645186000

N	-3.042736000	-0.154680000	0.249741000
C	-3.049464000	1.239548000	-1.733637000
H	-3.661141000	1.333537000	-2.630147000
H	-2.866448000	2.259410000	-1.378939000
N	-1.707740000	0.609515000	-2.054454000
C	-1.778179000	-0.236871000	-3.356543000
C	-2.066714000	0.654182000	-4.583417000
H	-3.008519000	1.203597000	-4.503329000
H	-2.140087000	0.010388000	-5.464955000
H	-1.258478000	1.368053000	-4.763761000
C	-0.436976000	-0.959319000	-3.577746000
H	0.354534000	-0.271791000	-3.888620000
H	-0.563739000	-1.686362000	-4.384911000
H	-0.101849000	-1.482393000	-2.683370000
C	-2.910135000	-1.264595000	-3.176225000
H	-2.794850000	-1.847522000	-2.259548000
H	-2.896511000	-1.958984000	-4.020735000
H	-3.898123000	-0.797275000	-3.156900000
C	-0.652641000	1.690255000	-2.122783000
H	-0.956123000	2.487021000	-2.808339000
H	0.261657000	1.245942000	-2.518505000
C	-0.396503000	2.270157000	-0.748642000
C	0.071263000	3.556361000	-0.485277000
H	0.290420000	4.239572000	-1.295599000
C	0.241087000	3.941645000	0.852592000
H	0.616568000	4.931378000	1.081727000
C	-0.098691000	3.069750000	1.895706000
H	-0.006520000	3.375219000	2.930082000
C	-0.583953000	1.802236000	1.570618000
N	-0.675118000	1.443768000	0.278555000
C	-1.168636000	0.825778000	2.564444000
H	-0.668967000	0.912234000	3.528637000
H	-2.209237000	1.124537000	2.728207000
O	0.501923000	-1.062553000	-0.545511000
H	1.202711000	-0.455991000	-0.827893000
O	-1.738933000	-6.453300000	-2.356086000
I	3.555430000	-1.022015000	-2.314011000
C	3.114937000	-3.033190000	-1.786335000
C	3.172980000	-4.023218000	-2.768157000
H	3.431543000	-3.777007000	-3.791457000
C	2.889470000	-5.344719000	-2.410045000
H	2.931987000	-6.120467000	-3.167900000
C	2.550287000	-5.663838000	-1.093803000
H	2.326669000	-6.690297000	-0.823123000
C	2.495648000	-4.657870000	-0.127175000
H	2.228472000	-4.898843000	0.896576000
C	2.780076000	-3.332796000	-0.465193000
H	2.730218000	-2.554014000	0.284545000
C	-4.616972000	-3.725310000	-0.185732000
C	-3.550812000	-4.245458000	-0.924711000
C	-2.518548000	-4.926172000	-0.264729000
C	-2.564703000	-5.125775000	1.118875000
C	-3.641948000	-4.613033000	1.855509000
C	-4.663127000	-3.910957000	1.203941000
H	-5.411473000	-3.185141000	-0.688527000
H	-3.531376000	-4.141199000	-2.003811000
H	-1.774543000	-5.680076000	1.614279000
H	-3.685676000	-4.768875000	2.927767000
H	-5.497617000	-3.518242000	1.773470000
S	-1.144982000	-5.666338000	-1.194431000
C	-0.502955000	-4.122920000	-1.917267000
H	-0.168509000	-3.446162000	-1.131628000
H	0.344259000	-4.386196000	-2.551357000
H	-1.285661000	-3.658468000	-2.517644000

^{3}PC

Mn	-1.129243000	-0.376902000	-0.117650000
N	-1.185064000	-0.609648000	2.019490000
C	-0.033026000	-1.441145000	2.675569000
C	1.283058000	-0.657006000	2.532409000
H	1.479077000	-0.420432000	1.486517000
H	1.296207000	0.260662000	3.125742000
H	2.097467000	-1.288992000	2.898164000
C	-0.343453000	-1.691603000	4.166637000
H	-1.200468000	-2.356715000	4.300474000
H	-0.522951000	-0.769494000	4.726881000
H	0.525219000	-2.181140000	4.616082000
C	0.093897000	-2.790321000	1.951652000
H	0.906060000	-3.353634000	2.421113000
H	0.341900000	-2.646641000	0.901264000
H	-0.807600000	-3.398987000	2.035048000
C	-2.541915000	-1.253307000	2.260699000
H	-2.828026000	-1.167873000	3.311327000
H	-2.465077000	-2.313681000	2.017977000
C	-3.563814000	-0.602340000	1.371453000
C	-4.928661000	-0.491984000	1.620470000
H	-5.354551000	-0.896507000	2.528745000
C	-5.730226000	0.157731000	0.671656000
H	-6.797253000	0.241125000	0.835885000
C	-5.156557000	0.723111000	-0.474511000
H	-5.761987000	1.254713000	-1.196826000
C	-3.779613000	0.607554000	-0.656982000
N	-3.040262000	-0.076334000	0.240967000
C	-2.980739000	1.284640000	-1.730650000
H	-3.548225000	1.370591000	-2.654943000
H	-2.772520000	2.306074000	-1.397850000
N	-1.642930000	0.592568000	-1.985867000
C	-1.718706000	-0.292337000	-3.274640000
C	-1.965006000	0.585117000	-4.521844000
H	-2.899374000	1.150137000	-4.479479000
H	-2.026690000	-0.077164000	-5.390313000
H	-1.140715000	1.280497000	-4.699848000
C	-0.395011000	-1.049578000	-3.472453000
H	0.411114000	-0.385734000	-3.796525000
H	-0.537795000	-1.789159000	-4.265260000
H	-0.072196000	-1.556767000	-2.566512000
C	-2.884623000	-1.282846000	-3.099011000
H	-2.791855000	-1.893920000	-2.199310000
H	-2.896034000	-1.958285000	-3.959034000
H	-3.855520000	-0.782150000	-3.070430000
C	-0.560112000	1.653173000	-2.081237000
H	-0.835099000	2.424177000	-2.804741000
H	0.354380000	1.174855000	-2.433637000
C	-0.347541000	2.259014000	-0.717650000
C	0.118227000	3.540811000	-0.442058000
H	0.375317000	4.212911000	-1.249985000
C	0.238288000	3.938017000	0.897682000
H	0.612191000	4.926520000	1.132924000
C	-0.143879000	3.075293000	1.932266000
H	-0.082433000	3.383618000	2.967678000
C	-0.625996000	1.808617000	1.604659000

N	-0.680681000	1.441166000	0.307963000
C	-1.215686000	0.815412000	2.565412000
H	-0.727438000	0.862804000	3.536893000
H	-2.262062000	1.090845000	2.727229000
O	0.519213000	-0.960137000	-0.531829000
H	1.210661000	-0.364183000	-0.854420000
O	-1.735704000	-6.511956000	-2.218309000
I	3.543319000	-0.954698000	-2.429155000
C	3.095438000	-2.961091000	-1.889928000
C	3.121290000	-3.952483000	-2.872031000
H	3.351038000	-3.708017000	-3.902608000
C	2.846214000	-5.273113000	-2.504105000
H	2.864944000	-6.049864000	-3.261903000
C	2.547470000	-5.590246000	-1.177461000
H	2.332048000	-6.616276000	-0.898386000
C	2.520624000	-4.582337000	-0.211765000
H	2.281976000	-4.820705000	0.819542000
C	2.795811000	-3.257967000	-0.559909000

H	2.765654000	-2.477202000	0.188683000
C	-4.679893000	-3.775211000	-0.106929000
C	-3.604941000	-4.289900000	-0.837140000
C	-2.567228000	-4.951998000	-0.166791000
C	-2.616597000	-5.137941000	1.219054000
C	-3.701502000	-4.629055000	1.947014000
C	-4.728406000	-3.945841000	1.284575000
H	-5.478215000	-3.248589000	-0.617821000
H	-3.582593000	-4.192638000	-1.916900000
H	-1.821554000	-5.677189000	1.723246000
H	-3.745379000	-4.771348000	3.021007000
H	-5.568371000	-3.554874000	1.847234000
S	-1.171405000	-5.675604000	-1.076724000
C	-0.577199000	-4.131609000	-1.838728000
H	-0.291967000	-3.414599000	-1.069282000
H	0.294248000	-4.378048000	-2.445947000
H	-1.365783000	-3.724011000	-2.471822000

C. Thioanisole sulfoxidation by complex 1 via the direct oxygen-atom transfer mechanism

^{5}RC

Mn	-0.501374000	-0.223673000	-0.257421000
N	-0.690189000	-0.753622000	2.051794000
C	0.539351000	-1.380330000	2.735048000
C	1.638596000	-0.305136000	2.841579000
H	1.861607000	0.108566000	1.855404000
H	1.380220000	0.508851000	3.523158000
H	2.549074000	-0.775029000	3.224916000
C	0.173460000	-1.919812000	4.133770000
H	-0.503998000	-2.776450000	4.069798000
H	-0.288503000	-1.157430000	4.769029000
H	1.086012000	-2.257500000	4.634133000
C	1.085263000	-2.517195000	1.862998000
H	1.935359000	-2.979266000	2.374207000
H	1.438127000	-2.113983000	0.917782000
H	0.355137000	-3.305195000	1.669824000
C	-1.858597000	-1.694434000	1.954531000
H	-2.280073000	-1.929138000	2.937137000
H	-1.511147000	-2.625235000	1.505015000
C	-2.928099000	-1.085904000	1.086105000
C	-4.294024000	-1.258988000	1.296546000
H	-4.637831000	-1.886111000	2.107047000
C	-5.196891000	-0.612091000	0.445796000
H	-6.262631000	-0.750325000	0.579653000
C	-4.721537000	0.243588000	-0.552645000
H	-5.404774000	0.791249000	-1.188945000
C	-3.344684000	0.415438000	-0.701791000
N	-2.490038000	-0.290057000	0.077292000
C	-2.761297000	1.477959000	-1.596730000
H	-3.400688000	1.617680000	-2.469085000
H	-2.807673000	2.421335000	-1.040451000
N	-1.337228000	1.227486000	-1.995245000
C	-1.219971000	0.731322000	-3.446923000
C	-1.747708000	1.778454000	-4.453554000
H	-2.814271000	1.986840000	-4.331321000
H	-1.606198000	1.390063000	-5.467058000
H	-1.196701000	2.720767000	-4.380962000
C	0.261933000	0.450940000	-3.771487000
H	0.799839000	1.371171000	-4.020110000
H	0.318337000	-0.202553000	-4.646715000
H	0.778463000	-0.028602000	-2.939667000
C	-2.050740000	-0.560511000	-3.559385000

H	-1.763718000	-1.280442000	-2.791295000
H	-1.891448000	-1.010204000	-4.543399000
H	-3.122837000	-0.370595000	-3.455843000
C	-0.478449000	2.423545000	-1.714781000
H	-0.829871000	3.315923000	-2.244327000
H	0.528659000	2.200907000	-2.070186000
C	-0.445923000	2.707886000	-0.233667000
C	-0.347122000	3.987282000	0.307769000
H	-0.260778000	4.845684000	-0.345733000
C	-0.379050000	4.136705000	1.699465000
H	-0.288322000	5.121318000	2.141282000
C	-0.570782000	3.018249000	2.515358000
H	-0.651613000	3.118767000	3.590111000
C	-0.686994000	1.758723000	1.924733000
N	-0.565212000	1.630937000	0.583388000
C	-1.097718000	0.533128000	2.702029000
H	-0.732631000	0.599204000	3.727878000
H	-2.190769000	0.557922000	2.772401000
O	1.263135000	-0.123650000	-0.653496000
H	1.731140000	0.672616000	-0.368513000
O	-0.600629000	-2.079497000	-0.852852000
I	0.613739000	-2.945200000	-2.065962000
C	2.459198000	-3.205244000	-1.094345000
C	2.682657000	-4.404565000	-0.413005000
H	1.912130000	-5.160300000	-0.342793000
C	3.919649000	-4.585307000	0.204547000
H	4.114744000	-5.504835000	0.745874000
C	4.893290000	-3.586091000	0.131835000
H	5.851610000	-3.732589000	0.618970000
C	4.644319000	-2.397059000	-0.561039000
H	5.404846000	-1.625319000	-0.612933000
C	3.416418000	-2.189930000	-1.186708000
H	3.193487000	-1.261922000	-1.694840000
C	-0.184333000	-6.619231000	-0.253734000
C	-1.380874000	-5.922744000	-0.456672000
C	-2.057868000	-5.356232000	0.636331000
C	-1.513695000	-5.484959000	1.928480000
C	-0.318287000	-6.183577000	2.123416000
C	0.350215000	-6.756227000	1.034237000
H	0.322947000	-7.063893000	-1.104039000
H	-1.787773000	-5.841120000	-1.457149000
H	-2.026003000	-5.038390000	2.774206000

H	0.091434000	-6.273756000	3.123600000
H	1.275521000	-7.301090000	1.186407000
S	-3.635245000	-4.532696000	0.481759000
C	-3.517772000	-3.744137000	-1.166379000
H	-3.549755000	-4.478982000	-1.972513000
H	-4.386393000	-3.087579000	-1.253865000
H	-2.605006000	-3.148287000	-1.225231000

^{3}RC

Mn	-0.594727000	-0.132501000	-0.173061000
N	-0.780418000	-0.779138000	1.897110000
C	0.452425000	-1.439893000	2.591486000
C	1.533581000	-0.364284000	2.806565000
H	1.786375000	0.114818000	1.859745000
H	1.247740000	0.396635000	3.536508000
H	2.432974000	-0.857240000	3.187220000
C	0.035867000	-2.060544000	3.943187000
H	-0.587648000	-2.947557000	3.805204000
H	-0.490340000	-1.358811000	4.596940000
H	0.942997000	-2.376798000	4.466235000
C	1.039613000	-2.531652000	1.692144000
H	1.839125000	-3.039086000	2.241074000
H	1.464421000	-2.078507000	0.802294000
H	0.309449000	-3.288544000	1.403838000
C	-1.959789000	-1.728668000	1.830699000
H	-2.343441000	-1.954040000	2.828345000
H	-1.618311000	-2.654522000	1.370421000
C	-3.031175000	-1.099902000	0.986002000
C	-4.403296000	-1.294979000	1.120285000
H	-4.785015000	-1.966775000	1.875916000
C	-5.265375000	-0.608830000	0.254518000
H	-6.335337000	-0.758825000	0.329164000
C	-4.752288000	0.291230000	-0.687154000
H	-5.409560000	0.851550000	-1.339289000
C	-3.371299000	0.475496000	-0.752595000
N	-2.559949000	-0.244916000	0.049355000
C	-2.686218000	1.528268000	-1.571634000
H	-3.231804000	1.721361000	-2.493521000
H	-2.713048000	2.461267000	-0.999233000
N	-1.230527000	1.192680000	-1.862460000
C	-1.068850000	0.690815000	-3.324761000
C	-1.499203000	1.762305000	-4.353236000
H	-2.558536000	2.023854000	-4.291271000
H	-1.326316000	1.351818000	-5.353094000
H	-0.905243000	2.676118000	-4.268396000
C	0.417495000	0.365146000	-3.576175000
H	0.977591000	1.263270000	-3.855166000
H	0.498548000	-0.338111000	-4.409145000
H	0.888300000	-0.067163000	-2.694430000
C	-1.967793000	-0.549633000	-3.481251000
H	-1.832657000	-1.246098000	-2.653536000
H	-1.726870000	-1.056254000	-4.419725000
H	-3.026959000	-0.279572000	-3.519847000
C	-0.378372000	2.416288000	-1.603016000
H	-0.737905000	3.278636000	-2.171088000
H	0.635531000	2.193358000	-1.936282000
C	-0.390451000	2.721047000	-0.130928000
C	-0.249201000	3.980538000	0.444923000
H	-0.092129000	4.851676000	-0.177518000
C	-0.328663000	4.091426000	1.840075000
H	-0.209089000	5.059254000	2.311123000
C	-0.595437000	2.963288000	2.624703000
H	-0.698654000	3.043292000	3.698987000
C	-0.747679000	1.729095000	1.993236000
N	-0.597355000	1.637777000	0.654022000
C	-1.202014000	0.467068000	2.663235000
H	-0.865968000	0.421230000	3.697812000
H	-2.295711000	0.491856000	2.697164000
O	1.195225000	-0.132601000	-0.489960000
H	1.670435000	0.688264000	-0.306135000
O	-0.683095000	-2.025394000	-0.758665000
I	0.413443000	-2.923883000	-2.057778000
C	2.365996000	-3.130672000	-1.284152000
C	2.686428000	-4.306096000	-0.601514000
H	1.938645000	-5.065165000	-0.416545000
C	3.991222000	-4.460264000	-0.132793000
H	4.260073000	-5.362678000	0.405897000
C	4.938146000	-3.456894000	-0.350043000
H	5.950157000	-3.582149000	0.020607000
C	4.592162000	-2.290573000	-1.039338000
H	5.331295000	-1.513900000	-1.204783000
C	3.294436000	-2.112589000	-1.516765000
H	3.007643000	-1.201541000	-2.023624000
C	-0.076397000	-6.503406000	0.110073000
C	-1.312534000	-5.913036000	-0.175756000
C	-2.082796000	-5.359620000	0.860200000
C	-1.595497000	-5.400879000	2.180660000
C	-0.358745000	-5.991471000	2.457817000
C	0.407299000	-6.544005000	1.423915000
H	0.499442000	-6.945405000	-0.696947000
H	-1.675722000	-5.903892000	-1.196016000
H	-2.184028000	-4.970871000	2.984334000
H	0.007288000	-6.011246000	3.478581000
H	1.366746000	-7.001347000	1.638672000
S	-3.703992000	-4.655858000	0.593881000
C	-3.545283000	-3.909317000	-1.071198000
H	-3.477314000	-4.667916000	-1.852726000
H	-4.450469000	-3.318966000	-1.229760000
H	-2.675442000	-3.249970000	-1.100018000

^{5}TS$_{DOT}$

Mn	-0.292671000	-0.003927000	-0.044154000
N	-0.397269000	-0.326208000	2.314297000
C	0.946058000	-0.579084000	3.030742000
C	1.775292000	0.718367000	2.979489000
H	1.896561000	1.052184000	1.946740000
H	1.338285000	1.525965000	3.571983000
H	2.768013000	0.513928000	3.391202000
C	0.708180000	-1.009752000	4.494068000
H	0.212632000	-1.983369000	4.553005000
H	0.111067000	-0.281881000	5.051948000
H	1.674261000	-1.098500000	4.999657000
C	1.731827000	-1.676993000	2.295963000
H	2.656705000	-1.869465000	2.848118000
H	1.999084000	-1.350118000	1.292689000
H	1.189656000	-2.621983000	2.226393000
C	-1.335317000	-1.488921000	2.395389000
H	-1.787791000	-1.574906000	3.389610000
H	-0.777121000	-2.400707000	2.198790000
C	-2.428804000	-1.366942000	1.364326000
C	-3.666279000	-1.995656000	1.488491000
H	-3.867502000	-2.623696000	2.346397000

C	-4.631033000	-1.794337000	0.496801000
H	-5.589551000	-2.293851000	0.563433000
C	-4.374340000	-0.911761000	-0.558627000
H	-5.131593000	-0.697377000	-1.301451000
C	-3.131358000	-0.283990000	-0.632205000
N	-2.175965000	-0.581324000	0.285286000
C	-2.857356000	0.844535000	-1.596139000
H	-3.475433000	0.719211000	-2.484965000
H	-3.215633000	1.759406000	-1.109332000
N	-1.411758000	1.038812000	-1.961966000
C	-1.108524000	0.573949000	-3.403937000
C	-1.958056000	1.336445000	-4.446392000
H	-3.027338000	1.123294000	-4.363987000
H	-1.641827000	1.020416000	-5.445397000
H	-1.810507000	2.418275000	-4.382027000
C	0.381971000	0.826999000	-3.713123000
H	0.563747000	1.857442000	-4.031809000
H	0.694226000	0.176948000	-4.535287000
H	1.012181000	0.615139000	-2.849341000
C	-1.414048000	-0.926537000	-3.485098000
H	-0.800852000	-1.480670000	-2.776489000
H	-1.200586000	-1.288277000	-4.494837000
H	-2.465596000	-1.145811000	-3.279210000
C	-0.992015000	2.461621000	-1.757861000
H	-1.622604000	3.156405000	-2.323896000
H	0.024990000	2.569932000	-2.136126000
C	-1.025170000	2.850976000	-0.306705000
C	-1.239633000	4.162804000	0.112487000
H	-1.385240000	4.944228000	-0.622087000
C	-1.272579000	4.440739000	1.482025000
H	-1.417929000	5.456413000	1.828497000
C	-1.159599000	3.393648000	2.400265000
H	-1.237738000	3.573170000	3.464874000
C	-0.977419000	2.093385000	1.929637000
N	-0.852709000	1.858270000	0.601062000
C	-1.086259000	0.898589000	2.833339000
H	-0.748557000	1.152228000	3.839036000
H	-2.155822000	0.677335000	2.923494000
O	1.454619000	0.443087000	-0.383743000
H	1.715821000	1.366576000	-0.496928000
O	0.183258000	-1.838393000	-0.641428000
I	1.955500000	-1.851977000	-1.597565000
C	1.637744000	-3.674660000	-2.572113000
C	1.129437000	-3.648444000	-3.877080000
H	0.945980000	-2.710979000	-4.387484000
C	0.851850000	-4.860811000	-4.507301000
H	0.454159000	-4.859820000	-5.516307000
C	1.075105000	-6.066728000	-3.836848000
H	0.845602000	-7.005867000	-4.329288000
C	1.597637000	-6.074459000	-2.540216000
H	1.773145000	-7.013036000	-2.026129000
C	1.888184000	-4.874289000	-1.893564000
H	2.279647000	-4.871117000	-0.885009000
C	-1.354348000	-7.678990000	0.702931000
C	-1.146395000	-6.453733000	0.058226000
C	-0.825035000	-5.303565000	0.801892000
C	-0.698305000	-5.412632000	2.198189000
C	-0.900579000	-6.639458000	2.839967000
C	-1.233950000	-7.777328000	2.094721000
H	-1.603378000	-8.557154000	0.115390000
H	-1.223201000	-6.402329000	-1.021912000
H	-0.445446000	-4.537582000	2.788967000
H	-0.805395000	-6.703462000	3.919218000
H	-1.395426000	-8.728075000	2.591406000
S	-0.411440000	-3.695952000	0.034543000
C	-1.626006000	-3.778976000	-1.340920000
H	-2.491925000	-4.354333000	-1.013398000
H	-1.941214000	-2.768742000	-1.580442000
H	-1.190336000	-4.240426000	-2.229247000

$^{3}TS_{DOT}$

Mn	-0.376412000	0.133008000	0.018154000
N	-0.423143000	-0.231746000	2.210912000
C	0.935869000	-0.466190000	2.940818000
C	1.728816000	0.853338000	2.941447000
H	1.843589000	1.231902000	1.924547000
H	1.277090000	1.625152000	3.568864000
H	2.726554000	0.652600000	3.342403000
C	0.681290000	-0.941639000	4.388338000
H	0.256488000	-1.948728000	4.415526000
H	0.026091000	-0.270210000	4.950666000
H	1.641076000	-0.974343000	4.911530000
C	1.759878000	-1.527709000	2.199117000
H	2.631287000	-1.774635000	2.813202000
H	2.113000000	-1.129929000	1.250567000
H	1.207978000	-2.450821000	2.017263000
C	-1.348019000	-1.416826000	2.349624000
H	-1.746903000	-1.481418000	3.365958000
H	-0.786929000	-2.323960000	2.136347000
C	-2.472172000	-1.302181000	1.357448000
C	-3.699206000	-1.952467000	1.466252000
H	-3.894117000	-2.608643000	2.304151000
C	-4.665180000	-1.735767000	0.476980000
H	-5.618227000	-2.246893000	0.532382000
C	-4.417967000	-0.833922000	-0.566281000
H	-5.173638000	-0.623490000	-1.311599000
C	-3.181905000	-0.192901000	-0.622158000
N	-2.232674000	-0.485344000	0.300642000
C	-2.856522000	0.927448000	-1.570923000
H	-3.440944000	0.833148000	-2.484411000
H	-3.181894000	1.857945000	-1.093259000
N	-1.377161000	1.054931000	-1.886685000
C	-1.052892000	0.511914000	-3.305866000
C	-1.849481000	1.259909000	-4.399805000
H	-2.926990000	1.085048000	-4.345510000
H	-1.512074000	0.886859000	-5.371722000
H	-1.665858000	2.337549000	-4.382212000
C	0.450629000	0.708587000	-3.587099000
H	0.664148000	1.714502000	-3.960475000
H	0.764565000	0.006006000	-4.364017000
H	1.052430000	0.533363000	-2.696409000
C	-1.420744000	-0.975700000	-3.326339000
H	-0.871330000	-1.518061000	-2.559388000
H	-1.178288000	-1.393998000	-4.307044000
H	-2.490324000	-1.137033000	-3.164521000
C	-0.938075000	2.492257000	-1.756643000
H	-1.551766000	3.152156000	-2.377130000
H	0.088235000	2.561393000	-2.117346000
C	-1.002150000	2.937391000	-0.325879000
C	-1.209442000	4.253951000	0.078060000
H	-1.325820000	5.034970000	-0.661863000
C	-1.274452000	4.538248000	1.446947000
H	-1.417997000	5.557888000	1.782140000
C	-1.193800000	3.499343000	2.380070000
H	-1.291954000	3.691288000	3.440503000
C	-1.013773000	2.196132000	1.921279000
N	-0.865598000	1.952451000	0.596648000
C	-1.131666000	0.981969000	2.787062000
H	-0.795698000	1.183048000	3.803070000

H	-2.198201000	0.744588000	2.857003000
O	1.380059000	0.470252000	-0.345040000
H	1.700647000	1.379742000	-0.407837000
O	0.118851000	-1.896626000	-0.545249000
I	1.854152000	-2.000786000	-1.478974000
C	1.527907000	-3.783107000	-2.540470000
C	1.059134000	-3.692171000	-3.855409000
H	0.895718000	-2.730293000	-4.325000000
C	0.797815000	-4.872623000	-4.552431000
H	0.428446000	-4.820460000	-5.570914000
C	1.004521000	-6.109610000	-3.937838000
H	0.790566000	-7.023465000	-4.482153000
C	1.490573000	-6.180053000	-2.628762000
H	1.653100000	-7.142437000	-2.155877000
C	1.760467000	-5.013285000	-1.915292000
H	2.116619000	-5.059601000	-0.894967000
C	-1.204977000	-7.818371000	0.627097000
C	-1.059866000	-6.555786000	0.039246000
C	-0.783658000	-5.427148000	0.832651000
C	-0.637358000	-5.595724000	2.221541000
C	-0.773816000	-6.859448000	2.806617000
C	-1.062691000	-7.976090000	2.011485000
H	-1.420938000	-8.679059000	0.001529000
H	-1.150478000	-6.456280000	-1.036775000
H	-0.422672000	-4.733229000	2.846516000
H	-0.664376000	-6.969972000	3.880963000
H	-1.173866000	-8.955656000	2.464314000
S	-0.440366000	-3.766236000	0.148999000
C	-1.690490000	-3.798993000	-1.196975000
H	-2.490402000	-4.486329000	-0.922136000
H	-2.102511000	-2.800175000	-1.310357000
H	-1.245070000	-4.111347000	-2.144224000

[5]pc

Mn	-1.233878000	-0.743986000	-0.211123000
N	-0.700706000	-1.340621000	1.907381000
C	0.482756000	-2.335106000	2.068411000
C	1.791810000	-1.602249000	1.727917000
H	1.736841000	-1.135217000	0.744742000
H	2.062442000	-0.845773000	2.468627000
H	2.600310000	-2.337449000	1.697229000
C	0.537196000	-2.876481000	3.512631000
H	-0.312306000	-3.528563000	3.733211000
H	0.574471000	-2.079580000	4.261641000
H	1.446412000	-3.474596000	3.623158000
C	0.292205000	-3.499929000	1.090577000
H	1.123340000	-4.198990000	1.205948000
H	0.302843000	-3.145719000	0.060997000
H	-0.623679000	-4.060385000	1.275865000
C	-2.008335000	-1.942269000	2.376207000
H	-2.034071000	-2.035858000	3.465365000
H	-2.087655000	-2.945666000	1.955838000
C	-3.154714000	-1.090648000	1.903874000
C	-4.384270000	-0.969575000	2.544707000
H	-4.586576000	-1.535492000	3.444372000
C	-5.336890000	-0.091650000	2.011231000
H	-6.304148000	0.007933000	2.487845000
C	-5.031124000	0.686159000	0.887973000
H	-5.741002000	1.402420000	0.495984000
C	-3.781269000	0.541153000	0.289128000
N	-2.907281000	-0.371811000	0.779030000
C	-3.251146000	1.432597000	-0.797495000
H	-4.054114000	1.785856000	-1.443725000
H	-2.836807000	2.321050000	-0.309678000
N	-2.144313000	0.783096000	-1.605732000
C	-2.675652000	0.260604000	-2.973464000
C	-3.043692000	1.446665000	-3.891958000
H	-3.762854000	2.130746000	-3.432140000
H	-3.504684000	1.051747000	-4.801791000
H	-2.160718000	2.017370000	-4.191565000
C	-1.605596000	-0.598651000	-3.668410000
H	-0.692800000	-0.038189000	-3.885249000
H	-2.014466000	-0.938361000	-4.624829000
H	-1.333709000	-1.471877000	-3.077134000
C	-3.925615000	-0.605579000	-2.721257000
H	-3.726540000	-1.435461000	-2.039155000
H	-4.241332000	-1.035369000	-3.676062000
H	-4.768706000	-0.029992000	-2.332296000
C	-1.001144000	1.764125000	-1.763819000
H	-1.354644000	2.706173000	-2.191972000
H	-0.279944000	1.328585000	-2.456708000
C	-0.341827000	2.028798000	-0.426147000
C	0.318099000	3.199692000	-0.057531000
H	0.408360000	4.024482000	-0.752439000
C	0.850312000	3.282950000	1.238161000
H	1.379064000	4.177769000	1.542827000
C	0.677993000	2.234706000	2.152460000
H	1.051623000	2.311178000	3.165455000
C	-0.009681000	1.094688000	1.733483000
N	-0.448113000	1.023845000	0.465247000
C	-0.443983000	-0.037428000	2.637181000
H	0.272162000	-0.183689000	3.445496000
H	-1.381970000	0.274570000	3.108330000
O	0.155523000	-1.220848000	-1.207365000
H	0.983505000	-0.718349000	-1.224790000
O	-1.621452000	-6.525477000	-2.412086000
I	3.575638000	-1.368996000	-2.032192000
C	2.939667000	-3.338153000	-1.539877000
C	1.806929000	-3.868742000	-2.159057000
H	1.237014000	-3.279630000	-2.867080000
C	1.403101000	-5.165843000	-1.828881000
H	0.508875000	-5.586361000	-2.281329000
C	2.129743000	-5.911659000	-0.897875000
H	1.813825000	-6.918338000	-0.644867000
C	3.257550000	-5.363233000	-0.286001000
H	3.821433000	-5.937870000	0.442080000
C	3.671664000	-4.065945000	-0.601362000
H	4.545743000	-3.638179000	-0.124035000
C	-0.929735000	-6.882642000	1.847485000
C	-1.228739000	-6.723315000	0.489181000
C	-2.323176000	-5.940517000	0.118360000
C	-3.146711000	-5.339443000	1.079102000
C	-2.841075000	-5.503767000	2.435265000
C	-1.730183000	-6.269545000	2.819061000
H	-0.073733000	-7.478488000	2.144023000
H	-0.627803000	-7.189033000	-0.282263000
H	-4.011635000	-4.753526000	0.783693000
H	-3.469137000	-5.039782000	3.187927000
H	-1.497550000	-6.393213000	3.870791000
S	-2.704811000	-5.771917000	-1.650037000
C	-2.311641000	-3.991008000	-1.791670000

H	-1.256390000	-3.835896000	-1.560990000
H	-2.522813000	-3.689559000	-2.819072000
H	-2.936855000	-3.422869000	-1.099075000

^{3}pc

Mn	-1.183999000	-0.212526000	-0.180435000
N	-1.181088000	-0.977922000	1.834231000
C	-0.000033000	-1.923007000	2.228370000
C	1.299104000	-1.101821000	2.287692000
H	1.492644000	-0.614277000	1.332680000
H	1.298332000	-0.355393000	3.085741000
H	2.125262000	-1.789185000	2.491439000
C	-0.277284000	-2.577086000	3.598999000
H	-1.106418000	-3.287726000	3.553254000
H	-0.480223000	-1.846820000	4.388001000
H	0.614891000	-3.136873000	3.893313000
C	0.133675000	-3.012920000	1.159635000
H	0.957736000	-3.673287000	1.434772000
H	0.359612000	-2.577735000	0.187878000
H	-0.753947000	-3.640075000	1.085544000
C	-2.517215000	-1.699573000	1.940692000
H	-2.773038000	-1.875013000	2.987796000
H	-2.420409000	-2.670577000	1.453460000
C	-3.576281000	-0.876341000	1.261783000
C	-4.936127000	-0.871962000	1.559170000
H	-5.329129000	-1.513657000	2.336788000
C	-5.775253000	-0.013517000	0.835101000
H	-6.838439000	-0.001586000	1.040015000
C	-5.243272000	0.847604000	-0.133137000
H	-5.877052000	1.536539000	-0.675756000
C	-3.871265000	0.813398000	-0.374548000
N	-3.092763000	-0.061810000	0.296240000
C	-3.115085000	1.757529000	-1.260686000
H	-3.708578000	2.067921000	-2.118591000
H	-2.906211000	2.663465000	-0.684007000
N	-1.780487000	1.171126000	-1.714227000
C	-1.890214000	0.615489000	-3.172004000
C	-2.087214000	1.780474000	-4.166701000
H	-2.947529000	2.410669000	-3.926051000
H	-2.263763000	1.354233000	-5.158274000
H	-1.197904000	2.412387000	-4.234786000
C	-0.616336000	-0.151502000	-3.557615000
H	0.275434000	0.480276000	-3.545451000
H	-0.742378000	-0.513750000	-4.581988000
H	-0.428523000	-1.005663000	-2.911688000
C	-3.099330000	-0.338269000	-3.237364000
H	-3.050193000	-1.125703000	-2.482296000
H	-3.100694000	-0.821114000	-4.218402000
H	-4.053779000	0.182480000	-3.132727000
C	-0.712870000	2.244203000	-1.579031000
H	-1.017440000	3.160897000	-2.088612000
H	0.196180000	1.876870000	-2.055339000
C	-0.476887000	2.503467000	-0.112323000
C	-0.043333000	3.692345000	0.465749000
H	0.173986000	4.552561000	-0.153407000
C	0.094096000	3.748993000	1.860469000
H	0.442099000	4.661023000	2.328772000
C	-0.238810000	2.644326000	2.654159000
H	-0.164338000	2.689462000	3.732643000
C	-0.689097000	1.481621000	2.030252000
N	-0.761439000	1.445777000	0.683711000
C	-1.227015000	0.261509000	2.723167000
H	-0.711344000	0.075671000	3.663853000
H	-2.273827000	0.457380000	2.974031000
O	0.489543000	-0.549320000	-0.776654000
H	1.167628000	0.135467000	-0.694796000
O	-0.942772000	-6.025280000	-3.201729000
I	1.667307000	-3.147401000	-4.622835000
C	2.447074000	-3.953107000	-2.819968000
C	2.921442000	-5.266992000	-2.810467000
H	2.871305000	-5.880820000	-3.702172000
C	3.463848000	-5.780184000	-1.629826000
H	3.831815000	-6.801237000	-1.614033000
C	3.539420000	-4.986469000	-0.482847000
H	3.971167000	-5.386970000	0.428554000
C	3.055661000	-3.678614000	-0.512480000
H	3.107413000	-3.056821000	0.374731000
C	2.494706000	-3.150386000	-1.679130000
H	2.088195000	-2.145156000	-1.679806000
C	0.432282000	-6.527109000	0.921113000
C	-0.008186000	-6.190614000	-0.362962000
C	-1.341474000	-5.814967000	-0.548379000
C	-2.246113000	-5.786761000	0.519295000
C	-1.795190000	-6.119780000	1.803168000
C	-0.458077000	-6.489145000	2.002702000
H	1.467939000	-6.808437000	1.076072000
H	0.665662000	-6.216815000	-1.211555000
H	-3.283620000	-5.512122000	0.355424000
H	-2.485217000	-6.097899000	2.639426000
H	-0.112781000	-6.748803000	2.997355000
S	-1.914102000	-5.391053000	-2.217655000
C	-1.490099000	-3.610667000	-2.140144000
H	-0.427427000	-3.502830000	-1.916908000
H	-1.710964000	-3.182639000	-3.119007000
H	-2.097132000	-3.126323000	-1.371895000

D. C-H bond activation 9,10-dihydroanthracene by complex 1.

$^{5}RC_{H}$

Mn	0.237885000	0.522262000	-0.170146000
N	-0.069259000	0.644923000	2.172514000
C	1.191468000	0.883585000	3.026444000
C	1.674131000	2.328487000	2.792772000
H	1.819299000	2.515238000	1.726021000
H	0.992511000	3.078532000	3.200992000
H	2.638119000	2.457548000	3.293661000
C	0.883254000	0.653303000	4.521273000
H	0.677450000	-0.400125000	4.733271000
H	0.033408000	1.250057000	4.867383000
H	1.754683000	0.946136000	5.114202000
C	2.316860000	-0.061640000	2.573856000
H	3.145095000	0.011893000	3.284949000
H	2.682951000	0.239811000	1.593285000
H	2.012170000	-1.109464000	2.534476000
C	-0.696602000	-0.695454000	2.408014000
H	-1.188919000	-0.750770000	3.385110000
H	0.092681000	-1.445858000	2.394617000
C	-1.706229000	-1.003383000	1.328634000
C	-2.796272000	-1.852974000	1.505230000
H	-2.949540000	-2.347495000	2.455393000
C	-3.684260000	-2.042946000	0.440199000
H	-4.522917000	-2.719189000	0.549399000
C	-3.509971000	-1.332047000	-0.751897000
H	-4.214010000	-1.430755000	-1.567927000
C	-2.419468000	-0.470577000	-0.874129000
N	-1.525706000	-0.376154000	0.139246000
C	-2.250852000	0.480336000	-2.032697000
H	-2.737665000	0.070973000	-2.918276000
H	-2.805448000	1.390649000	-1.777721000
N	-0.824142000	0.857047000	-2.314403000

C	-0.253511000	0.109814000	-3.535475000
C	-1.030132000	0.451954000	-4.826813000
H	-2.073200000	0.125063000	-4.794565000
H	-0.553648000	-0.064716000	-5.665837000
H	-1.005982000	1.524052000	-5.042701000
C	1.223066000	0.511058000	-3.731628000
H	1.318328000	1.458084000	-4.271539000
H	1.725405000	-0.254350000	-4.329688000
H	1.744658000	0.605820000	-2.779038000
C	-0.359123000	-1.396827000	-3.250596000
H	0.154678000	-1.656106000	-2.324437000
H	0.093881000	-1.952131000	-4.076907000
H	-1.399036000	-1.725396000	-3.171397000
C	-0.665311000	2.342758000	-2.431689000
H	-1.311255000	2.765484000	-3.209248000
H	0.367328000	2.546765000	-2.719336000
C	-0.971053000	3.018676000	-1.119876000
C	-1.512398000	4.298591000	-1.023276000
H	-1.708450000	4.870376000	-1.921025000
C	-1.810091000	4.813951000	0.243172000
H	-2.215847000	5.813302000	0.340578000
C	-1.625767000	4.018365000	1.377752000
H	-1.903891000	4.375034000	2.361187000
C	-1.103842000	2.733417000	1.227774000
N	-0.733159000	2.292835000	0.001559000
C	-1.085428000	1.731042000	2.352229000
H	-0.969474000	2.237831000	3.311350000
H	-2.079936000	1.271600000	2.373412000
O	1.915791000	1.180667000	-0.502349000
H	2.053473000	2.112161000	-0.717969000
O	0.993104000	-1.235296000	-0.221113000
I	2.829601000	-1.377197000	-0.830697000
C	2.862700000	-3.465350000	-0.918618000
C	2.260352000	-4.108180000	-2.007380000
H	1.771895000	-3.543847000	-2.791447000
C	2.288133000	-5.499394000	-2.046214000
H	1.802205000	-6.018529000	-2.863901000
C	2.913763000	-6.219224000	-1.024696000
H	2.918383000	-7.303576000	-1.058098000
C	3.519929000	-5.558927000	0.044707000
H	3.980729000	-6.120287000	0.848846000
C	3.494614000	-4.166696000	0.113181000
H	3.935187000	-3.649563000	0.956041000
C	3.020994000	-5.124265000	3.760289000
C	2.045839000	-4.379466000	3.090171000
C	1.071985000	-5.010259000	2.303064000
C	1.073882000	-6.412139000	2.192669000
C	2.057265000	-7.155258000	2.861838000
C	3.028402000	-6.520178000	3.642987000
C	0.039698000	-4.197842000	1.551353000
C	0.018770000	-7.096960000	1.348559000
C	-0.421461000	-6.260163000	0.164851000
C	-0.423803000	-4.858376000	0.270536000
C	-0.851795000	-4.083713000	-0.816638000
H	-0.822185000	-3.004300000	-0.735930000
C	-1.283173000	-4.688628000	-2.000430000
C	-1.272140000	-6.084477000	-2.110371000
C	-0.838016000	-6.861532000	-1.031863000
H	0.450767000	-3.212436000	1.310929000
H	3.771999000	-4.621119000	4.360256000
H	2.048508000	-3.296070000	3.167327000
H	2.064454000	-8.236767000	2.762102000
H	3.785824000	-7.107525000	4.151386000
H	-0.860047000	-7.311617000	1.979759000
H	-1.611269000	-4.078585000	-2.834936000
H	-1.592859000	-6.563152000	-3.029840000
H	-0.819832000	-7.944290000	-1.116157000
H	0.385446000	-8.070705000	1.006063000
H	-0.831130000	-4.023979000	2.205268000

^{3}RC$_{H}$

Mn	0.206492000	0.569756000	-0.155484000
N	-0.081875000	0.631826000	2.116049000
C	1.183340000	0.879624000	2.979188000
C	1.651336000	2.329366000	2.752558000
H	1.807938000	2.516815000	1.688015000
H	0.958493000	3.071951000	3.155190000
H	2.608020000	2.465639000	3.265213000
C	0.859065000	0.639574000	4.469645000
H	0.670554000	-0.417398000	4.677627000
H	0.000070000	1.222430000	4.815038000
H	1.723218000	0.944750000	5.066490000
C	2.318421000	-0.058913000	2.540424000
H	3.135278000	0.027478000	3.263092000
H	2.691621000	0.240409000	1.562616000
H	2.021303000	-1.108578000	2.502826000
C	-0.703739000	-0.719618000	2.361082000
H	-1.172571000	-0.764972000	3.348617000
H	0.091538000	-1.461030000	2.329003000
C	-1.725262000	-1.021898000	1.293183000
C	-2.809123000	-1.880200000	1.457557000
H	-2.963367000	-2.388875000	2.400074000
C	-3.689366000	-2.065720000	0.384151000
H	-4.523581000	-2.749109000	0.482035000
C	-3.511048000	-1.347330000	-0.802643000
H	-4.205708000	-1.450835000	-1.626066000
C	-2.427053000	-0.474652000	-0.908387000
N	-1.542686000	-0.376218000	0.112670000
C	-2.241856000	0.480993000	-2.058565000
H	-2.691203000	0.070917000	-2.962364000
H	-2.809501000	1.386858000	-1.819771000
N	-0.804336000	0.879833000	-2.306373000
C	-0.194958000	0.134315000	-3.515161000
C	-0.957171000	0.458484000	-4.819679000
H	-1.991920000	0.106030000	-4.809498000
H	-0.449997000	-0.048905000	-5.646114000
H	-0.952741000	1.529930000	-5.038872000
C	1.274732000	0.566538000	-3.688071000
H	1.356892000	1.511913000	-4.232827000
H	1.802744000	-0.191272000	-4.273291000
H	1.777221000	0.677128000	-2.727082000
C	-0.285029000	-1.370409000	-3.218844000
H	0.195819000	-1.610542000	-2.270044000
H	0.209479000	-1.924687000	-4.021438000
H	-1.321458000	-1.715930000	-3.177570000
C	-0.675265000	2.375017000	-2.429357000
H	-1.317530000	2.768999000	-3.223562000
H	0.357600000	2.598367000	-2.699296000
C	-1.020598000	3.040606000	-1.123321000
C	-1.596892000	4.304050000	-1.019766000
H	-1.801552000	4.880268000	-1.912624000

C	-1.916100000	4.799758000	0.250030000
H	-2.348124000	5.787415000	0.352939000
C	-1.717160000	4.001560000	1.380606000
H	-2.007078000	4.345607000	2.365001000
C	-1.164884000	2.730823000	1.221540000
N	-0.775655000	2.306996000	-0.006575000
C	-1.118273000	1.712954000	2.327605000
H	-0.984718000	2.196320000	3.294926000
H	-2.102285000	1.233847000	2.358325000
O	1.896954000	1.224401000	-0.472431000
H	2.003280000	2.146339000	-0.744104000
O	1.042017000	-1.197775000	-0.145490000
I	2.851513000	-1.389454000	-0.859284000
C	2.851490000	-3.480557000	-0.952878000
C	2.226493000	-4.113145000	-2.034201000
H	1.738013000	-3.540438000	-2.811866000
C	2.230940000	-5.504921000	-2.075043000
H	1.727730000	-6.014718000	-2.888346000
C	2.854128000	-6.236584000	-1.060802000
H	2.839135000	-7.320853000	-1.094360000
C	3.483303000	-5.586850000	0.001589000
H	3.943391000	-6.155969000	0.800910000
C	3.484660000	-4.194111000	0.068966000
H	3.946084000	-3.685420000	0.905776000
C	3.130777000	-5.215864000	3.742833000
C	2.168268000	-4.435138000	3.095700000
C	1.151567000	-5.029686000	2.334774000
C	1.095995000	-6.430943000	2.229571000
C	2.067478000	-7.210065000	2.874780000
C	3.082282000	-6.611234000	3.628575000
C	0.136962000	-4.179100000	1.601458000
C	-0.008279000	-7.076130000	1.417663000
C	-0.445579000	-6.228201000	0.240768000
C	-0.387025000	-4.826986000	0.337541000
C	-0.812516000	-4.040664000	-0.742263000
H	-0.730442000	-2.963326000	-0.669921000
C	-1.304177000	-4.633426000	-1.908490000
C	-1.354163000	-6.029017000	-2.009941000
C	-0.920503000	-6.817959000	-0.939859000
H	0.584309000	-3.214246000	1.342650000
H	3.915597000	-4.740963000	4.322224000
H	2.215795000	-3.352329000	3.168407000
H	2.030495000	-8.291248000	2.778215000
H	3.829993000	-7.226207000	4.118371000
H	-0.877965000	-7.250496000	2.073535000
H	-1.631373000	-4.014690000	-2.736980000
H	-1.721702000	-6.498727000	-2.916460000
H	-0.949385000	-7.900991000	-1.017833000
H	0.308120000	-8.066313000	1.072148000
H	-0.709674000	-3.962792000	2.274656000

^{5}TS1$_H$

Mn	0.230970000	0.030899000	-0.092727000
N	0.224781000	0.594826000	2.290714000
C	1.548607000	0.480563000	3.090232000
C	2.627424000	1.319496000	2.383411000
H	2.836355000	0.924811000	1.389824000
H	2.353844000	2.374202000	2.287096000
H	3.548132000	1.277161000	2.972723000
C	1.357184000	0.978397000	4.541015000
H	0.558913000	0.433060000	5.054090000
H	1.138252000	2.048505000	4.592562000
H	2.283806000	0.805390000	5.096888000
C	2.008479000	-0.990311000	3.127343000
H	3.064684000	-1.017410000	3.410913000
H	1.896264000	-1.465086000	2.151832000
H	1.464276000	-1.573426000	3.875448000
C	-0.788529000	-0.408710000	2.751057000
H	-1.149667000	-0.182804000	3.760077000
H	-0.298570000	-1.380278000	2.784173000
C	-1.957936000	-0.484026000	1.809641000
C	-3.278422000	-0.509046000	2.247094000
H	-3.493015000	-0.494679000	3.307410000
C	-4.310007000	-0.513722000	1.299923000
H	-5.344383000	-0.516335000	1.620057000
C	-3.993579000	-0.483282000	-0.057537000
H	-4.770092000	-0.457325000	-0.811069000
C	-2.652056000	-0.478971000	-0.451423000
N	-1.666720000	-0.503036000	0.478820000
C	-2.282546000	-0.488610000	-1.910864000
H	-2.352742000	-1.525863000	-2.242082000
H	-3.038091000	0.072673000	-2.472980000
N	-0.907986000	0.016597000	-2.211564000
C	-0.278931000	-0.755261000	-3.402431000
C	-1.212364000	-0.711468000	-4.633144000
H	-2.163289000	-1.220366000	-4.451711000
H	-0.718988000	-1.223125000	-5.465468000
H	-1.418568000	0.314679000	-4.952967000
C	1.075963000	-0.123929000	-3.779509000
H	0.954417000	0.793006000	-4.364370000
H	1.625906000	-0.832642000	-4.405247000
H	1.671771000	0.100347000	-2.894423000
C	-0.055205000	-2.213022000	-2.971527000
H	0.535743000	-2.261411000	-2.056667000
H	0.477347000	-2.741447000	-3.768051000
H	-0.990453000	-2.750197000	-2.806929000
C	-0.929620000	1.493737000	-2.463854000
H	-1.626707000	1.752754000	-3.267938000
H	0.072661000	1.788999000	-2.774036000
C	-1.282742000	2.274062000	-1.229144000
C	-2.127854000	3.378840000	-1.250740000
H	-2.580024000	3.691051000	-2.182946000
C	-2.393315000	4.055344000	-0.054355000
H	-3.054534000	4.913012000	-0.048081000
C	-1.820424000	3.601290000	1.132998000
H	-2.027736000	4.090829000	2.076062000
C	-0.981387000	2.482977000	1.105840000
N	-0.718209000	1.858616000	-0.063493000
C	-0.339400000	1.977563000	2.373379000
H	0.466925000	2.669984000	2.618151000
H	-1.065114000	2.051116000	3.192158000
O	1.768904000	0.773533000	-0.772200000
H	1.943347000	1.683202000	-0.494150000
O	1.057172000	-1.714746000	0.065849000
I	3.245506000	-1.409158000	-0.584113000
C	3.964273000	-3.304590000	-0.100122000
C	4.137211000	-4.259272000	-1.114351000
H	3.802114000	-4.057022000	-2.125576000
C	4.760419000	-5.464763000	-0.802967000
H	4.906928000	-6.208177000	-1.579344000
C	5.190081000	-5.716564000	0.503594000
H	5.672058000	-6.659356000	0.739957000
C	4.993782000	-4.766104000	1.508890000
H	5.316735000	-4.970917000	2.523683000
C	4.378927000	-3.552445000	1.215683000
H	4.233678000	-2.808413000	1.989532000
C	1.888647000	-5.531033000	3.182945000
C	0.986579000	-4.562403000	2.747154000
C	0.565529000	-4.532125000	1.401838000

C	1.108575000	-5.459251000	0.477186000
C	1.994889000	-6.437094000	0.931408000
C	2.384219000	-6.478879000	2.275400000
C	-0.476678000	-3.597303000	0.942253000
C	0.684213000	-5.373340000	-0.971600000
C	-0.760235000	-4.955562000	-1.128982000
C	-1.318081000	-4.072592000	-0.175539000
C	-2.661158000	-3.675135000	-0.299810000
H	-3.098141000	-3.042546000	0.462895000
C	-3.433892000	-4.110078000	-1.377331000
C	-2.872196000	-4.969492000	-2.333300000
C	-1.546328000	-5.400026000	-2.196114000
H	0.205633000	-2.614594000	0.482951000
H	2.195383000	-5.559169000	4.222397000
H	0.571297000	-3.848626000	3.450954000
H	2.388992000	-7.165523000	0.231662000
H	3.073182000	-7.244851000	2.612878000
H	0.870389000	-6.321502000	-1.484951000
H	-4.467463000	-3.794333000	-1.465697000
H	-3.468441000	-5.314389000	-3.170798000
H	-1.119624000	-6.080869000	-2.925606000
H	1.318116000	-4.626073000	-1.476584000
H	-1.045861000	-3.139192000	1.751535000

$^{3}TS1_{H}$

Mn	0.103052000	0.278649000	0.052275000
N	-0.117006000	0.573555000	2.201235000
C	1.159973000	0.787404000	3.082318000
C	1.745467000	2.179022000	2.776497000
H	1.918197000	2.295367000	1.705614000
H	1.122517000	3.000164000	3.138040000
H	2.711195000	2.257966000	3.284367000
C	0.790960000	0.680651000	4.579213000
H	0.551549000	-0.345598000	4.869849000
H	-0.042718000	1.327870000	4.865757000
H	1.660364000	0.991795000	5.165019000
C	2.229388000	-0.261590000	2.749699000
H	2.984851000	-0.247010000	3.541061000
H	2.715417000	-0.006896000	1.811615000
H	1.840030000	-1.277143000	2.683872000
C	-0.880920000	-0.667599000	2.603410000
H	-1.288837000	-0.576434000	3.612501000
H	-0.183485000	-1.500341000	2.585889000
C	-1.987663000	-0.883618000	1.608756000
C	-3.242619000	-1.416743000	1.880684000
H	-3.481817000	-1.770043000	2.874871000
C	-4.189749000	-1.465314000	0.846029000
H	-5.171270000	-1.882074000	1.033609000
C	-3.879610000	-0.954437000	-0.418061000
H	-4.610246000	-0.957779000	-1.216179000
C	-2.606094000	-0.421787000	-0.635233000
N	-1.698071000	-0.434074000	0.361481000
C	-2.201747000	0.288486000	-1.892804000
H	-2.621361000	-0.218330000	-2.758797000
H	-2.658818000	1.283490000	-1.872633000
N	-0.694875000	0.455250000	-2.049278000
C	-0.159664000	-0.456608000	-3.201731000
C	-0.904458000	-0.196489000	-4.533785000
H	-1.945576000	-0.527841000	-4.523072000
H	-0.394747000	-0.773946000	-5.311251000
H	-0.870822000	0.853999000	-4.834676000
C	1.332045000	-0.144489000	-3.436638000
H	1.461791000	0.699627000	-4.120551000
H	1.810725000	-1.011504000	-3.899525000
H	1.851749000	0.081923000	-2.508516000
C	-0.379733000	-1.911752000	-2.777053000
H	0.104102000	-2.132437000	-1.826440000
H	0.020024000	-2.576667000	-3.548626000
H	-1.440777000	-2.147820000	-2.674743000
C	-0.386314000	1.905459000	-2.355169000
H	-0.941491000	2.250686000	-3.231124000
H	0.675859000	1.982066000	-2.584804000
C	-0.714387000	2.751860000	-1.164692000
C	-1.108081000	4.086175000	-1.207012000
H	-1.189403000	4.599102000	-2.156230000
C	-1.403622000	4.737365000	-0.002651000
H	-1.698242000	5.779354000	-0.009432000
C	-1.351080000	4.033995000	1.205664000
H	-1.614731000	4.509752000	2.141208000
C	-0.973940000	2.692318000	1.184736000
N	-0.624206000	2.102684000	0.019621000
C	-1.046213000	1.769167000	2.357295000
H	-0.854374000	2.292026000	3.291941000
H	-2.074614000	1.400004000	2.420058000
O	1.836019000	0.805153000	-0.235448000
H	2.030951000	1.695933000	-0.553594000
O	0.885320000	-1.655030000	0.180797000
I	2.796444000	-1.639632000	-0.484750000
C	3.102616000	-3.680956000	-0.812151000
C	2.734576000	-4.229266000	-2.049852000
H	2.142931000	-3.660937000	-2.756624000
C	3.160382000	-5.516895000	-2.361994000
H	2.881942000	-5.957199000	-3.313347000
C	3.949708000	-6.235154000	-1.455728000
H	4.289492000	-7.233155000	-1.711966000
C	4.295948000	-5.680478000	-0.222061000
H	4.887826000	-6.247957000	0.487281000
C	3.863674000	-4.398608000	0.117937000
H	4.111903000	-3.972029000	1.082445000
C	2.177841000	-4.538726000	3.954519000
C	1.211052000	-3.918073000	3.165210000
C	0.767372000	-4.480817000	1.950258000
C	1.299439000	-5.726903000	1.560138000
C	2.260015000	-6.357204000	2.371457000
C	2.713583000	-5.770980000	3.552026000
C	-0.234772000	-3.750514000	1.108122000
C	0.857489000	-6.422967000	0.295490000
C	-0.279258000	-5.762820000	-0.446183000
C	-0.822776000	-4.533377000	-0.022669000
C	-1.948519000	-4.028618000	-0.700867000
H	-2.385596000	-3.100734000	-0.359928000
C	-2.505989000	-4.690837000	-1.792624000
C	-1.936142000	-5.893001000	-2.236603000
C	-0.837650000	-6.420426000	-1.556450000
H	0.310898000	-2.738206000	0.647249000
H	2.501005000	-4.075294000	4.880823000
H	0.773569000	-2.988023000	3.509247000
H	2.657031000	-7.318779000	2.058533000
H	3.463087000	-6.269002000	4.157845000
H	0.567497000	-7.455133000	0.541617000

H	-3.373405000	-4.274418000	-2.294734000
H	-2.352819000	-6.416470000	-3.090704000
H	-0.406503000	-7.365209000	-1.876600000
H	1.719265000	-6.528731000	-0.375590000
H	-1.037830000	-3.348132000	1.737217000

^{5}IM1$_{H}$

Mn	-0.253037000	-0.125941000	-0.384446000
N	-0.596536000	0.222006000	1.981147000
C	0.577244000	-0.173024000	2.893570000
C	1.790177000	0.697159000	2.521078000
H	2.095971000	0.491793000	1.494789000
H	1.587529000	1.766594000	2.630192000
H	2.621677000	0.451271000	3.188479000
C	0.220276000	0.023485000	4.383973000
H	-0.654270000	-0.568590000	4.670880000
H	0.030621000	1.070597000	4.638531000
H	1.063105000	-0.313830000	4.995525000
C	0.928839000	-1.654368000	2.653992000
H	1.925317000	-1.855074000	3.057923000
H	0.927219000	-1.897959000	1.590580000
H	0.233120000	-2.320245000	3.173096000
C	-1.794506000	-0.655430000	2.166196000
H	-2.284561000	-0.491269000	3.132982000
H	-1.452872000	-1.690586000	2.141894000
C	-2.796633000	-0.438667000	1.060606000
C	-4.172933000	-0.574381000	1.230532000
H	-4.573557000	-0.854926000	2.195885000
C	-5.015321000	-0.333468000	0.138993000
H	-6.086738000	-0.452699000	0.243474000
C	-4.473075000	0.097873000	-1.074807000
H	-5.110176000	0.337012000	-1.916778000
C	-3.088850000	0.245732000	-1.187040000
N	-2.288592000	-0.078395000	-0.144768000
C	-2.440189000	0.896695000	-2.384584000
H	-3.022005000	0.687240000	-3.284137000
H	-2.512836000	1.979408000	-2.229404000
N	-0.999082000	0.537639000	-2.558157000
C	-0.775105000	-0.492862000	-3.677697000
C	-1.057849000	0.133699000	-5.060524000
H	-2.071712000	0.540247000	-5.133835000
H	-0.955089000	-0.636379000	-5.831215000
H	-0.346828000	0.932178000	-5.293307000
C	0.674093000	-1.010984000	-3.630183000
H	1.407654000	-0.228421000	-3.841795000
H	0.788348000	-1.780504000	-4.400844000
H	0.896860000	-1.447181000	-2.656947000
C	-1.713830000	-1.690996000	-3.435200000
H	-1.503323000	-2.138676000	-2.463405000
H	-1.526486000	-2.443946000	-4.206660000
H	-2.772446000	-1.424873000	-3.489996000
C	-0.153643000	1.767546000	-2.698836000
H	-0.465201000	2.389407000	-3.545514000
H	0.873503000	1.445167000	-2.875983000
C	-0.211189000	2.591097000	-1.433850000
C	-0.150388000	3.982265000	-1.410386000
H	-0.038055000	4.535211000	-2.334077000
C	-0.250945000	4.642475000	-0.179289000
H	-0.190489000	5.723147000	-0.137346000
C	-0.466629000	3.907867000	0.989505000
H	-0.592313000	4.402937000	1.944199000
C	-0.545138000	2.514719000	0.912531000
N	-0.366062000	1.897419000	-0.276529000
C	-0.960516000	1.669508000	2.094327000
H	-0.564760000	2.109177000	3.011381000
H	-2.051214000	1.750960000	2.172880000
O	1.570067000	-0.061833000	-0.692614000
H	1.960306000	0.813121000	-0.568635000
O	-0.249958000	-1.967311000	-0.427388000
I	3.502732000	-2.106606000	-0.433266000
C	5.053198000	-3.543186000	-0.246766000
C	5.333944000	-4.410339000	-1.307891000
H	4.778777000	-4.337518000	-2.237072000
C	6.325074000	-5.384089000	-1.160123000
H	6.536709000	-6.059353000	-1.983551000
C	7.036869000	-5.491800000	0.037337000
H	7.804617000	-6.250679000	0.148474000
C	6.755416000	-4.618657000	1.090453000
H	7.302057000	-4.696637000	2.025257000
C	5.763651000	-3.644343000	0.954107000
H	5.539483000	-2.978579000	1.780619000
C	3.337181000	-5.995687000	2.529400000
C	2.111586000	-5.620957000	3.042972000
C	1.032552000	-5.314445000	2.160757000
C	1.225189000	-5.376556000	0.741033000
C	2.471895000	-5.766135000	0.251007000
C	3.512220000	-6.075173000	1.129941000
C	-0.230546000	-4.970788000	2.670048000
C	0.124197000	-4.968986000	-0.180636000
C	-1.216450000	-4.735142000	0.437648000
C	-1.352871000	-4.688950000	1.859257000
C	-2.608042000	-4.359307000	2.446441000
H	-2.691522000	-4.327987000	3.526965000
C	-3.697914000	-4.077870000	1.641893000
C	-3.560212000	-4.133256000	0.238350000
C	-2.337685000	-4.459854000	-0.354467000
H	-1.159189000	-2.303094000	-0.389450000
H	4.164237000	-6.227411000	3.188508000
H	1.949253000	-5.557792000	4.113120000
H	2.638766000	-5.814423000	-0.818254000
H	4.474523000	-6.369410000	0.729857000
H	0.051609000	-5.654315000	-1.033763000
H	-4.652956000	-3.819582000	2.081231000
H	-4.415165000	-3.913113000	-0.390334000
H	-2.253128000	-4.497756000	-1.434875000
H	0.413503000	-3.987266000	-0.614584000
H	-0.357051000	-4.931533000	3.748615000

^{3}IM1$_{H}$

Mn	-0.194963000	-0.060091000	-0.465909000
N	-0.731854000	0.110577000	1.771538000
C	0.389106000	-0.186823000	2.799461000
C	1.493542000	0.868423000	2.616034000
H	1.864519000	0.839228000	1.588846000
H	1.146502000	1.878597000	2.853217000
H	2.314079000	0.637958000	3.302748000
C	-0.159674000	-0.147622000	4.243489000
H	-0.933592000	-0.903411000	4.407085000
H	-0.560976000	0.830720000	4.522563000
H	0.664752000	-0.368024000	4.928592000
C	0.963502000	-1.587963000	2.532876000
H	1.896111000	-1.700260000	3.093074000
H	1.169137000	-1.740611000	1.475781000
H	0.285154000	-2.372221000	2.877015000
C	-1.859570000	-0.890025000	1.842508000
H	-2.416596000	-0.806207000	2.780329000
H	-1.416103000	-1.883734000	1.794626000
C	-2.783509000	-0.703041000	0.668610000
C	-4.155986000	-0.940256000	0.680163000
H	-4.642413000	-1.284198000	1.582951000

C	-4.886690000	-0.714226000	-0.493798000
H	-5.951832000	-0.909937000	-0.512758000
C	-4.250161000	-0.205173000	-1.630519000
H	-4.808069000	0.009913000	-2.532955000
C	-2.878741000	0.052796000	-1.573024000
N	-2.184338000	-0.241291000	-0.453927000
C	-2.124144000	0.784332000	-2.649607000
H	-2.516568000	0.529729000	-3.633353000
H	-2.320410000	1.852813000	-2.511013000
N	-0.619572000	0.575454000	-2.601071000
C	-0.150259000	-0.386252000	-3.729270000
C	-0.381818000	0.245633000	-5.119409000
H	-1.430176000	0.494149000	-5.306778000
H	-0.078506000	-0.479790000	-5.880232000
H	0.221638000	1.146107000	-5.265077000
C	1.350311000	-0.680984000	-3.564529000
H	1.974189000	0.186133000	-3.802004000
H	1.625762000	-1.474952000	-4.264900000
H	1.585478000	-1.011306000	-2.554908000
C	-0.943575000	-1.699185000	-3.597990000
H	-0.807515000	-2.122258000	-2.600319000
H	-0.566612000	-2.408643000	-4.340844000
H	-2.011335000	-1.564035000	-3.792150000
C	0.065086000	1.920429000	-2.671463000
H	-0.277327000	2.493897000	-3.538322000
H	1.135632000	1.751447000	-2.795592000
C	-0.199130000	2.693174000	-1.404233000
C	-0.215527000	4.082231000	-1.308346000
H	-0.020216000	4.691839000	-2.181071000
C	-0.496316000	4.666093000	-0.065967000
H	-0.499497000	5.744365000	0.035882000
C	-0.802218000	3.859533000	1.034594000
H	-1.056294000	4.295340000	1.992286000
C	-0.796661000	2.472062000	0.876659000
N	-0.456885000	1.930823000	-0.313741000
C	-1.275265000	1.514273000	1.933625000
H	-1.054611000	1.904336000	2.926713000
H	-2.367195000	1.468109000	1.861090000
O	1.693280000	0.110817000	-0.416617000
H	2.040127000	0.763272000	-1.037809000
O	-0.037085000	-1.908395000	-0.559334000
I	3.705329000	-1.868330000	-0.392291000
C	5.080007000	-3.484508000	-0.460451000
C	5.249008000	-4.195815000	-1.652728000
H	4.721749000	-3.894015000	-2.551397000
C	6.088825000	-5.312095000	-1.674553000
H	6.215541000	-5.867324000	-2.598850000
C	6.757393000	-5.715089000	-0.515703000
H	7.404229000	-6.586219000	-0.535526000
C	6.593077000	-4.991169000	0.667159000
H	7.110688000	-5.297230000	1.571153000
C	5.753716000	-3.874921000	0.700814000
H	5.612178000	-3.329121000	1.627319000
C	3.246358000	-5.934325000	1.947790000
C	2.216652000	-5.655553000	2.822552000
C	0.939823000	-5.263956000	2.319950000
C	0.730982000	-5.135501000	0.906352000
C	1.796739000	-5.417088000	0.044404000
C	3.030501000	-5.822000000	0.555093000
C	-0.132549000	-5.029756000	3.197950000
C	-0.580917000	-4.660115000	0.390116000
C	-1.698660000	-4.571477000	1.367474000
C	-1.435723000	-4.698984000	2.769625000
C	-2.487744000	-4.521131000	3.713423000
H	-2.266144000	-4.609198000	4.771053000
C	-3.770355000	-4.247691000	3.276375000
C	-4.028028000	-4.145595000	1.891385000
C	-3.006539000	-4.293586000	0.952243000
H	0.887874000	-2.107394000	-0.787187000
H	4.219191000	-6.233985000	2.315410000
H	2.356473000	-5.735938000	3.894710000
H	1.658972000	-5.323863000	-1.026666000
H	3.842321000	-6.046957000	-0.125220000
H	-0.878711000	-5.210825000	-0.510743000
H	-4.576842000	-4.116116000	3.986723000
H	-5.037654000	-3.945305000	1.551436000
H	-3.223172000	-4.193764000	-0.105120000
H	-0.420868000	-3.607409000	0.013257000
H	0.045972000	-5.128013000	4.265305000

$^5TS2_H$

Mn	-0.223130000	-0.170133000	-0.353694000
N	-0.630940000	0.166241000	1.966637000
C	0.529854000	-0.186071000	2.916989000
C	1.687839000	0.796490000	2.663765000
H	2.038919000	0.711281000	1.634900000
H	1.417878000	1.835557000	2.868869000
H	2.516863000	0.537901000	3.328963000
C	0.076715000	-0.106683000	4.391044000
H	-0.705396000	-0.838768000	4.613075000
H	-0.290207000	0.887588000	4.663577000
H	0.931056000	-0.329850000	5.037227000
C	1.023507000	-1.610102000	2.614835000
H	1.919222000	-1.806491000	3.211824000
H	1.283307000	-1.728436000	1.562829000
H	0.287206000	-2.367346000	2.888854000
C	-1.816890000	-0.739298000	2.126047000
H	-2.334724000	-0.576974000	3.077306000
H	-1.456861000	-1.768718000	2.117349000
C	-2.788264000	-0.534847000	0.990293000
C	-4.166852000	-0.691844000	1.108245000
H	-4.598088000	-0.991390000	2.054172000
C	-4.972547000	-0.448998000	-0.010616000
H	-6.045068000	-0.583797000	0.055073000
C	-4.395990000	0.002133000	-1.201581000
H	-5.007766000	0.240395000	-2.062121000
C	-3.011671000	0.168545000	-1.266175000
N	-2.245376000	-0.155028000	-0.196202000
C	-2.320798000	0.832068000	-2.430934000
H	-2.862818000	0.632729000	-3.356626000
H	-2.399138000	1.912793000	-2.266933000
N	-0.869502000	0.472952000	-2.546786000
C	-0.596745000	-0.547776000	-3.671016000
C	-0.767376000	0.115294000	-5.054866000
H	-1.751199000	0.581479000	-5.171393000
H	-0.669592000	-0.648446000	-5.832113000
H	0.000043000	0.872908000	-5.237783000
C	0.830627000	-1.106240000	-3.529632000
H	1.598249000	-0.333114000	-3.612920000
H	1.000591000	-1.823970000	-4.338755000
H	0.956042000	-1.618178000	-2.575812000

C	-1.583476000	-1.723031000	-3.531686000
H	-1.480979000	-2.198218000	-2.556261000
H	-1.341211000	-2.470422000	-4.292708000
H	-2.625259000	-1.431252000	-3.682925000
C	-0.030378000	1.712378000	-2.668025000
H	-0.332413000	2.328120000	-3.521580000
H	1.003495000	1.402271000	-2.827373000
C	-0.128801000	2.537492000	-1.407198000
C	-0.057541000	3.927947000	-1.377735000
H	0.099271000	4.481734000	-2.294319000
C	-0.207010000	4.586168000	-0.150511000
H	-0.139769000	5.666119000	-0.103993000
C	-0.480903000	3.852223000	1.007289000
H	-0.646594000	4.347008000	1.955806000
C	-0.565297000	2.460969000	0.924040000
N	-0.337783000	1.846687000	-0.258182000
C	-1.042156000	1.603784000	2.071367000
H	-0.727102000	2.042636000	3.019112000
H	-2.136945000	1.654414000	2.068320000
O	1.584178000	-0.115881000	-0.567586000
H	1.981031000	0.760665000	-0.476756000
O	-0.317594000	-2.100916000	-0.417348000
I	3.643016000	-2.038512000	-0.581140000
C	5.162138000	-3.512526000	-0.494137000
C	5.291472000	-4.437518000	-1.534778000
H	4.655232000	-4.371392000	-2.410856000
C	6.233854000	-5.463679000	-1.429225000
H	6.328399000	-6.186150000	-2.234105000
C	7.044436000	-5.564501000	-0.295950000
H	7.771642000	-6.366073000	-0.216262000
C	6.915881000	-4.629447000	0.733518000
H	7.542057000	-4.701069000	1.617533000
C	5.974606000	-3.601711000	0.640373000
H	5.864920000	-2.889529000	1.451024000
C	3.267767000	-5.774711000	2.507933000
C	2.059723000	-5.473680000	3.094785000
C	0.928419000	-5.148695000	2.283620000
C	1.070944000	-5.088409000	0.850439000
C	2.324977000	-5.403970000	0.284296000
C	3.392574000	-5.756782000	1.092510000
C	-0.333866000	-4.921595000	2.853527000
C	-0.061218000	-4.678173000	0.044400000
C	-1.381230000	-4.642106000	0.655401000
C	-1.489868000	-4.670220000	2.087360000
C	-2.760012000	-4.457612000	2.704260000
H	-2.823873000	-4.472684000	3.786718000
C	-3.879244000	-4.230476000	1.932126000
C	-3.772682000	-4.226760000	0.516571000
C	-2.551985000	-4.428595000	-0.109402000
H	-1.257165000	-2.309988000	-0.547296000
H	4.126658000	-6.032437000	3.115823000
H	1.941170000	-5.490905000	4.172703000
H	2.443862000	-5.373130000	-0.792177000
H	4.341780000	-6.017379000	0.641543000
H	-0.030596000	-4.985896000	-1.002942000
H	-4.842386000	-4.060433000	2.397494000
H	-4.661205000	-4.061891000	-0.082110000
H	-2.487983000	-4.432707000	-1.192753000
H	0.062557000	-3.385254000	-0.132507000
H	-0.429009000	-4.956894000	3.935240000

$^{3}TS2_{H}$

Mn	-0.287572000	-0.079145000	-0.402035000
N	-0.737493000	0.035426000	1.771399000
C	0.411998000	-0.351150000	2.747703000
C	1.502597000	0.733205000	2.697056000
H	1.872970000	0.862292000	1.680300000
H	1.170338000	1.698004000	3.086613000
H	2.339396000	0.403141000	3.319112000
C	-0.134759000	-0.489315000	4.184831000
H	-0.801685000	-1.349823000	4.285810000
H	-0.663503000	0.406137000	4.525408000
H	0.709963000	-0.648239000	4.861059000
C	1.020610000	-1.683755000	2.299678000
H	1.806803000	-1.968680000	3.004863000
H	1.463368000	-1.598469000	1.309008000
H	0.286232000	-2.486892000	2.305719000
C	-1.933887000	-0.886868000	1.900582000
H	-2.440356000	-0.743899000	2.857979000
H	-1.574124000	-1.914389000	1.859612000
C	-2.883450000	-0.628714000	0.761198000
C	-4.263583000	-0.803131000	0.806232000
H	-4.738405000	-1.159892000	1.710181000
C	-5.015579000	-0.500460000	-0.336171000
H	-6.088960000	-0.643358000	-0.328054000
C	-4.389611000	0.014716000	-1.477015000
H	-4.964026000	0.287332000	-2.352775000
C	-3.007148000	0.198303000	-1.462482000
N	-2.293475000	-0.165928000	-0.370259000
C	-2.236650000	0.913802000	-2.537479000
H	-2.689363000	0.759378000	-3.516400000
H	-2.318263000	1.986001000	-2.331094000
N	-0.765008000	0.547610000	-2.553671000
C	-0.426338000	-0.427076000	-3.715886000
C	-0.470486000	0.319143000	-5.067322000
H	-1.418477000	0.843653000	-5.224671000
H	-0.362522000	-0.412831000	-5.872892000
H	0.347746000	1.037376000	-5.164606000
C	0.969791000	-1.026650000	-3.490216000
H	1.749865000	-0.265794000	-3.418099000
H	1.211165000	-1.667993000	-4.343992000
H	0.988089000	-1.626647000	-2.581586000
C	-1.455194000	-1.572860000	-3.734399000
H	-1.503174000	-2.080597000	-2.772367000
H	-1.134054000	-2.308769000	-4.476702000
H	-2.459405000	-1.246995000	-4.013550000
C	0.060651000	1.816284000	-2.620169000
H	-0.223092000	2.427572000	-3.480794000
H	1.105339000	1.525009000	-2.735709000
C	-0.120973000	2.606929000	-1.349886000
C	-0.057178000	3.993946000	-1.248875000
H	0.151970000	4.593921000	-2.124749000
C	-0.280511000	4.589532000	-0.000258000
H	-0.222741000	5.666103000	0.101206000
C	-0.610692000	3.802224000	1.108724000
H	-0.824997000	4.252409000	2.069177000
C	-0.681003000	2.418503000	0.953139000
N	-0.392665000	1.863119000	-0.247174000
C	-1.194290000	1.468827000	1.998944000
H	-0.932139000	1.805305000	3.001001000
H	-2.287474000	1.487868000	1.945515000
O	1.514673000	-0.057536000	-0.546121000
H	1.957435000	0.787828000	-0.391244000
O	-0.310302000	-2.154151000	-0.617607000
I	3.626370000	-2.049196000	-0.768351000
C	5.109277000	-3.559716000	-0.722466000
C	5.082940000	-4.581415000	-1.676342000
H	4.343845000	-4.573820000	-2.470117000
C	6.005868000	-5.626573000	-1.587219000

H	5.980924000	-6.424020000	-2.323513000
C	6.949024000	-5.650260000	-0.557213000
H	7.660605000	-6.466787000	-0.489287000
C	6.973472000	-4.618968000	0.384253000
H	7.703663000	-4.630111000	1.187620000
C	6.054008000	-3.570365000	0.307671000
H	6.065294000	-2.781125000	1.051425000
C	3.389191000	-5.658854000	2.682591000
C	2.215388000	-5.377388000	3.340703000
C	1.020455000	-5.100481000	2.604816000
C	1.069819000	-5.084939000	1.160004000
C	2.303866000	-5.384286000	0.516788000
C	3.428533000	-5.674819000	1.257536000
C	-0.201440000	-4.853329000	3.249185000
C	-0.112814000	-4.776434000	0.430258000
C	-1.361596000	-4.609882000	1.098495000
C	-1.389023000	-4.596817000	2.542179000
C	-2.624356000	-4.329702000	3.213293000
H	-2.631351000	-4.308624000	4.297790000
C	-3.775833000	-4.101376000	2.496250000
C	-3.751999000	-4.142149000	1.071120000
C	-2.579021000	-4.387477000	0.390367000
H	-1.179249000	-2.425322000	-0.952976000
H	4.292811000	-5.874737000	3.240990000
H	2.171715000	-5.367317000	4.424687000
H	2.346081000	-5.379686000	-0.566446000
H	4.358495000	-5.911237000	0.755910000
H	-0.119663000	-4.986568000	-0.638749000
H	-4.708786000	-3.896349000	3.008012000
H	-4.670662000	-3.976652000	0.520206000
H	-2.573930000	-4.430947000	-0.694452000
H	-0.004404000	-3.197469000	0.065701000
H	-0.230405000	-4.854435000	4.334762000

^{5}TS2$_{reb}$

Mn	-0.265758000	-0.187987000	0.266764000
N	-0.648849000	0.593155000	2.463944000
C	0.477419000	0.315737000	3.479516000
C	1.696622000	1.186736000	3.121802000
H	2.051166000	0.954372000	2.117060000
H	1.494341000	2.257772000	3.194925000
H	2.502984000	0.959348000	3.824988000
C	0.006818000	0.626803000	4.915989000
H	-0.798517000	-0.042323000	5.232620000
H	-0.336224000	1.660399000	5.025743000
H	0.845133000	0.483466000	5.604183000
C	0.900633000	-1.158615000	3.377425000
H	1.745688000	-1.326552000	4.052226000
H	1.220810000	-1.400552000	2.363169000
H	0.105071000	-1.842172000	3.685981000
C	-1.912913000	-0.160456000	2.763078000
H	-2.416571000	0.223521000	3.656340000
H	-1.648337000	-1.200911000	2.953004000
C	-2.855557000	-0.082286000	1.587722000
C	-4.243194000	-0.104469000	1.699388000
H	-4.706447000	-0.201446000	2.672504000
C	-5.016857000	0.014017000	0.538311000
H	-6.097400000	-0.018990000	0.602295000
C	-4.395484000	0.213827000	-0.698042000
H	-4.978854000	0.357546000	-1.598215000
C	-3.002116000	0.250026000	-0.759751000
N	-2.274085000	0.044415000	0.365694000
C	-2.243929000	0.645065000	-2.000790000
H	-2.797965000	0.357042000	-2.895315000
H	-2.208393000	1.740221000	-2.012507000
N	-0.837564000	0.121165000	-2.024682000
C	-0.674337000	-1.091126000	-2.966496000
C	-0.738660000	-0.644805000	-4.442926000
H	-1.651080000	-0.081241000	-4.664028000
H	-0.734818000	-1.531574000	-5.083663000
H	0.124474000	-0.033594000	-4.720628000
C	0.672262000	-1.775703000	-2.681661000
H	1.527046000	-1.115072000	-2.840063000
H	0.786323000	-2.621233000	-3.367902000
H	0.712800000	-2.145451000	-1.656837000
C	-1.800172000	-2.105217000	-2.688137000
H	-1.803609000	-2.415701000	-1.644377000
H	-1.621011000	-2.998031000	-3.293503000
H	-2.792316000	-1.731739000	-2.950453000
C	0.129162000	1.229398000	-2.337485000
H	-0.096430000	1.707489000	-3.295836000
H	1.127073000	0.793396000	-2.406773000
C	0.098859000	2.273249000	-1.247415000
C	0.301065000	3.634232000	-1.462329000
H	0.518580000	3.999241000	-2.457642000
C	0.202858000	4.511464000	-0.374720000
H	0.371683000	5.571600000	-0.517710000
C	-0.150759000	4.024012000	0.886912000
H	-0.278622000	4.692615000	1.728538000
C	-0.365581000	2.654053000	1.047684000
N	-0.185798000	1.821199000	-0.000846000
C	-0.934476000	2.057113000	2.311190000
H	-0.598344000	2.624167000	3.180439000
H	-2.020304000	2.197232000	2.264842000
O	1.535327000	-0.322668000	0.105180000
H	1.986397000	0.531337000	0.057009000
O	-0.584418000	-2.114521000	0.535108000
I	3.771960000	-2.074300000	-0.398035000
C	5.291671000	-3.536420000	-0.569967000
C	5.327895000	-4.360919000	-1.698176000
H	4.627139000	-4.209012000	-2.511739000
C	6.257590000	-5.401213000	-1.757756000
H	6.281700000	-6.045488000	-2.631353000
C	7.143552000	-5.618188000	-0.699982000
H	7.859981000	-6.431990000	-0.747914000
C	7.104823000	-4.783559000	0.419036000
H	7.790636000	-4.945403000	1.245055000
C	6.179162000	-3.739812000	0.490346000
H	6.138559000	-3.106841000	1.370016000
C	3.040333000	-4.944378000	1.942701000
C	1.807223000	-4.320872000	2.099248000
C	0.824630000	-4.420070000	1.090612000
C	1.096315000	-5.153265000	-0.091178000
C	2.346448000	-5.760882000	-0.239077000
C	3.310002000	-5.663479000	0.768994000
C	-0.501561000	-3.875511000	1.302248000
C	0.040145000	-5.279517000	-1.158108000
C	-1.374088000	-5.117587000	-0.659092000
C	-1.623413000	-4.442256000	0.558366000
C	-2.948438000	-4.246720000	1.005597000
H	-3.125887000	-3.723763000	1.939905000

C	-4.018388000	-4.717379000	0.249933000
C	-3.771127000	-5.386441000	-0.959425000
C	-2.461264000	-5.585680000	-1.406079000
H	-1.511437000	-2.213413000	0.266092000
H	3.792255000	-4.869758000	2.719064000
H	1.585291000	-3.771642000	3.006395000
H	2.564349000	-6.322251000	-1.141124000
H	4.273976000	-6.140495000	0.637218000
H	0.146801000	-6.234984000	-1.682125000
H	-5.035131000	-4.569255000	0.593742000
H	-4.601320000	-5.756190000	-1.550677000
H	-2.280827000	-6.105779000	-2.340735000
H	0.223746000	-4.505197000	-1.920986000
H	-0.714652000	-3.510828000	2.299344000

^{5}PC$_{H}$

Mn	0.194165000	-0.019837000	-0.203707000
N	0.179855000	0.281293000	2.127870000
C	1.543189000	0.297625000	2.855862000
C	2.292137000	1.586687000	2.469058000
H	2.413284000	1.651419000	1.386421000
H	1.800876000	2.493318000	2.829820000
H	3.289288000	1.556334000	2.917435000
C	1.339927000	0.237772000	4.385219000
H	0.927618000	-0.725000000	4.700583000
H	0.685255000	1.034288000	4.752252000
H	2.310439000	0.357394000	4.875661000
C	2.391636000	-0.901690000	2.402647000
H	3.332860000	-0.888344000	2.961339000
H	2.631020000	-0.823793000	1.343066000
H	1.916444000	-1.865483000	2.595820000
C	-0.668210000	-0.902873000	2.510750000
H	-1.013724000	-0.838532000	3.546984000
H	-0.057661000	-1.802474000	2.419034000
C	-1.857111000	-0.995461000	1.591166000
C	-3.114117000	-1.443837000	1.980526000
H	-3.276581000	-1.781224000	2.995306000
C	-4.154008000	-1.444471000	1.043058000
H	-5.134164000	-1.807059000	1.322654000
C	-3.935518000	-0.942142000	-0.242692000
H	-4.739013000	-0.894813000	-0.965904000
C	-2.668772000	-0.466866000	-0.578670000
N	-1.656296000	-0.554845000	0.319772000
C	-2.391918000	0.300106000	-1.843879000
H	-3.008584000	-0.074058000	-2.661608000
H	-2.721717000	1.329793000	-1.667383000
N	-0.934408000	0.325950000	-2.217306000
C	-0.618461000	-0.647106000	-3.376345000
C	-1.222578000	-0.128842000	-4.698631000
H	-2.296011000	0.067916000	-4.616818000
H	-1.085613000	-0.889132000	-5.473144000
H	-0.726216000	0.784135000	-5.039617000
C	0.903584000	-0.803376000	-3.532559000
H	1.410808000	0.142120000	-3.737158000
H	1.095241000	-1.469120000	-4.379599000
H	1.348796000	-1.247180000	-2.643404000
C	-1.212734000	-2.024576000	-3.029350000
H	-0.859825000	-2.373520000	-2.058484000
H	-0.884347000	-2.747462000	-3.781391000
H	-2.304530000	-2.034324000	-3.024354000
C	-0.499237000	1.738523000	-2.500846000
H	-1.113350000	2.200459000	-3.279962000
H	0.530741000	1.708180000	-2.859481000
C	-0.581390000	2.573501000	-1.243414000
C	-0.859701000	3.937704000	-1.216980000
H	-1.015199000	4.479098000	-2.141119000
C	-0.947164000	4.582469000	0.024093000
H	-1.148643000	5.645720000	0.066302000
C	-0.811923000	3.851114000	1.209019000
H	-0.923433000	4.327643000	2.174531000
C	-0.555161000	2.481430000	1.130813000
N	-0.401542000	1.903906000	-0.079745000
C	-0.583911000	1.561315000	2.328231000
H	-0.246827000	2.092015000	3.218980000
H	-1.635793000	1.312772000	2.506640000
O	1.864150000	0.390179000	-0.732073000
H	2.119614000	1.322222000	-0.720206000
O	0.772525000	-1.976076000	-0.132500000
I	4.153169000	-1.874115000	-1.153440000
C	5.105024000	-3.687231000	-0.607298000
C	4.856084000	-4.844715000	-1.347254000
H	4.221674000	-4.815926000	-2.225857000
C	5.429394000	-6.048470000	-0.929243000
H	5.234121000	-6.954044000	-1.494681000
C	6.242955000	-6.088011000	0.204637000
H	6.682631000	-7.026778000	0.524999000
C	6.490980000	-4.918005000	0.925441000
H	7.123575000	-4.942081000	1.807058000
C	5.920510000	-3.706753000	0.525990000
H	6.099007000	-2.802378000	1.096165000
C	1.980505000	-4.705025000	2.880469000
C	0.616463000	-4.610608000	3.024072000
C	-0.249855000	-4.705647000	1.886679000
C	0.342536000	-4.889279000	0.576341000
C	1.768576000	-5.000033000	0.474377000
C	2.563129000	-4.910582000	1.591288000
C	-1.646362000	-4.627154000	1.999407000
C	-0.494209000	-4.913488000	-0.555798000
C	-1.892214000	-4.811227000	-0.440806000
C	-2.484079000	-4.692456000	0.874588000
C	-3.909428000	-4.633213000	0.984030000
H	-4.350055000	-4.559857000	1.973086000
C	-4.703711000	-4.660894000	-0.137464000
C	-4.118329000	-4.750064000	-1.436802000
C	-2.753600000	-4.828863000	-1.583848000
H	0.263365000	-2.806063000	-0.228384000
H	2.626592000	-4.629009000	3.747909000
H	0.170763000	-4.464170000	4.002806000
H	2.210353000	-5.152370000	-0.504665000
H	3.639338000	-4.992944000	1.500070000
H	-0.050452000	-5.042333000	-1.539210000
H	-5.782816000	-4.610585000	-0.043323000
H	-4.761836000	-4.764168000	-2.309479000
H	-2.306436000	-4.911882000	-2.568961000
H	1.708822000	-2.166265000	-0.358034000
H	-2.090743000	-4.512940000	2.983773000

^{3}PC$_{H}$

Mn	0.138132000	0.018777000	-0.125137000
N	0.064884000	0.291575000	2.104614000
C	1.417044000	0.304358000	2.881211000
C	2.187636000	1.581270000	2.501839000
H	2.342482000	1.624884000	1.422853000
H	1.696434000	2.497224000	2.837943000
H	3.168788000	1.543607000	2.983604000
C	1.148112000	0.266296000	4.401402000
H	0.733003000	-0.694569000	4.716929000
H	0.480497000	1.064811000	4.737596000
H	2.102151000	0.398410000	4.919441000
C	2.265043000	-0.911123000	2.481758000

H	3.170046000	-0.909875000	3.097377000
H	2.566772000	-0.838372000	1.439206000
H	1.761827000	-1.864648000	2.649271000
C	-0.825242000	-0.871593000	2.514276000
H	-1.170006000	-0.748578000	3.543570000
H	-0.229477000	-1.783157000	2.460190000
C	-2.000077000	-0.956588000	1.574060000
C	-3.276569000	-1.389442000	1.915358000
H	-3.486078000	-1.728521000	2.921110000
C	-4.275082000	-1.377469000	0.932569000
H	-5.269271000	-1.732503000	1.169581000
C	-3.997864000	-0.881575000	-0.345446000
H	-4.769377000	-0.831063000	-1.102597000
C	-2.710396000	-0.423394000	-0.624852000
N	-1.742818000	-0.516690000	0.315796000
C	-2.335816000	0.325110000	-1.875909000
H	-2.901709000	-0.034024000	-2.734491000
H	-2.629809000	1.368975000	-1.729971000
N	-0.843964000	0.298430000	-2.169801000
C	-0.504169000	-0.726911000	-3.288100000
C	-1.030104000	-0.218829000	-4.648011000
H	-2.097023000	0.021116000	-4.624987000
H	-0.888668000	-1.010817000	-5.388879000
H	-0.479699000	0.659656000	-4.995038000
C	1.015367000	-0.933383000	-3.373298000
H	1.562303000	-0.007486000	-3.562681000
H	1.219938000	-1.613536000	-4.205613000
H	1.405386000	-1.380594000	-2.462338000
C	-1.167916000	-2.069222000	-2.934482000
H	-0.901119000	-2.390870000	-1.928324000
H	-0.807813000	-2.829101000	-3.633398000
H	-2.256487000	-2.044713000	-3.013253000
C	-0.364936000	1.699541000	-2.498132000
H	-0.942497000	2.125078000	-3.322387000
H	0.677282000	1.629288000	-2.810076000
C	-0.491067000	2.569142000	-1.272913000
C	-0.757679000	3.935095000	-1.279748000
H	-0.861430000	4.462569000	-2.218843000
C	-0.902667000	4.600855000	-0.055749000
H	-1.094360000	5.666383000	-0.039913000
C	-0.838499000	3.885398000	1.144471000
H	-0.994265000	4.377132000	2.095904000
C	-0.594791000	2.512929000	1.100548000
N	-0.378684000	1.906470000	-0.091702000
C	-0.690712000	1.599732000	2.291404000
H	-0.364240000	2.102989000	3.199890000
H	-1.748811000	1.360616000	2.433690000
O	1.884493000	0.364503000	-0.603116000
H	2.104145000	1.298780000	-0.725876000
O	0.734177000	-1.927578000	-0.030957000
I	4.136473000	-1.803985000	-0.974542000
C	5.115996000	-3.663087000	-0.698501000
C	4.799869000	-4.736101000	-1.535073000
H	4.102614000	-4.611334000	-2.355964000
C	5.387465000	-5.980268000	-1.292192000
H	5.140399000	-6.819143000	-1.935118000
C	6.281284000	-6.144993000	-0.232568000
H	6.731816000	-7.114602000	-0.047521000
C	6.595456000	-5.059832000	0.588133000
H	7.290799000	-5.180903000	1.412770000
C	6.012591000	-3.810202000	0.362470000
H	6.244834000	-2.972975000	1.010853000
C	2.059840000	-4.667303000	2.913280000
C	0.697765000	-4.584285000	3.081759000
C	-0.188265000	-4.681664000	1.959820000
C	0.382132000	-4.862005000	0.639346000
C	1.807002000	-4.955105000	0.510567000
C	2.621186000	-4.859224000	1.612820000
C	-1.583258000	-4.606376000	2.096577000
C	-0.474665000	-4.898745000	-0.477092000
C	-1.871024000	-4.807136000	-0.337968000
C	-2.440426000	-4.680333000	0.986926000
C	-3.864106000	-4.626988000	1.120550000
H	-4.287773000	-4.543728000	2.116167000
C	-4.677572000	-4.671261000	0.013830000
C	-4.114726000	-4.770538000	-1.294930000
C	-2.752528000	-4.841297000	-1.465507000
H	0.228694000	-2.756898000	-0.133605000
H	2.720532000	-4.588512000	3.769412000
H	0.268816000	-4.444031000	4.068880000
H	2.232755000	-5.096422000	-0.477157000
H	3.697021000	-4.924009000	1.499657000
H	-0.047028000	-5.025604000	-1.467764000
H	-5.755138000	-4.626031000	0.126422000
H	-4.773570000	-4.797918000	-2.155803000
H	-2.322417000	-4.931500000	-2.457580000
H	1.666930000	-2.100372000	-0.279573000
H	-2.011030000	-4.488807000	3.087996000

$^{5}PC_{reb}$

Mn	-0.012375000	-0.821640000	0.398305000
N	-0.891180000	0.384938000	2.247438000
C	-0.303304000	0.120335000	3.651833000
C	1.113875000	0.716292000	3.717223000
H	1.736836000	0.322044000	2.914488000
H	1.125831000	1.807447000	3.670898000
H	1.570338000	0.425452000	4.667552000
C	-1.193597000	0.748901000	4.744540000
H	-2.163795000	0.249357000	4.816689000
H	-1.363073000	1.817563000	4.579655000
H	-0.694371000	0.640581000	5.711806000
C	-0.188080000	-1.393259000	3.877782000
H	0.207379000	-1.572211000	4.881848000
H	0.503637000	-1.827191000	3.154076000
H	-1.154383000	-1.898412000	3.818967000
C	-2.330638000	-0.038664000	2.129888000
H	-2.989930000	0.588748000	2.737333000
H	-2.416830000	-1.061074000	2.500273000
C	-2.770878000	0.035646000	0.689660000
C	-4.066309000	0.346097000	0.287599000
H	-4.837930000	0.509251000	1.028480000
C	-4.339985000	0.463632000	-1.080952000
H	-5.345206000	0.689835000	-1.414156000
C	-3.306695000	0.334187000	-2.013287000
H	-3.488719000	0.477482000	-3.070413000
C	-2.018585000	0.040182000	-1.564184000
N	-1.797773000	-0.163131000	-0.241331000
C	-0.811969000	0.073547000	-2.463028000
H	-1.093102000	-0.208907000	-3.477670000
H	-0.487962000	1.118457000	-2.521909000
N	0.333271000	-0.758343000	-1.958208000

C	0.458947000	-2.087451000	-2.733205000
C	0.800571000	-1.842282000	-4.219593000
H	0.030111000	-1.273762000	-4.748542000
H	0.889664000	-2.812635000	-4.717666000
H	1.757530000	-1.325539000	-4.334852000
C	1.571953000	-2.930516000	-2.097483000
H	2.563456000	-2.574418000	-2.380519000
H	1.483652000	-3.962493000	-2.444353000
H	1.502519000	-2.932670000	-1.011143000
C	-0.879218000	-2.836153000	-2.620672000
H	-1.113482000	-3.041078000	-1.576650000
H	-0.790772000	-3.792775000	-3.142975000
H	-1.712407000	-2.292154000	-3.072681000
C	1.617251000	0.024337000	-1.962153000
H	1.883000000	0.370695000	-2.965426000
H	2.410079000	-0.639131000	-1.615242000
C	1.505555000	1.212047000	-1.041882000
C	2.119857000	2.441384000	-1.265792000
H	2.748158000	2.582407000	-2.135065000
C	1.894493000	3.483587000	-0.357992000
H	2.378762000	4.441261000	-0.502881000
C	1.012524000	3.301321000	0.711749000
H	0.784534000	4.111639000	1.392167000
C	0.396370000	2.059732000	0.875811000
N	0.704211000	1.045142000	0.038731000
C	-0.735471000	1.823473000	1.844220000
H	-0.634186000	2.474448000	2.712977000
H	-1.651445000	2.142361000	1.335107000
O	1.552823000	-1.457734000	0.978988000
H	2.240946000	-0.805925000	1.183027000
O	-0.936262000	-2.642172000	0.750891000
I	4.451744000	0.875923000	1.511937000
C	4.820266000	-0.229605000	-0.260652000
C	5.106052000	0.454972000	-1.443422000
H	5.162730000	1.536771000	-1.458693000
C	5.314880000	-0.281174000	-2.612648000
H	5.531763000	0.242925000	-3.537800000
C	5.248723000	-1.675819000	-2.591643000
H	5.411608000	-2.241193000	-3.503074000
C	4.967439000	-2.342032000	-1.397158000
H	4.902981000	-3.424986000	-1.374969000
C	4.743866000	-1.623526000	-0.220310000
H	4.509060000	-2.144110000	0.700346000
C	2.728801000	-5.318358000	0.300374000
C	1.774022000	-4.586597000	1.008256000
C	0.408111000	-4.744396000	0.728232000
C	-0.012232000	-5.662771000	-0.252037000
C	0.954594000	-6.391142000	-0.959583000
C	2.315965000	-6.218493000	-0.691226000
C	-0.610987000	-3.960287000	1.493881000
C	-1.486778000	-5.869812000	-0.517649000
C	-2.363618000	-5.562247000	0.676991000
C	-1.923463000	-4.664559000	1.670142000
C	-2.730104000	-4.397445000	2.788892000
H	-2.371327000	-3.722797000	3.559164000
C	-3.979782000	-5.003904000	2.919675000
C	-4.426840000	-5.886763000	1.926960000
C	-3.621295000	-6.165911000	0.819514000
H	-1.850837000	-2.715616000	0.427023000
H	3.783178000	-5.192560000	0.520673000
H	2.080883000	-3.868925000	1.759389000
H	0.636741000	-7.104707000	-1.713162000
H	3.051342000	-6.792936000	-1.244190000
H	-1.663046000	-6.898838000	-0.848102000
H	-4.596647000	-4.797152000	3.786755000
H	-5.395437000	-6.365103000	2.022860000
H	-3.963730000	-6.864177000	0.062555000
H	-1.796353000	-5.234714000	-1.362336000
H	-0.206997000	-3.608765000	2.438160000

E. Thioanisole sulfoxidation by the PhIO molecule.

RC_0

I	3.385889000	-3.046892000	0.112940000
O	4.676099000	-4.132496000	-0.704841000
C	4.290093000	-1.133531000	-0.150068000
C	5.654344000	-1.144000000	-0.418475000
C	3.533835000	0.033268000	-0.108993000
C	6.288485000	0.078557000	-0.651640000
C	4.188655000	1.245525000	-0.338348000
C	5.557500000	1.268530000	-0.610496000
H	6.186767000	-2.088795000	-0.470143000
H	2.463842000	0.010582000	0.067094000
H	7.351547000	0.096433000	-0.870673000
H	3.616237000	2.166393000	-0.339299000
H	6.052331000	2.214268000	-0.804832000
S	2.763561000	-1.630531000	-3.463134000
C	4.459545000	-2.322343000	-3.482104000
C	3.035713000	0.132238000	-3.538069000
C	1.987803000	0.962140000	-3.095918000
C	2.132322000	2.350960000	-3.115442000
C	3.326123000	2.932279000	-3.563562000
C	4.366304000	2.108865000	-4.006624000
C	4.223269000	0.716216000	-4.005836000
H	4.436008000	-3.248180000	-2.900767000
H	4.806455000	-2.509540000	-4.499763000
H	5.138188000	-1.635808000	-2.974201000
H	1.071404000	0.517806000	-2.720844000
H	1.318015000	2.977792000	-2.766748000
H	3.442103000	4.010631000	-3.566676000
H	5.294053000	2.546835000	-4.360451000
H	5.036470000	0.098146000	-4.365559000

TS_0

I	2.764898000	-1.741943000	-0.198840000
O	3.021383000	-0.107121000	-1.498682000
C	4.536678000	-1.365478000	0.918666000
C	5.155729000	-0.137730000	0.710745000
C	5.036371000	-2.317848000	1.804083000
C	6.324900000	0.145577000	1.420912000
C	6.207705000	-2.016196000	2.504858000
C	6.850777000	-0.790503000	2.314784000
H	4.716787000	0.558772000	0.004623000
H	4.537525000	-3.270496000	1.947086000
H	6.822150000	1.099372000	1.272880000
H	6.615672000	-2.745743000	3.197517000
H	7.760509000	-0.566316000	2.862489000
S	3.828425000	1.478846000	-2.839805000
C	5.335419000	0.493016000	-3.078348000
C	3.000865000	1.344624000	-4.407353000
C	1.892075000	2.191194000	-4.609498000
C	1.168621000	2.128382000	-5.801510000
C	1.540721000	1.226869000	-6.808515000
C	2.641490000	0.386643000	-6.609110000
C	3.369984000	0.437003000	-5.414731000
H	5.908718000	0.588624000	-2.154976000
H	5.072060000	-0.555829000	-3.215767000
H	5.925764000	0.866562000	-3.917413000
H	1.603376000	2.896092000	-3.836343000
H	0.318189000	2.786222000	-5.945840000

H	0.979857000	1.181963000	-7.735514000
H	2.937854000	-0.314700000	-7.382040000
H	4.214093000	-0.226813000	-5.280537000

PC_0

I	1.971353000	0.812586000	1.030961000
O	4.364098000	3.048950000	-1.798079000
C	3.730836000	-0.311282000	0.620936000
C	4.796442000	0.304131000	-0.037800000
C	3.788129000	-1.645045000	1.028304000
C	5.950621000	-0.447557000	-0.278330000
C	4.948451000	-2.379125000	0.769734000
C	6.031004000	-1.782695000	0.120852000
H	4.729530000	1.332035000	-0.381692000
H	2.949546000	-2.107101000	1.536316000
H	6.793431000	0.021424000	-0.777146000
H	5.000486000	-3.417638000	1.081560000
H	6.932397000	-2.355383000	-0.072326000
S	4.659597000	2.820873000	-3.277361000
C	6.241500000	1.903724000	-3.371912000
C	3.589830000	1.457935000	-3.824283000
C	2.712113000	0.893177000	-2.897792000
C	1.869491000	-0.149589000	-3.299685000
C	1.905578000	-0.610199000	-4.621396000
C	2.779882000	-0.023478000	-5.547427000
C	3.624425000	1.020219000	-5.153991000
H	7.025022000	2.561753000	-2.990294000
H	6.170700000	1.000599000	-2.763695000
H	6.446702000	1.646641000	-4.413535000
H	2.693500000	1.270959000	-1.883965000
H	1.189484000	-0.596549000	-2.582301000
H	1.252045000	-1.417825000	-4.932682000
H	2.802226000	-0.375121000	-6.573103000
H	4.294772000	1.479275000	-5.873856000

F. C-H bond activation reaction of 9, 10-dihydroanthracene by the PhIO molecule.

RC_0'

I	3.814215000	-0.999765000	-8.832726000
O	5.598107000	-1.243235000	-9.346845000
C	3.272092000	-3.033671000	-8.465240000
C	1.993627000	-3.369325000	-8.030512000
C	4.285367000	-3.963614000	-8.651369000
C	1.729202000	-4.717761000	-7.774537000
C	3.997725000	-5.304804000	-8.390982000
C	2.725788000	-5.679826000	-7.954667000
H	1.223215000	-2.618340000	-7.887452000
H	5.266634000	-3.633636000	-8.977393000
H	0.741479000	-5.010157000	-7.432332000
H	4.775854000	-6.050356000	-8.513634000
H	2.511887000	-6.723048000	-7.746239000
C	6.058587000	1.694596000	-4.686659000
C	6.616812000	0.612002000	-5.372349000
C	5.926221000	-0.605218000	-5.489111000
C	4.656112000	-0.734768000	-4.895690000
C	4.106272000	0.352329000	-4.198057000
C	4.795374000	1.564078000	-4.094790000
C	6.529209000	-1.755469000	-6.266811000
C	3.878575000	-2.026421000	-5.034706000
C	4.752885000	-3.244957000	-5.240589000
C	6.023187000	-3.113853000	-5.832451000
C	6.804978000	-4.261155000	-6.039509000
H	7.787662000	-4.156088000	-6.491005000
C	6.336204000	-5.526596000	-5.677023000
C	5.072875000	-5.655121000	-5.086418000
C	4.293693000	-4.516822000	-4.867584000
H	7.622747000	-1.723916000	-6.183999000
H	6.606059000	2.628250000	-4.607393000
H	7.600422000	0.706157000	-5.824153000
H	3.129667000	0.243414000	-3.734410000
H	4.355788000	2.395354000	-3.553202000
H	3.192339000	-1.937619000	-5.891834000
H	6.952239000	-6.403689000	-5.847647000
H	4.699637000	-6.632777000	-4.799407000
H	3.312576000	-4.611620000	-4.411200000
H	3.234568000	-2.171488000	-4.159297000
H	6.309484000	-1.614059000	-7.340493000

TS_0'

I	3.562310000	-1.218339000	-9.265731000
O	5.489384000	-1.232875000	-8.536845000
C	3.197831000	-3.200205000	-8.609014000
C	1.919464000	-3.559750000	-8.186724000
C	4.260160000	-4.099870000	-8.651891000
C	1.701756000	-4.887483000	-7.810665000
C	4.015574000	-5.423009000	-8.283995000
C	2.742676000	-5.816836000	-7.867800000
H	1.115391000	-2.832880000	-8.146336000
H	5.248548000	-3.761822000	-8.937817000
H	0.715901000	-5.188994000	-7.471214000
H	4.829564000	-6.138530000	-8.305313000
H	2.564259000	-6.846460000	-7.575385000
C	6.237217000	1.662765000	-4.480275000
C	6.751760000	0.612934000	-5.243964000
C	6.001137000	-0.560557000	-5.453710000
C	4.706426000	-0.660464000	-4.886331000
C	4.205372000	0.392834000	-4.114306000
C	4.961022000	1.554458000	-3.909453000
C	6.506728000	-1.681208000	-6.266498000
C	3.884315000	-1.897170000	-5.181683000
C	4.729919000	-3.143354000	-5.326943000
C	6.019992000	-3.018575000	-5.903179000
C	6.790520000	-4.180837000	-6.110260000
H	7.775504000	-4.087952000	-6.558522000
C	6.309043000	-5.433314000	-5.727539000
C	5.043626000	-5.546799000	-5.134124000
C	4.263210000	-4.401954000	-4.938550000
H	7.561966000	-1.625420000	-6.530380000
H	6.829127000	2.558869000	-4.323887000
H	7.743442000	0.690783000	-5.680168000
H	3.215901000	0.305301000	-3.674288000
H	4.559758000	2.364832000	-3.309905000
H	3.344473000	-1.736292000	-6.133237000
H	6.917136000	-6.318265000	-5.885398000
H	4.666763000	-6.518556000	-4.833364000
H	3.276880000	-4.488296000	-4.492415000
H	3.110365000	-2.040059000	-4.419929000
H	5.814936000	-1.439647000	-7.462684000

PC_0'

I	4.350257000	-2.822213000	-11.592029000
O	5.554777000	-1.720544000	-7.751825000

C	3.625648000	-3.462544000	-9.702200000	C	5.007148000	-3.503867000	-4.899863000
C	2.656034000	-2.698016000	-9.049283000	C	5.999039000	-3.193871000	-5.846053000
C	4.145502000	-4.623917000	-9.128612000	C	6.842429000	-4.209392000	-6.324967000
C	2.199491000	-3.117000000	-7.794811000	H	7.602071000	-3.960152000	-7.059969000
C	3.678734000	-5.026543000	-7.876310000	C	6.710717000	-5.523220000	-5.873871000
C	2.706623000	-4.279384000	-7.210498000	C	5.724879000	-5.832825000	-4.925646000
H	2.264479000	-1.794012000	-9.500706000	C	4.884888000	-4.826974000	-4.443752000
H	4.909542000	-5.199146000	-9.638040000	H	7.190769000	-1.579553000	-6.588883000
H	1.446813000	-2.527949000	-7.280301000	H	5.784958000	2.649866000	-5.020061000
H	4.096601000	-5.912306000	-7.411535000	H	6.757542000	0.824680000	-6.398243000
H	2.356452000	-4.595051000	-6.234183000	H	3.246503000	-0.221756000	-3.058979000
C	5.438729000	1.629358000	-4.895170000	H	4.016461000	2.118955000	-3.342371000
C	5.985724000	0.603203000	-5.666853000	H	3.099159000	-2.547995000	-4.934641000
C	5.552133000	-0.724142000	-5.514631000	H	7.369499000	-6.298219000	-6.250873000
C	4.553871000	-1.024257000	-4.571332000	H	5.617927000	-6.849287000	-4.561505000
C	4.011002000	0.012514000	-3.794147000	H	4.122819000	-5.064102000	-3.707056000
C	4.444018000	1.330583000	-3.953078000	H	3.818528000	-2.624566000	-3.342760000
C	6.133055000	-1.796976000	-6.408628000	H	4.629026000	-2.010755000	-7.678507000
C	4.055519000	-2.441623000	-4.397549000				
